# Evolutionary superscaffolding and chromosome anchoring to improve *Anopheles* genome assemblies

**DOI:** 10.1186/s12915-019-0728-3

**Published:** 2020-01-02

**Authors:** Robert M. Waterhouse, Sergey Aganezov, Yoann Anselmetti, Jiyoung Lee, Livio Ruzzante, Maarten J. M. F. Reijnders, Romain Feron, Sèverine Bérard, Phillip George, Matthew W. Hahn, Paul I. Howell, Maryam Kamali, Sergey Koren, Daniel Lawson, Gareth Maslen, Ashley Peery, Adam M. Phillippy, Maria V. Sharakhova, Eric Tannier, Maria F. Unger, Simo V. Zhang, Max A. Alekseyev, Nora J. Besansky, Cedric Chauve, Scott J. Emrich, Igor V. Sharakhov

**Affiliations:** 1 Department of Ecology and Evolution, University of Lausanne, and Swiss Institute of Bioinformatics, 1015 Lausanne, Switzerland; 2 Department of Computer Science, Princeton University, Princeton, NJ 08450 USA; 3 Department of Computer Science, Johns Hopkins University, Baltimore, MD 21218 USA; 4 ISEM, Univ Montpellier, CNRS, EPHE, IRD, Montpellier, France; 5 The Interdisciplinary PhD Program in Genetics, Bioinformatics, and Computational Biology, Virginia Polytechnic Institute and State University, Blacksburg, VA 24061 USA; 6 Department of Entomology, Virginia Polytechnic Institute and State University, Blacksburg, VA 24061 USA; 7 Departments of Biology and Computer Science, Indiana University, Bloomington, IN 47405 USA; 8 Centers for Disease Control and Prevention, Atlanta, GA 30329 USA; 9 Department of Medical Entomology and Parasitology, Faculty of Medical Sciences, Tarbiat Modares University, Tehran, Iran; 10 Genome Informatics Section, Computational and Statistical Genomics Branch, National Human Genome Research Institute, National Institutes of Health, Bethesda, MD 20892 USA; 11 European Molecular Biology Laboratory, European Bioinformatics Institute, Wellcome Genome Campus, Hinxton, CB10 1SD UK; 12 Laboratory of Ecology, Genetics and Environmental Protection, Tomsk State University, Tomsk, Russia 634050; 13 Laboratoire de Biométrie et Biologie Evolutive, Université Lyon 1, Unité Mixte de Recherche 5558 Centre National de la Recherche Scientifique, 69622 Villeurbanne, France; 14 Institut national de recherche en informatique et en automatique, Montbonnot, 38334 Grenoble, Rhône-Alpes France; 15 Eck Institute for Global Health and Department of Biological Sciences, University of Notre Dame, Galvin Life Sciences Building, Notre Dame, IN 46556 USA; 16 Department of Mathematics and Computational Biology Institute, George Washington University, Ashburn, VA 20147 USA; 17 Department of Mathematics, Simon Fraser University, Burnaby, British Columbia V5A 1S6 Canada; 18 Department of Electrical Engineering and Computer Science, University of Tennessee, Knoxville, TN 37996 USA

**Keywords:** Genome assembly, Gene synteny, Comparative genomics, Mosquito genomes, Orthology, Bioinformatics, Computational evolutionary biology, Chromosomes, Physical mapping

## Abstract

**Background:**

New sequencing technologies have lowered financial barriers to whole genome sequencing, but resulting assemblies are often fragmented and far from ‘finished’. Updating multi-scaffold drafts to chromosome-level status can be achieved through experimental mapping or re-sequencing efforts. Avoiding the costs associated with such approaches, comparative genomic analysis of gene order conservation (synteny) to predict scaffold neighbours (adjacencies) offers a potentially useful complementary method for improving draft assemblies.

**Results:**

We evaluated and employed 3 gene synteny-based methods applied to 21 *Anopheles* mosquito assemblies to produce consensus sets of scaffold adjacencies. For subsets of the assemblies, we integrated these with additional supporting data to confirm and complement the synteny-based adjacencies: 6 with physical mapping data that anchor scaffolds to chromosome locations, 13 with paired-end RNA sequencing (RNAseq) data, and 3 with new assemblies based on re-scaffolding or long-read data. Our combined analyses produced 20 new superscaffolded assemblies with improved contiguities: 7 for which assignments of non-anchored scaffolds to chromosome arms span more than 75% of the assemblies, and a further 7 with chromosome anchoring including an 88% anchored *Anopheles arabiensis* assembly and, respectively, 73% and 84% anchored assemblies with comprehensively updated cytogenetic photomaps for *Anopheles funestus* and *Anopheles stephensi*.

**Conclusions:**

Experimental data from probe mapping, RNAseq, or long-read technologies, where available, all contribute to successful upgrading of draft assemblies. Our evaluations show that gene synteny-based computational methods represent a valuable alternative or complementary approach. Our improved *Anopheles* reference assemblies highlight the utility of applying comparative genomics approaches to improve community genomic resources.

## Background

Reduced costs of new sequencing technologies have facilitated the rapid growth of draft genome assemblies from all kingdoms of life. Nevertheless, progressing from draft status to that of a ‘finished’ reference genome—a near-complete and near-contiguous chromosome-level assembly—remains the exclusive accomplishment of relatively few species. Chromosomal ordering and orienting of contigs or scaffolds may be achieved by experimental approaches including fluorescence in situ hybridization (FISH) [1], genetic linkage mapping [2, 3], optical (restriction site) mapping [4], or analysis of chromatin interaction frequency data [5, 6]. When resources allow, combined approaches can produce excellent results, e.g. for Brassicaceae plants [7], the three-spined stickleback [8], and the mosquitoes, *Aedes aegypti* and *Culex quinquefasciatus* [9, 10].

While many research applications may not strictly require such high-quality assemblies, improvements in contiguity, completeness, and chromosome anchoring or assignments can substantially add to the power and breadth of biological and evolutionary inferences from comparative genomics or population genetics analyses. For example, extensive contiguity and chromosome-level anchoring are clearly important when addressing questions concerning karyotype evolution or smaller-scale inversions and translocations, re-sequencing analyses of population-level samples, reconstructing rearrangement-based phylogenies, identifying and characterising genes that localise within quantitative trait loci (QTL), examining genomic sexual conflicts, or tracing drivers of speciation. In many such studies, assembly improvements were critical to enable more robust analyses, e.g. QTL analysis with rape mustard flowering-time phenotypes [11], contrasting genomic patterns of diversity between barley cultivars [12], defining rearrangements of the typical avian karyotype [13], detecting chromosome fusion events during butterfly evolution [14], characterising the ancestral lepidopteran karyotype [15], identifying the chromosomal position and structure of the male determining locus in *Ae. aegypti* [10], and characterising a melon fly genetic sexing strain as well as localising the sexing trait [16].

Available genome assemblies for anopheline mosquitoes vary considerably in contiguity and levels of chromosome anchoring. Sequencing the first mosquito genome produced an assembly for the *Anopheles gambiae* PEST strain with 8987 scaffolds spanning 278 Mbp, where physical mapping assigned 84% of the genome to chromosome arms [17]. Additional FISH mapping and orienting of scaffolds and bioinformatics analyses later facilitated an assembly update by removing haplotype scaffolds and bacterial sequences and anchoring a third of previously unmapped scaffolds to chromosomes [18]. Since then, more than 20 new assemblies have been built, several with mapping efforts that enabled at least partial chromosome anchoring. Sequencing of the *A. gambiae* Pimperena S form and *Anopheles coluzzii* (formerly *A. gambiae* M form) produced assemblies with 13,050 and 10,525 scaffolds, respectively [19]. The much smaller 174 Mbp assembly of the more distantly related neotropical vector, *Anopheles darlingi*, comprised 8233 scaffolds, but they remained unanchored [20]. Physical mapping assigned 62% of the *Anopheles stephensi* Indian strain assembly [21] and 36% of the *Anopheles sinensis* Chinese strain assembly [22, 23] to polytene chromosomes. The *Anopheles* 16 Genomes Project [24] produced assemblies ranging from a few hundred to several thousand scaffolds and used mapping data from 4 species to anchor *Anopheles funestus* (35%), *Anopheles atroparvus* (40%), *A. stephensi* SDA-500 strain (41%), and *Anopheles albimanus* (76%) genomes to chromosome arms [25]. Additional physical mapping data for *A. atroparvus* subsequently improved this initial assembly to 90% chromosome anchoring [26] and for *A. albimanus* to 98% [27].

For a genus such as *Anopheles* with already more than 20 genome assemblies available [28], multi-species comparative analyses to identify potentially neighbouring scaffolds could facilitate assembly upgrades with improved contiguities. While genomic rearrangements can and do occur, multiple homologous regions with conserved orders and orientations, i.e. regions with maintained synteny, offer an evolutionarily guided approach for assembly improvement. Specifically, employing orthologous genes as conserved markers allows for the delineation of maintained syntenic blocks that provide support for putative scaffold adjacencies. Here, we present results from applying three synteny-based computational approaches to perform evolutionarily guided assembly improvements of multiple *Anopheles* genomes. These synteny-based methods aim to identify blocks of collinear orthologues across multiple species that are then used to infer scaffold adjacencies in species where collinearity has been broken due to assembly fragmentation. They assume that multiple rearrangements over the course of evolution have eroded the collinearity of genes in extant genomes with that of the ancestral gene order. Within genomic blocks where synteny has been widely maintained, broken collinearity in one or more species delineates putative rearrangement breakpoints. Breakpoints at the extremities of contigs or scaffolds are considered more likely due to assembly fragmentation than to genomic rearrangement events, and can thus be used to infer adjacencies that repair such breakpoints. The consensus predictions offer well-supported sets of scaffold adjacencies that lead to improved assembly contiguities without the associated costs or time investments required for experimental superscaffolding. Integrating these predictions with experimental data for subsets of the anophelines supported many adjacencies and highlighted the complementarity of experimental and computational approaches. Providing support for experimental results, complementary data to enhance improvements, or independent evidence for assembly validations, these evolutionarily guided methods offer a handy set of utensils in any genome assembly toolbox—here applied to improve available genomic resources of *Anopheles* mosquitoes.

## Results

### New reference genome assemblies and chromosome maps

**Fig. 1:**
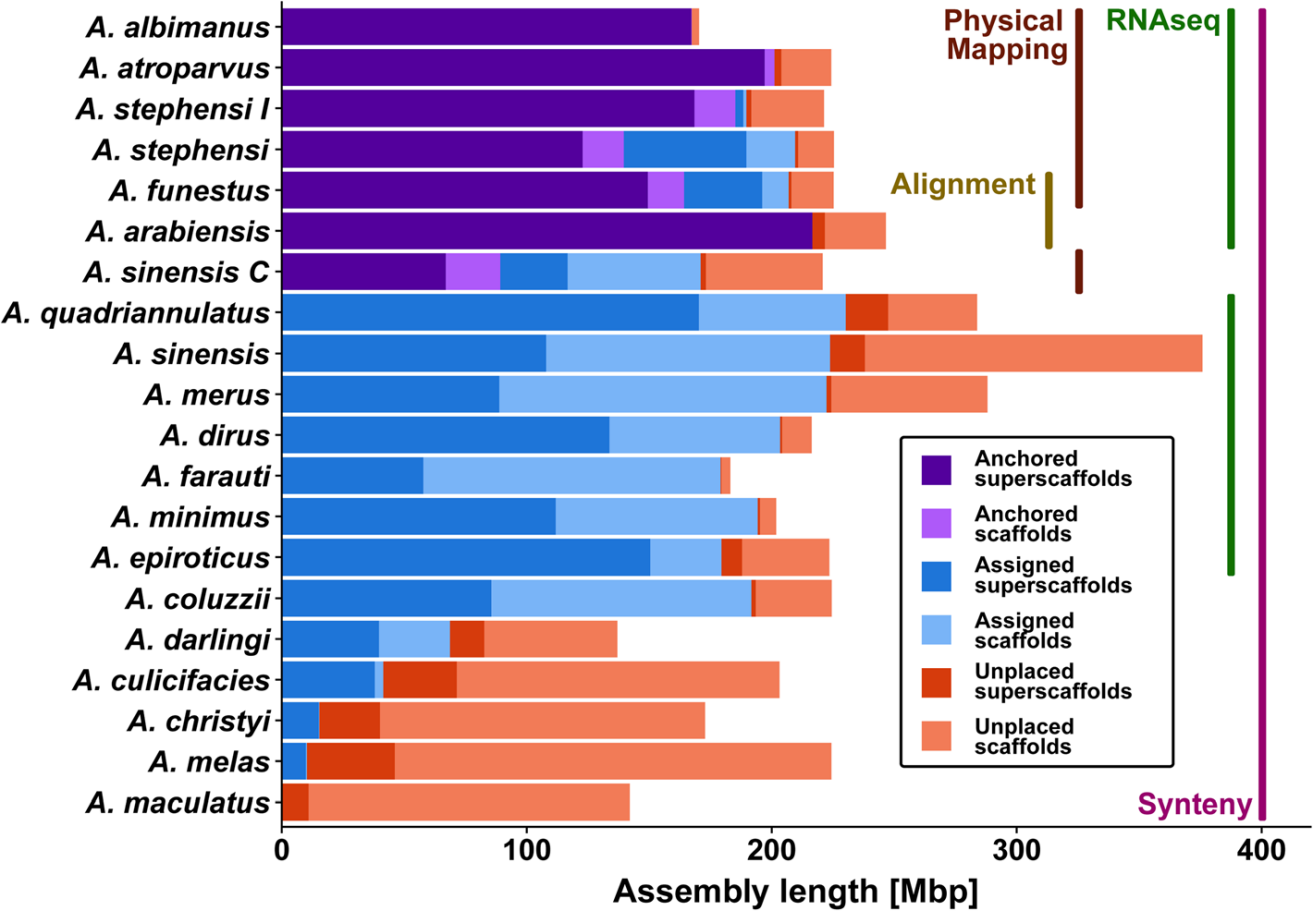
Genomic spans of scaffolds and superscaffolds with and without chromosome anchoring or arm assignments for 20 improved *Anopheles* assemblies. Consensus gene synteny-based methods were employed across the 21-assembly input dataset (also including *Anopheles gambiae*) to delineate scaffold adjacencies and build new superscaffolded assemblies with improved contiguities. These were integrated with results from additional complementary approaches for subsets of the anophelines including transcriptome (RNAseq) and genome sequencing data, whole genome alignments, and chromosome anchoring data from physical mapping of probes. Chromosome mapping data for 7 assemblies enabled anchoring of superscaffolds and scaffolds to their chromosomal locations (purple colours). Enumerating shared orthologues further enabled the assignment of non-anchored superscaffolds and scaffolds to chromosome arms (blue colours). Unplaced superscaffolds and scaffolds (orange colours) still comprise the majority of the least contiguous input assemblies, but they make up only a small proportion of the assemblies for which the available data allowed for substantial improvements to assembly contiguity and/or anchoring and/or arm assignments. Results for two strains are shown for *Anopheles sinensis*, SINENSIS and Chinese (C), and *Anopheles stephensi*, SDA-500 and Indian (I)

New genome assemblies with scaffolds and superscaffolds anchored or assigned to chromosome arms were generated by leveraging evolutionary relationships to predict scaffold adjacencies and combining these with additional experimental data for subsets of the anophelines (Fig. 1). Integrating results from 3 gene synteny-based computational approaches to build superscaffolds from all scaffold neighbours and reconciling these with the experimental datasets resulted in 20 new assemblies with variable levels of improved contiguities (Table 1), as well as chromosome mapping spanning 88% of the *Anopheles arabiensis* assembly, and updated chromosome maps for 6 other anophelines (Table 2). The synteny-based adjacencies were used to define well-supported consensus sets, which were then validated with and complemented by physical mapping and/or RNAseq and/or re-sequencing data for 14 assemblies. This followed a reconciliation workflow to integrate the different sets of scaffold adjacencies from synteny, physical mapping, RNAseq, or alignment data for each assembly (see the “Methods” section; Additional file 1: Figure S1) [29–50]. Applying this integrative approach produced updated reference assemblies with increased scaffold N50 values (a median-like metric where half the genome is assembled into scaffolds of length N50 or longer) and reduced scaffold counts (Table 1). Although superscaffold contiguity levels remain variable, the total span of scaffolds that now form part of superscaffolds comprises more than half of ten of the assemblies, ranging from 113 to 222 Mbp (Additional file 1: Figure S2).

**Table 1:** Summary statistics of the 20 input and new improved *Anopheles* assemblies

Species	Input assemblies			Approaches applied	New assemblies		
	Assembly version	Number of scaffolds	Scaffold N50 (Kbp)		Assembly version	Number of scaffolds [% reduced]	Scaffold N50 (Kbp) [fold increase]
*A. albimanus*	AalbS1	204	18,068	SYN + AGO + PHY	AalbS3^§^	203 [0.0]	33,601 [3.5]
*A. arabiensis*	AaraD1	1214	5604	SYN + AGO + ALN	AaraD2	1124 [7.5]	47,566 [8.5]
*A. atroparvus*	AatrE1	1371	9207	SYN + AGO + PHY	AatrE4^§^	1297 [5.4]	37,151 [4.0]
*A. christyi*	AchrA1	30,369	9	SYN	AchrA2	28,853 [5.0]	10 [1.1]
*A. coluzzii*	AcolM1	10,521	4437	SYN	AcolM2	10,440 [0.8]	4778 [1.1]
*A. culicifacies*	AculA1	16,162	22	SYN	AculA2	14,593 [9.7]	29 [1.3]
*A. darlingi*	AdarC3	2221	115	SYN	AdarC4	1838 [17.2]	159 [1.4]
*A. dirus*	AdirW1	1266	6906	SYN + AGO	AdirW2	1211 [4.3]	12,741 [1.8]
*A. epiroticus*	AepiE1	2673	367	SYN + AGO	AepiE2	2254 [15.7]	814 [2.2]
*A. farauti*	AfarF1	550	1197	SYN + AGO	AfarF3^§^	299 [45.6]	15,480 [12.9]
*A. funestus*	AfunF1	1392	672	SYN + AGO + PHY + PB	AfunF2	1091 [21.6]	2051 [3.1]
*A. maculatus*	AmacM1	47,797	4	SYN	AmacM2	46,342 [3.0]	4 [1.0]
*A. melas*	AmelC1	20,281	18	SYN	AmelC3^§^	18,604 [8.0]	21 [1.2]
*A. merus*	AmerM1	2753	342	SYN + AGO	AmerM3^§^	1976 [28.2]	1896 [5.5]
*A. minimus*	AminM1	678	10,313	SYN + AGO	AminM2	652 [3.8]	15,145 [1.5]
*A. quadriannulatus*	AquaS1	2823	1641	SYN + AGO	AquaS2	2617 [7.3]	2675 [1.6]
*A. sinensis*	AsinS2	10,448	579	SYN + AGO	AsinS3	10,136 [3.0]	638 [1.1]
*A. sinensis* (Chinese)	AsinC2	9592	814	SYN + PHY	AsinC3	9482 [1.1]	1025 [1.3]
*A. stephensi*	AsteS1	1110	837	SYN + AGO + PHY	AsteS2	873 [21.4]	1780 [2.1]
*A. stephensi* (Indian)	AsteI2	23,371	1591	SYN + AGO + PHY	AsteI3	23,051[1.4]	3775 [2.4]

^§^New assemblies built from adjacencies of input assembly versions via reconciliation with updated assembly versions: physical mapping improvements for AalbS2, AatrE2, and AatrE3; additional ‘Fosill’-based scaffolding for AfarF2 and AmerM2; and haplotype removal for AmelC2

**Table 2:** Summary of anchoring improvements for seven anophelines with chromosome mapping data

Assembly	Mapped scaffolds	Scaffolds added to map by			Total scaffolds added	Mapped scaffolds now with oriented neighbours	Total base pairs added	Percentage of assembly added	Total percentage of assembly anchored
		Synteny	Agouti	SYN + AGO					
*A. albimanus*	31	0	2	0	2	0	2160	0.00	98.26
*A. arabiensis*	51	4	2	0	6	0	256,948	0.10	87.84
*A. atroparvus*	46	5	7	3	9	0	870,748	0.39	89.75
*A. funestus*	202	89	45	34	100	81	26,434,544	11.73	72.91
*A. sinensis* (Chinese)	52	18	NA	NA	18	14	5,791,225	2.62	40.41
*A. stephensi*	99	102	52	45	110	77	47,779,259	21.20	61.96
*A. stephensi* (Indian)	118	76	47	33	90	92	10,975,818	4.96	83.66

The greatest reductions in the total numbers of scaffolds were achieved for some of the least contiguous input assemblies including *Anopheles christyi*, *Anopheles culicifacies*, *Anopheles maculatus*, and *Anopheles melas* (Table 1). These superscaffolded assemblies also yielded up to 24 additional ‘complete’ Benchmarking Universal Single-Copy Orthologues, as well as thousands of additional genes now with identifiable syntenic orthologues (see the “Methods” section; Additional file 1: Figure S3 and Table S1). Given the heterogeneity of the input assemblies, the relative changes highlight some of the most dramatic improvements, e.g. the *A. funestus* and *A. stephensi* (SDA-500) scaffold counts both dropped by almost 22% and the newly anchored *A. arabiensis* assembly resulted in an 8.5-fold larger N50 value (Table 1). Comparing this *A. arabiensis* assembly with that of the closely related *A. gambiae* (PEST) confirmed structural variants (Additional file 1: Figure S4) identified in the scaffold-level assembly used to explore patterns of introgression in the species complex [51] and known from previous polytene chromosome studies [52]. For the other anophelines with chromosome mapping data, the contributions of the synteny-based and/or RNAseq-based adjacencies to the numbers and genomic spans of anchored scaffolds were largest for *A. stephensi* (SDA-500) and *A. funestus*, but negligible or low for the recently updated *A. albimanus* [27], *A. atroparvus* [26], and *A. sinensis* (Chinese) [23] assemblies (Table 2). The two *A. stephensi* assemblies achieved updated assembly anchoring of 62% and 84% (both improvements of more than 20%) and *A. funestus* more than doubled to reach 73% anchored and a further 17% with chromosome arm assignments (Fig. 1; Table 2).

Summary statistics of scaffold counts and N50 values of the 20 input and improved *Anopheles* assemblies after applying synteny-based (SYN), and/or RNAseq Agouti-based (AGO), and/or alignment-based (ALN), and/or physical mapping-based (PHY), and/or PacBio sequencing-based (PB) approaches. To make the input and new scaffold N50 values directly comparable, the values for the new assemblies do not include the 100 Ns used to join scaffold adjacencies.

Summary of scaffold counts and genomic spans added to the initial chromosome maps from synteny-based (SYN) and RNAseq Agouti-based (AGO) adjacencies, and counts of chromosome-mapped scaffolds that gained oriented neighbours after incorporating the SYN and AGO scaffold adjacencies.

The seven updated assemblies with additional chromosome anchoring data (Table 2), together with the chromosome-level *A. gambiae* (PEST) genome, provided the opportunity to confidently assign non-anchored scaffolds and scaffolds from non-anchored assemblies to chromosome arms (see the “Methods” section; Additional file 1: Table S2). This resulted in total anchoring or arm assignments of 90–92% for the *A. funestus* and *A. stephensi* (SDA-500) assemblies, as well as assignments for the non-anchored assemblies of 96–97% for *A. minimus* and *Anopheles farauti* and 75% or more for a further five assemblies (Fig. 1; Additional file 2). All of the new improved *Anopheles* genome assemblies and their updated gene annotations, as well as the corresponding chromosome maps of all anchored scaffolds and superscaffolds, are available from VectorBase [53, 54].

### Synteny contributions to improved assembly contiguities

**Fig. 2:**
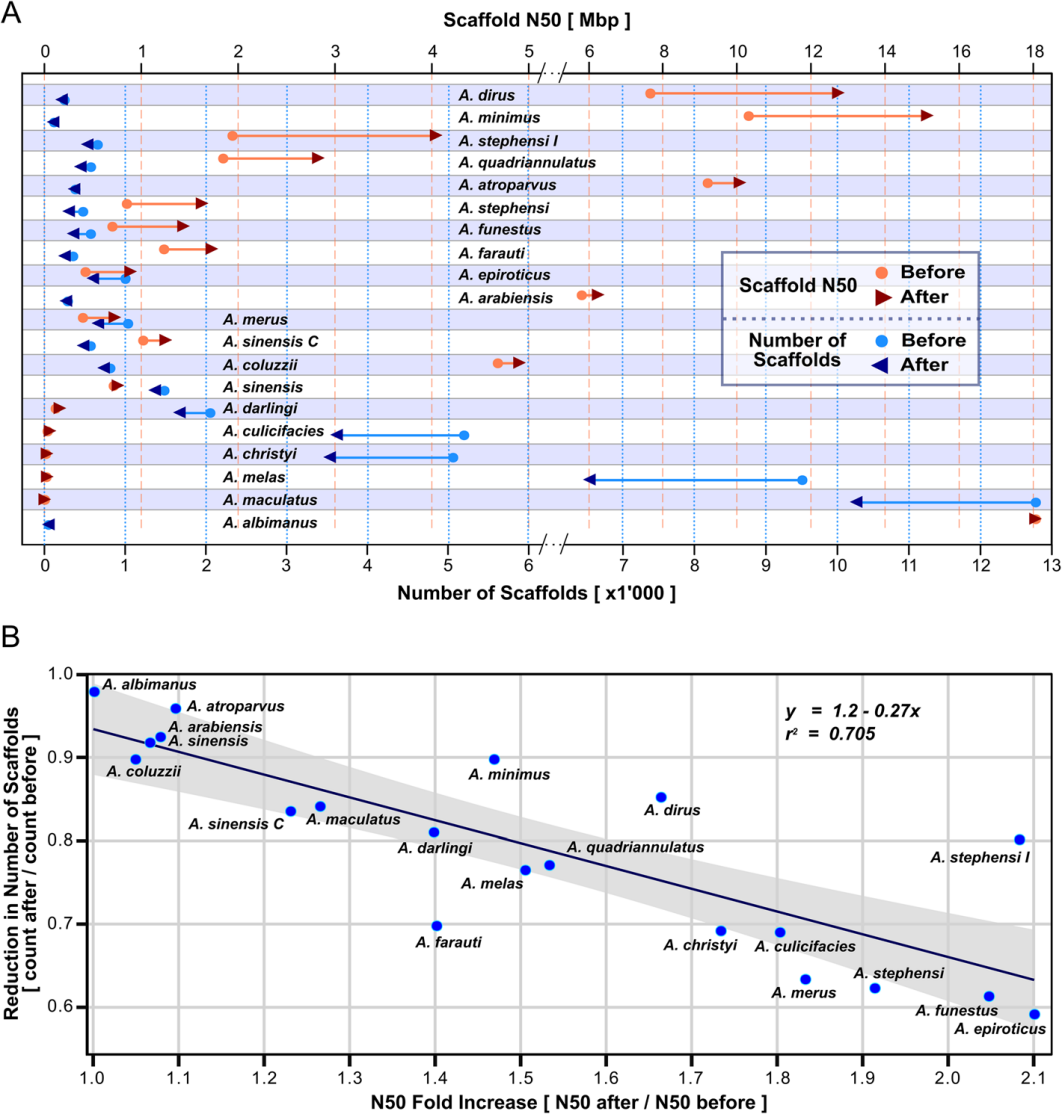
Improved genome assemblies for 20 anophelines from solely synteny-based scaffold adjacency predictions. Results from ADseq, Gos-Asm, and OrthoStitch predictions were compared to define two-way consensus adjacencies predicted by at least two of the three approaches, where the third approach did not conflict. These adjacencies were used to build new assemblies with improved contiguities, quantified by comparing before and after scaffold counts and N50 values (half the total assembly length comprises scaffolds of length N50 or longer). The counts, values, and ratios represent only scaffolds with annotated orthologous genes used as the input dataset for the scaffold adjacency predictions. To make the N50s before and after superscaffolding directly comparable, the values for the new assemblies do not include the 100 Ns used to join scaffold adjacencies. **a** Scaffold counts (blues, bottom axis) and N50 values (red/orange, top axis) are shown before (dots) and after (arrowheads) synteny-based improvements were applied. The 20 anopheline assemblies are ordered from the greatest N50 improvement at the top for *Anopheles dirus* to the smallest at the bottom for *Anopheles albimanus*. Note axis scale changes for improved visibility after N50 of 5 Mbp and scaffold count of 6000. **b** Plotting before to after ratios of scaffold counts versus N50 values (counts or N50 after/counts or N50 before superscaffolding of the adjacencies) reveals a general trend of a ~ 33% reduction in scaffold numbers resulting in a ~ 2-fold increase of N50 values. The line shows the linear regression with a 95% confidence interval in grey. Results for two strains are shown for *Anopheles sinensis*, SINENSIS and Chinese (C), and *Anopheles stephensi*, SDA-500 and Indian (I)

Applying only the synteny-based approaches to build two-way consensus sets of well-supported predicted scaffold adjacencies resulted in substantial improvements for several assemblies (Fig. 2). These employed orthologues delineated across 21 anopheline gene sets (Additional file 1: Table S3) and combined the results from two established methods, ADseq [55] and Gos-Asm [56], and a newly developed approach, OrthoStitch (see the “Methods” section; Additional file 1: Figures S5, S6 and Tables S4, S5). The two-way consensus adjacencies were required to be predicted by at least two of the approaches with no third-method conflicts. Improvements were quantified in terms of the absolute (Fig. 2a) and relative (Fig. 2b) increases in scaffold N50 values and decreases in scaffold counts, considering only scaffolds with annotated orthologous genes used as input data for the scaffold adjacency predictions.

*Anopheles dirus* and *A. minimus* achieved the greatest absolute increases in scaffold N50 values, while the greatest absolute reductions in scaffold counts were achieved for *A. christyi*, *A. culicifacies*, *A. maculatus*, and *A. melas* (Fig. 2a), reflecting the variable levels of contiguity of their input assemblies. As no physical mapping data are currently available for these species, and only *A. dirus* and *A. minimus* have supporting RNAseq data, these synteny-based adjacencies represent the only or principal resource from which to build improved assemblies. Reductions in the numbers of scaffolds that comprise each assembly varied from 1890 fewer for the rather fragmented *A. melas* assembly to just 1 fewer for the already relatively contiguous *A. albimanus* assembly. Even without large reductions in the numbers of scaffolds, when a few adjacencies bring together relatively long scaffolds, then they can lead to marked improvements in N50 values. For example, *A. dirus* and *A. minimus* improved with N50 increases of 5.1 Mbp and 4.8 Mbp and only 36 and 12 fewer scaffolds, respectively.

*Anopheles epiroticus* showed the greatest relative reduction in the number of scaffolds (40%) and achieved a 2.1-fold N50 increase, exemplifying a general trend where reducing the number of scaffolds by a third leads to a doubling of N50 values (Fig. 2b). Notable exceptions include *A. farauti*, which showed a 1.4-fold N50 increase with a 30% reduction in the number of scaffolds, while *A. dirus* and *A. stephensi* (Indian) achieved 1.66-fold and 2.08-fold N50 increases with only 14% and 19% reductions in the number of scaffolds, respectively. Using only three-way consensus adjacencies led to more conservative improvements, while employing a liberal union of all non-conflicting adjacencies resulted in a trend of a ~ 30% scaffold reduction to double N50 values (Additional file 1: Figures S7, S8). While the results clearly depend on the status of the input assemblies, the enhanced contiguities of these anopheline assemblies based solely on synteny-predicted scaffold adjacencies demonstrate that applying synteny-based approaches can achieve substantial improvements.

### Consensus adjacencies from complementary synteny-based methods

**Fig. 3:**
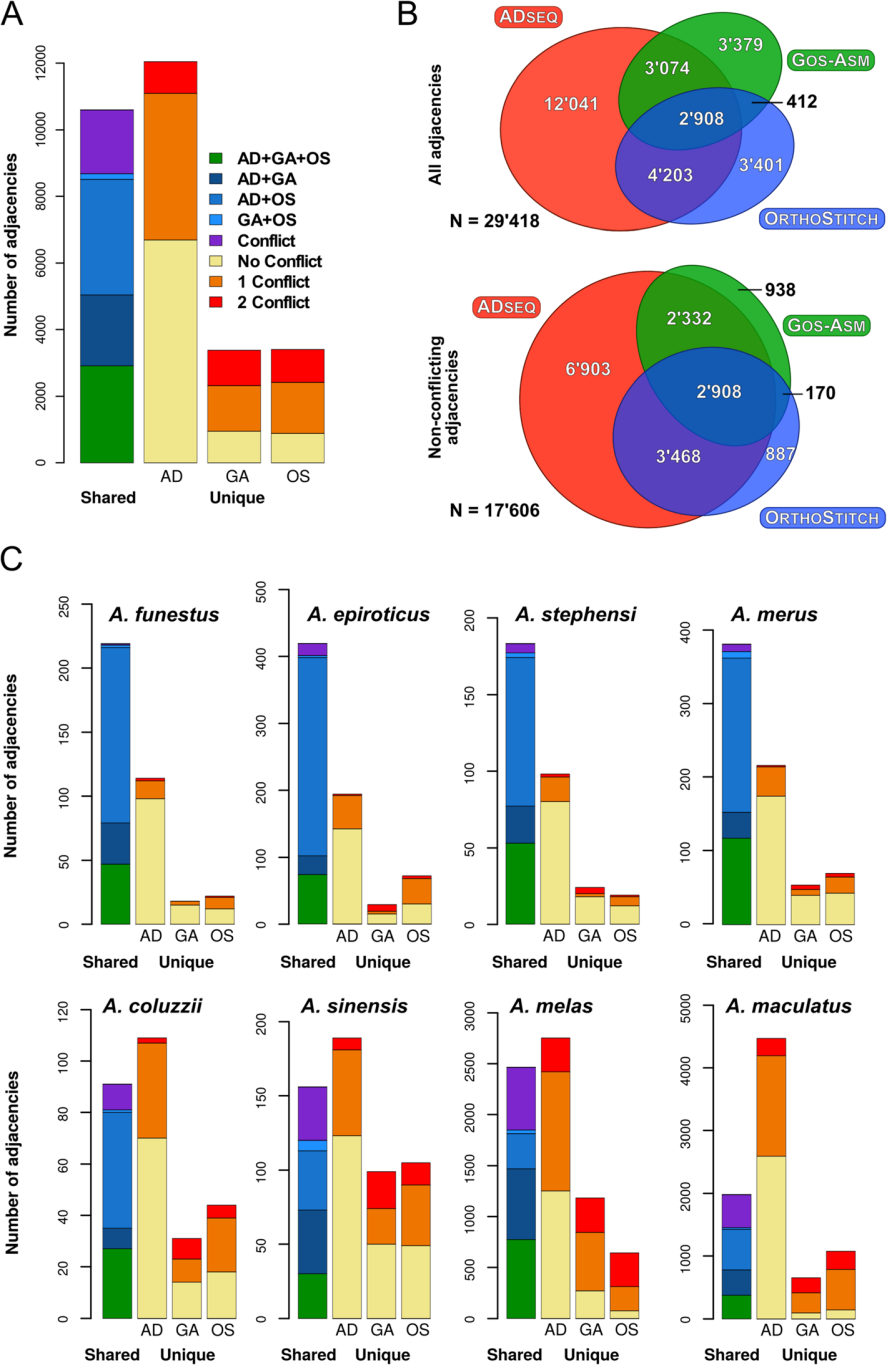
Comparisons of synteny-based scaffold adjacency predictions from ADseq (AD), Gos-Asm (GA), and OrthoStitch (OS). Bar charts show counts of predicted adjacencies (pairs of neighbouring scaffolds) that are shared amongst all three methods (green), or two methods without (blues) and with (purple) third-method conflicts, or that are unique to a single method and do not conflict (yellow) or do conflict with predictions from one (orange) or both (red) of the other methods. **a** Results of all adjacencies summed across all 20 anopheline assemblies. **b** Area-proportional Euler diagrams showing (top) the extent of the agreements amongst the three methods for all 29,418 distinct scaffold adjacencies, and (bottom) the extent of the agreements amongst the three methods for the 17,606 distinct and non-conflicting scaffold adjacencies (the liberal union sets), both summed over all 20 assemblies. **c** Individual results of adjacencies for representative anopheline assemblies, four with more than 50% agreement (top row), and four with lower levels of agreement (bottom row). Colours for each fraction are the same as in **a**, *y*-axes vary for each assembly with maxima of 120 for *Anopheles coluzzii* to 5000 for *Anopheles maculatus*. Results for *Anopheles stephensi* are for the SDA-500 strain

To systematically characterise the contributions from each of the synteny-based methods, the resulting scaffold adjacency predictions were examined with the Comparative Analysis and Merging of Scaffold Assemblies (Camsa) tool [57] (Additional file 1: Table S5). Although each of the computational methods aims to predict scaffold adjacencies based on gene collinearity, they differ in some of their underlying assumptions and in their implementations that identify, score, and infer the most likely scaffold neighbours (see the “Methods” section). Following traditional meta-assembly-like methods, the comparisons leveraged these differences to identify subsets of well-supported consensus adjacency predictions that were subsequently used for superscaffolding (Fig. 3).

For the full set of assemblies, Gos-Asm and OrthoStitch predicted about half as many oriented adjacencies compared to ADseq, with a total of almost 30,000 distinct scaffold adjacencies. More than a third were supported by at least two methods and 10% were in three-way agreement, with the larger sets of ADseq predictions resulting in a high proportion of unique adjacencies (Fig. 3; Additional file 1: Figure S9). Adjacencies in three-way agreement constituted just under a third of Gos-Asm and OrthoStitch predictions and just 13% of the more numerous ADseq predictions. From the liberal union sets of all non-conflicting adjacencies for all assemblies, the three-way agreement increased to 16.5% of the total, which increased further to 32.8% of the two-way consensus sets of adjacencies used for the synteny-based assembly improvements (Fig. 3b). Of these two-way consensus adjacencies, 98% were supported by ADseq, 74% by OrthoStitch, and 61% by Gos-Asm, and about half of Gos-Asm and OrthoStitch predictions were in three-way agreement, compared with a third for ADseq. Thus, comparing the results from the three methods and employing a two-way agreement with no third-method conflict filter resulted in greatly improved levels of adjacency agreements.

For the individual assemblies, more than half of the distinct scaffold adjacencies were in agreement for *A. epiroticus*, *Anopheles merus*, and both the *A. stephensi* assemblies, with *A. funestus* achieving the highest consistency at 58% (Fig. 3c; Additional file 1: Figure S10). Some of the most fragmented input assemblies produced some of the largest sets of distinct adjacency predictions, but the agreement amongst these predictions was generally lower than the other assemblies. For example, *A. maculatus* was the least contiguous input assembly and produced more than 8000 distinct predictions, of which only 18% showed at least two-way agreement with no conflicts (Fig. 3c; Additional file 1: Figure S10).

### Enhanced superscaffolding with physical mapping and RNA sequencing data

**Fig. 4:**
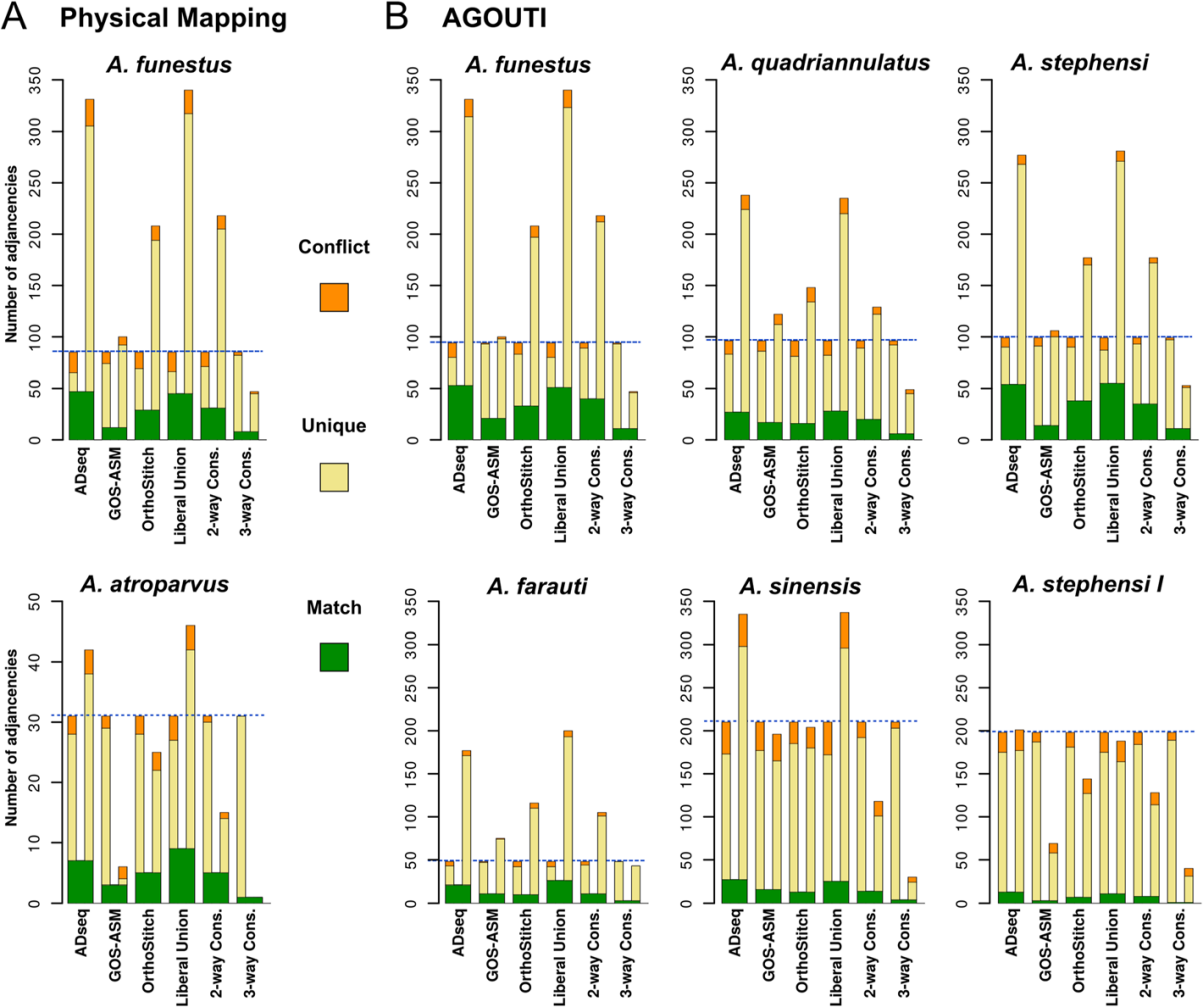
Comparisons of synteny-based scaffold adjacency predictions with physical mapping and RNA sequencing data. The bar charts show counts from each set of synteny-based scaffold adjacency predictions compared with the adjacencies from the physical mapping (**a**) or RNAseq Agouti-based (**b**) sets. The synteny-based sets comprise predictions from three different methods, ADseq, Gos-Asm, and OrthoStitch, as well as their liberal union (all non-conflicting predictions), their two-way consensus (2-way Cons. predicted by two methods and not conflicting with the third method), and their three-way consensus (3-way Cons. predicted by all three methods). Adjacencies that are exactly matching form the green base common to both sets in each comparison, from which extend bars showing physical mapping or Agouti adjacency counts (left) and synteny-based adjacency counts (right) that are unique (yellow) or conflicting (orange) in each comparison. Blue dashed lines highlight the total adjacencies for the physical mapping or Agouti sets. For comparison, all *y*-axes are fixed at a maximum of 350 adjacencies, except for *Anopheles atroparvus*. Results for two strains are shown for *Anopheles stephensi*, SDA-500 and Indian (I)

Combining the synteny-based results with physical mapping data from a subset of the anophelines allowed for enhanced superscaffolding as well as independent validations of the synteny-based predictions and their consensus sets. Building cytogenetic photomaps and conducting extensive FISH experiments mapped 31 *A. albimanus* scaffolds [27], 46 *A. atroparvus* scaffolds [25, 26, 58], 202 *A. funestus* scaffolds [25, 59–61] (including additional mapping for this study), 52 *A. sinensis* scaffolds (Chinese) [23], 99 *A. stephensi* (SDA-500) scaffolds [25], and 118 *A. stephensi* (Indian) scaffolds [21] (including additional mapping for this study) (see the “Methods” section; Additional file 1: Figure S11 and Tables S6, S7). The scaffold adjacencies identified from these physical mapping data, i.e. pairs of neighbouring mapped scaffolds, were compared with adjacencies predicted by each of the three methods and the Camsa-generated consensus sets (Additional file 1: Table S8). *A. funestus* validations confirmed 12–17% of the different sets of synteny-based adjacencies and highlighted conflicts with just 4–8%, while for *A. atroparvus*, 5 of the 15 two-way consensus synteny-based predictions were confirmed by physical mapping and only 1 conflict was identified (Fig. 4a). Examining the identified conflicts in detail revealed that most were resolvable. As not all scaffolds were targeted for physical mapping, neighbouring scaffolds on the physical maps could have shorter unmapped scaffolds between them that were identified by the synteny-based approaches. For *A. funestus*, five conflicts were resolved because the synteny-based neighbour was short and not used for physical mapping and an additional four conflicts were resolved by switching the orientation of physically mapped scaffolds, which were anchored by only a single FISH probe, and therefore, their orientations had not been confidently determined.

Transcriptome data from RNAseq experiments enabled further superscaffolding and validations of the synteny-based predictions and their consensus sets. The Annotated Genome Optimization Using Transcriptome Information (Agouti) tool [62] employs RNAseq data to identify adjacencies when individual transcripts (or paired-end reads) reliably map to scaffold extremities. Using available mapped paired-end RNAseq data from VectorBase [53, 54], Agouti predicted scaffold adjacencies for 13 anophelines (Additional file 1: Table S9). These Agouti-based scaffold adjacencies were compared with the adjacencies predicted by each of the three methods and the Camsa-generated consensus sets (Fig. 4b; Additional file 1: Table S10). Across all 13 assemblies, 18% of Agouti-based scaffold adjacencies supported the two-way consensus synteny-based adjacencies, 75% were unique to the Agouti sets, and only 7% were in conflict. The numerous adjacencies for *A. stephensi* (Indian) confirmed only eight of the two-way consensus set adjacencies, while about half as many adjacencies each for *A. stephensi* (SDA-500) and *A. funestus* confirmed four to five times as many two-way consensus set adjacencies with very few conflicts (Fig. 4b). Notably, most Agouti-based adjacencies that produced conflicts with the two-way consensus set adjacencies comprised scaffolds with no annotated orthologues. Such non-annotated scaffolds were also numerous amongst the adjacencies that were unique to Agouti. These cases can be resolved by noting that only scaffolds with orthologous genes were used for synteny-based predictions; therefore, the inferred neighbouring scaffolds could have shorter non-annotated scaffolds between them that were identified by Agouti.

### Superscaffold comparisons with new genome assemblies

**Fig. 5:**
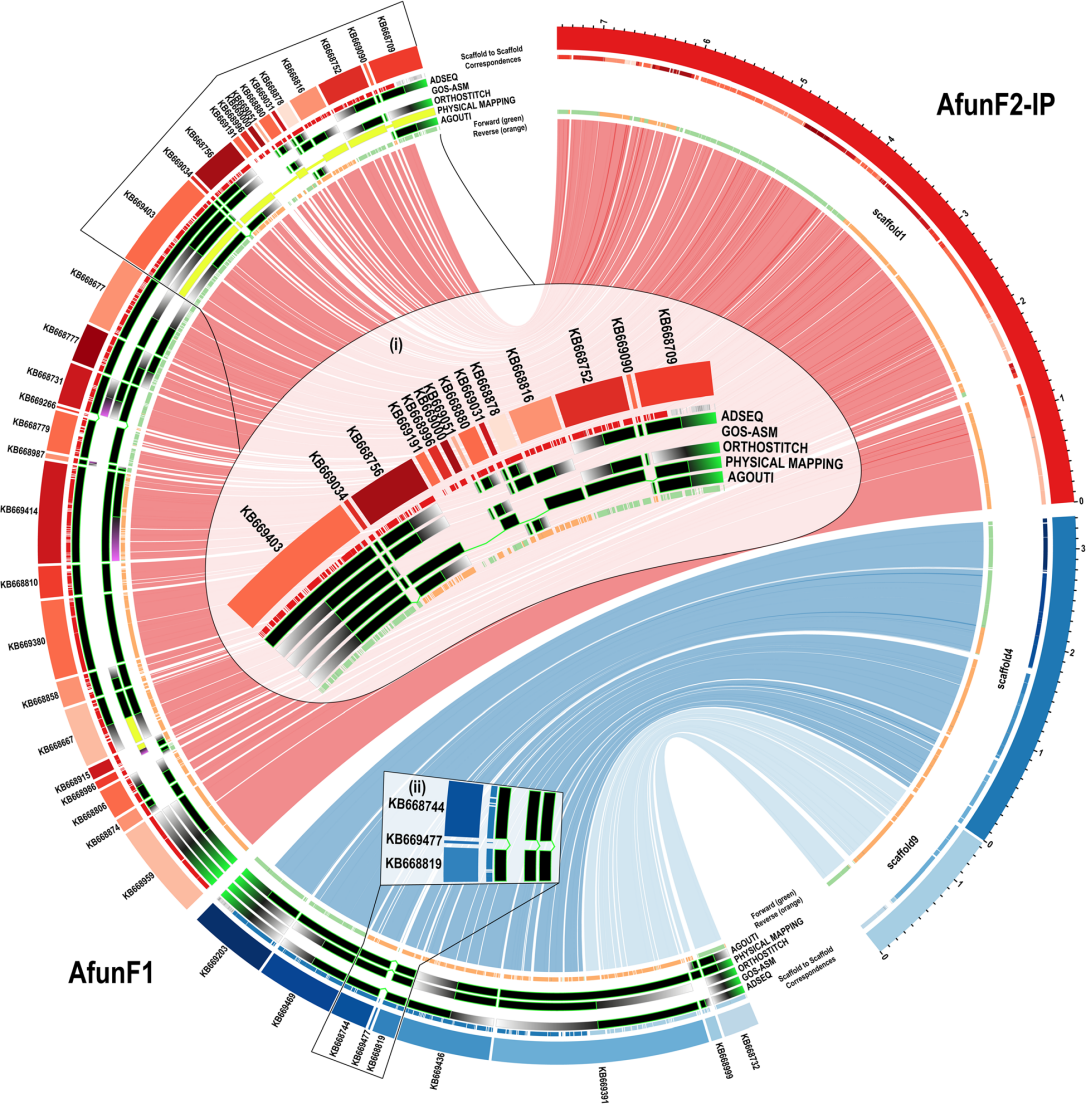
Whole genome alignment comparisons of selected *Anopheles funestus* AfunF1 and AfunF2-IP scaffolds. The plot shows correspondences of three AfunF2-IP scaffolds (right) with AfunF1 (left) scaffolds based on whole genome alignments, with links coloured according to their AfunF2-IP scaffold. Putative adjacencies between AfunF1 scaffolds are highlighted with tracks showing confirmed neighbours (black with bright green borders), supported neighbours with conflicting orientations (yellow), scaffolds with putative adjacencies that conflict with the alignments (purple gradient), scaffolds without putative adjacencies and thus no conflicts with the alignments (grey gradient) for: from outer to inner tracks, ADseq, Gos-Asm, OrthoStitch, physical mapping, and Agouti. The innermost track shows alignments in forward (green) and reverse (orange) orientations. The outermost track shows alignments coloured according to the corresponding scaffold in the other assemblies (light grey if aligned to scaffolds not shown). Inset (i) shows how corrected orientations of physically mapped scaffolds agree with the other methods. Inset (ii) shows how the alignments identified a short scaffold that was placed between two scaffolds identified by three other methods

A new *A. funestus* assembly, designated AfunF2-IP, was generated as part of this study by merging approximately 70× of PacBio sequencing data with the reference assembly (AfunF1), with subsequent scaffolding using the original Illumina sequencing data (see the “Methods” section; Additional file 1: Fig. S12 and Table S11). This AfunF2-IP assembly for *A. funestus* enabled the validation of the scaffold adjacency predictions for the AfunF1 assembly by examining collinearity between the two assemblies. AfunF1 scaffolds were ordered and oriented based on their alignments to AfunF2-IP scaffolds, and the resulting alignment-based scaffold adjacencies were then compared with the synteny-based and Agouti predictions as well as with the physical mapping adjacencies to identify supported, unique, and conflicting adjacencies (Fig. 5; Additional file 1: Figure S13 and Table S12). Each of the three synteny method prediction sets, as well as the two-way consensus and liberal union sets, had 14–17.5% in common with the alignment-based scaffold adjacencies, fewer than a quarter in conflict, and almost two thirds that were neither supported nor in conflict (Additional file 1: Table S12). The physical mapping adjacencies had generally more support, but also more conflicts as about half disagreed with the alignment-based adjacencies. Several disagreements were easily resolved by comparing these conflicts with those identified from the synteny-based adjacencies and confirming that switching the orientation of physically mapped scaffolds corrected the relative placements of these scaffolds, e.g. Fig. 5 inset (i). Similarly to the comparisons with the physical mapping and RNAseq data presented above, apparent conflicts with the alignment-based adjacencies can also arise because using genome alignment data considered all alignable scaffolds while physical mapping targeted only large scaffolds and synteny methods did not consider scaffolds with no annotated orthologues (i.e. short scaffolds). This is exemplified in Fig. 5 inset (ii) where the alignment data placed a short scaffold between two scaffolds predicted to be neighbours by ADseq, OrthoStitch, and physical mapping data. Skipping such short scaffolds (< 5 Kbp) to define a smaller set of alignment-based adjacencies considering only the longer scaffolds resulted in increased support for the synteny-based sets and most notably up to 39% for the physical mapping adjacencies, while only marginally increasing support for Agouti predictions (Additional file 1: Table S12). The availability of a new chromosome-level assembly built using long-reads and Hi-C data from the same *A. funestus* FUMOZ colony [63] allowed for additional validations of the scaffold adjacency predictions for the AfunF2 assembly. Comparing the AfunF1 and AfunF2 assemblies with the new AfunF3 assembly using the Quality Assessment Tool Quast-LG [64] identified 1980 and 2191 differences, respectively, with the majority in both comparisons being relocations, i.e. breakpoints on the same chromosome (Additional file 1: Table S13). Visualising collinearity with ‘dot plots’ built with D-Genies (Dot plot large Genomes in an Interactive, Efficient and Simple way) [65] showed overall good concordance and a high level of coverage, with 50 putative inversion and/or translocation events, three fifths of which were local inversions, i.e. correct placements but inverted orientations with respect to AfunF3 (Additional file 1: Figure S14).

Re-scaffolding of the initial *A. farauti* (AfarF1) and *A. merus* (AmerM1) assemblies employed large-insert ‘Fosill’ sequencing libraries and reduced the numbers of scaffolds and increased N50 values [25]. The availability of these re-scaffolded assemblies enabled the validation of the synteny-based and Agouti-based scaffold adjacency predictions for the AfarF1 and AmerM1 assemblies by examining corresponding scaffolds from the AfarF2 and AmerM2 assemblies (see the “Methods” section; Additional file 1: Figure S15). The comparisons identified full support for the majority of the two-way synteny consensus set adjacencies and few unresolvable conflicts, while the Agouti-based adjacencies achieved similarly high levels of full support but with slightly greater proportions of conflicts (Additional file 1: Table S14).

### Updated cytogenetic photomaps and physical genome maps for *A. funestus* and *A. stephensi*

**Fig. 6:**
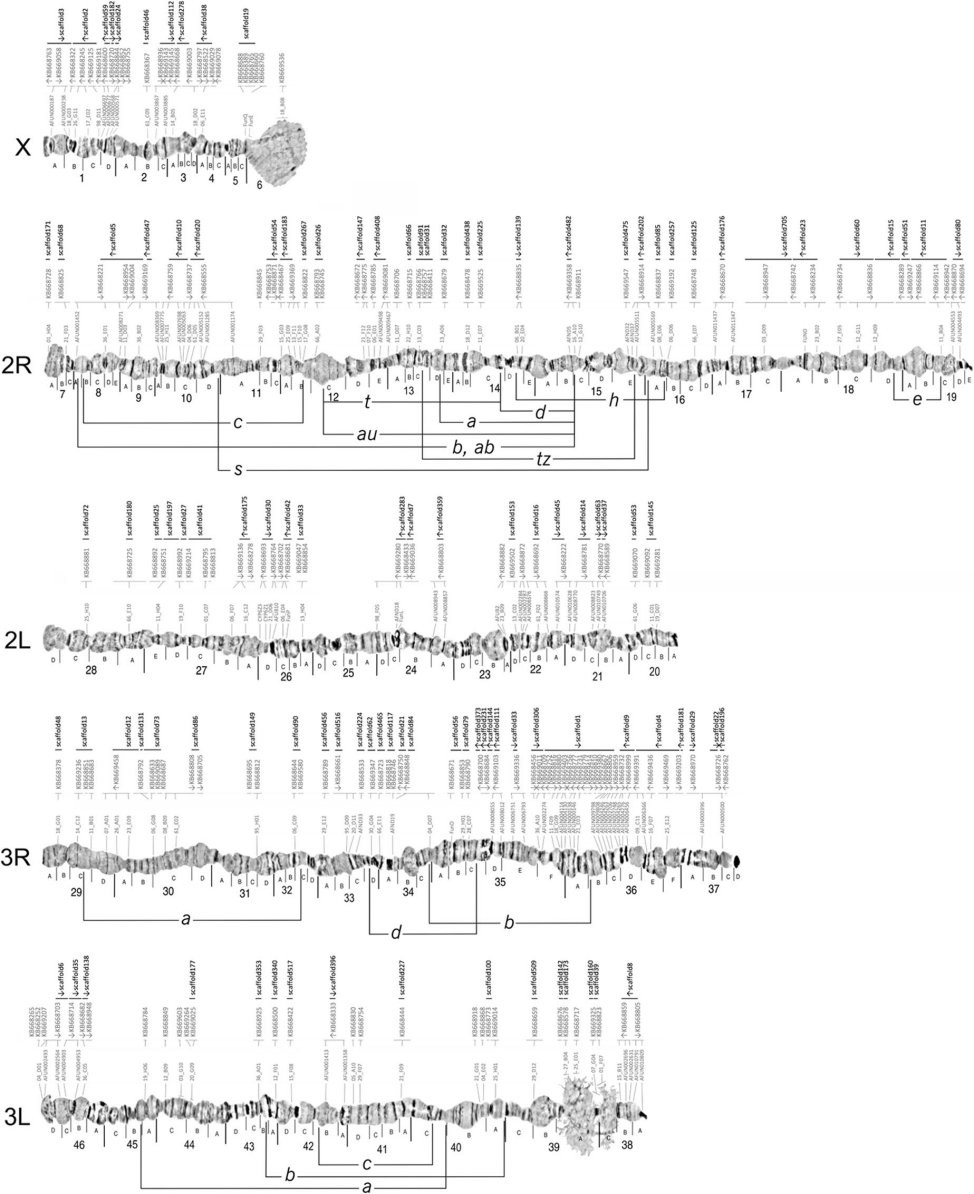
The *Anopheles funestus* cytogenetic photomap of polytene chromosomes with anchored scaffolds from the AfunF1 and AfunF2-IP assemblies. FISH-mapped DNA markers (grey probe identifiers directly above each chromosome) show the density of physical mapping along the chromosome arm subdivisions (labelled with letters A, B, C, etc. directly below each chromosome) and divisions (labelled with numbers 1–46 below the subdivision labels). Scaffolds from the AfunF1 (KB66XXXX identifiers, grey font and thin horizontal lines) and AfunF2-IP (scaffoldXX identifiers, black font and thick horizontal lines) assemblies are ordered along the photomap above each chromosome. Orientation of the scaffolds in the genome, if known, is shown by the arrows below each of the scaffold identifiers. Known polymorphic inversions are shown for chromosome arms 2R, 3R, and 3L

**Fig. 7:**
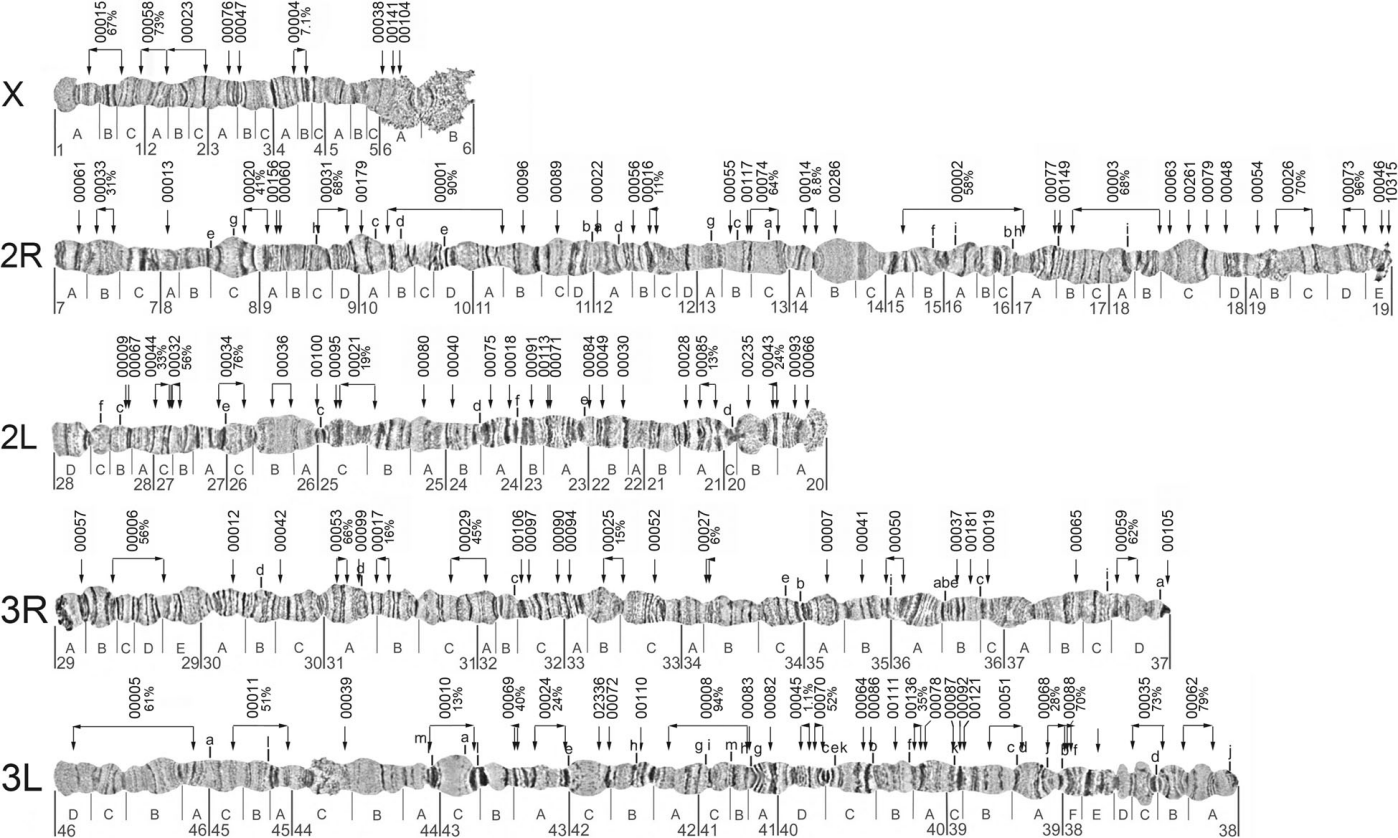
The *Anopheles stephensi* cytogenetic photomap of polytene chromosomes with anchored scaffolds from the AsteI2 assembly. The updated cytogenetic photomap is shown with chromosome arm subdivisions (labelled with letters A, B, C, etc. directly below each chromosome) and divisions (labelled with numbers 1–46 below the subdivision labels). Locations of known polymorphic inversions are indicated with lowercase letters above chromosome arms 2R, 2L, 3R, and 3L. The AsteI2 assembly identifiers of the 118 mapped scaffolds are shown above each chromosome arm (scaffold identifiers are abbreviated, e.g. ‘scaffold_00001’ is shown on the map as ‘00001’), and the locations of FISH probes used to map the scaffolds are shown with downward-pointing arrows. For scaffolds with two mapped FISH probes, the orientations along the genome map are shown with horizontal arrows below each of the scaffold identifiers, with labels indicating the proportion (%) of each scaffold located between the probe pairs

The collated data allowed for comprehensive updates of the previously published chromosomal photomaps from ovarian nurse cells for *A. funestus* [59] and for *A. stephensi* [66]. The existing images of *A. funestus* polytene chromosomes of the five arms common to all anophelines (X, 2R, 2L, 3R, and 3L) were further straightened to facilitate linear placements of the genomic scaffolds on the photomap (Fig. 6). Major structural updates to the *A. funestus* cytogenetic photomap included reversal of the order of divisions and subdivisions within the 3La inversion to follow the standard 3L+^a^ arrangement, and merging of two small subdivisions with larger neighbouring subdivisions: 5D to 6 and 34D to 34C. The previous physical genome map of the AfunF1 assembly included 104 scaffolds and spanned 35% of the assembly [25]. The extensive additional physical mapping performed for *A. funestus*, together with the new AfunF2-IP assembly and sequence alignment-based comparisons with the AfunF1 assembly, enabled an updated physical genome map to be built (Fig. 6). The 126 previously FISH-mapped [59–61] and 66 newly FISH-mapped DNA markers (Additional file 1: Figure S11) were located with BLAST searches to 139 AfunF1 scaffolds and then compared with AfunF2-IP scaffolds using whole genome pairwise alignments (see the “Methods” section). The placement of scaffolds along the photomap took advantage of comparisons with the synteny-based scaffold adjacency predictions and with the AfunF1-AfunF2-IP whole genome pairwise alignments. Synteny- or alignment-based scaffold neighbours were added to the genome map when they were short and thus had not been used for physical mapping. Additionally, scaffolds which were anchored with only a single FISH probe (i.e. with undetermined orientations) were reoriented when synteny- or alignment-based scaffold adjacencies provided supporting evidence to correct their relative placements on the map. The resulting physical genome map for *A. funestus* includes 202 AfunF1 scaffolds spanning 61% of the assembly (Additional file 1: Table S7), with a further 100 neighbouring scaffolds (additional 12% of the assembly) after incorporating the synteny-based and Agouti-based adjacencies. For *A. stephensi* (Indian), structural updates to the cytogenetic photomap [66] included changing the order of lettered subdivisions on arms 2L and 3L to match the order of numbered divisions (Fig. 7). The previous physical genome map of the AsteI2 assembly included 86 scaffolds and spanned 62% of the assembly [21]. The additional FISH probes allowed for 43 scaffolds to be oriented and placed a total of 118 scaffolds on the cytogenetic photomap spanning 79% of the assembly (Fig. 7) with a further 90 neighbouring scaffolds (additional 5% of the assembly) after incorporating all reconciled adjacencies.

## Discussion

Integrating synteny-based scaffold adjacency predictions with additional supporting data for subsets of the anophelines enabled superscaffolding with chromosome anchoring and arm assignments to produce 20 new *Anopheles* assemblies (Fig. 1; Tables 1 and 2). Consensus predictions were used to build the improved assemblies for which the general trend showed that a reduction in the total number of orthologue-bearing scaffolds of about a third could double the scaffold N50 (Fig. 2). Notably, when the scaffolds involved were long, even a handful of adjacencies could greatly increase N50s; however, the numerous adjacencies for the rather fragmented input assemblies improved their contiguity but led to only minor N50 improvements. For the six assemblies with input N50s of between 340 and 840 Kbp (considering all scaffolds, not only those with orthologues), the average improvement was just under 400 Kbp, demonstrating what can be achieved using only synteny-based approaches. By way of comparison, the honeybee genome assembly upgrade relied on millions of reads from ~ 20× SOLiD and ~ 5× Roche 454 sequencing to improve the scaffold N50 from 359 to 997 Kbp [67]. Thus, while the *Anopheles* results varied considerably depending on the input assemblies, using only gene synteny-based adjacencies from a combined analysis of the results of three methods achieved substantial contiguity improvements for many assemblies.

Results from comparing predicted adjacencies from the three synteny-based methods (Fig. 3) highlight the challenge of inferring accurate adjacencies as well as the importance of employing multiple approaches. Only 10% of all distinct scaffold adjacencies were predicted by all three methods, but building the two-way consensus sets increased this three-method agreement more than threefold, and almost all the two-way consensus adjacencies were supported by ADseq, nearly three quarters by OrthoStitch, and three fifths by Gos-Asm. Consensus building therefore takes advantage of differences amongst the employed methods to achieve the goal of identifying a subset of well-supported adjacencies. Synteny block delineation, which then allows for scaffold adjacencies to be predicted, is itself a complex task where results from different anchor-based approaches can vary considerably [68]. Several key differences distinguish the three methods applied to the *Anopheles* assemblies, for example, Gos-Asm employs only single-copy orthologues so any gene duplications are excluded from the ancestral genome reconstructions, whereas the other two methods do consider paralogues. Furthermore, both Gos-Asm and ADseq are ‘phylogeny-aware’ algorithms as they use the species tree topology, and ADseq additionally employs individual gene trees for each orthologous group. In contrast, OrthoStitch does not take phylogenies into account and instead relies on enumerating levels of support across the dataset to score putative adjacencies. These differences affect the sensitivity and specificity of the methods, reflected by the more numerous predictions from ADseq that can explore complex gene evolutionary histories within the species tree topology, versus the smaller sets of adjacencies from Gos-Asm, which excludes complexities introduced by gene duplications, and OrthoStitch that simplifies the search by not imposing any evolutionary model. Thus, while applying a consensus approach to filter adjacency predictions results in reduced sensitivities, it takes advantage of the different underlying assumptions and algorithmic implementations of each method to identify common sets of well-supported scaffold adjacencies to enable confident superscaffolding.

The input data are another factor that may influence the number of predicted adjacencies, the level of agreement amongst different methods, and the achievable contiguity improvements. An assembly with many short scaffolds with annotated orthologues may achieve numerous adjacency predictions, e.g. *A. maculatus*, but an assembly with such low contiguity is less likely to provide support for putative adjacencies in other assemblies. The evolutionary divergence of the set of species, as well as the total number of species, to which these methods are applied would also impact their ability to recover reliable adjacencies, because the complexity of the task of inferring synteny blocks is greatly reduced if the input orthology dataset consists mainly of near-universal single-copy orthologues. As gene duplications and losses accumulate over time, the proportion of near-universal single-copy orthologues will shrink, and even amongst those that are maintained, translocations and genomic shuffling events will add to the steady erosion of the evolutionary signals on which these methods rely. Rearrangements may also be more or less common in different genomic contexts, e.g. the *Osiris* [69] and *TipE* [70] gene clusters have been noted for their unusually high synteny conservation across insects, or in different species, e.g. the well-known *Hox* gene cluster is largely collinear across animals but may be found with disorganised, split, or atomised arrangements [71]. Genomic shuffling rates may also vary amongst different lineages—e.g. lepidopteran genomes appear to have reduced levels of gene rearrangements [72]—so seemingly equally divergent (in terms of time to last common ancestor) sets of species may be differentially amenable to superscaffolding through synteny delineation.

Comparisons of the predictions based solely on synteny inferences with alternative scaffold adjacency datasets demonstrated their complementarity and the benefits of integrating different data types. Although generally few adjacencies were obtained from the physical mapping data, the comparisons were able to identify support for many synteny-based adjacencies (Fig. 4a). Several conflicts were also identified; however, most of these were due to the fact that the synteny-based neighbour was a short scaffold that had not been targeted for physical mapping and could be positioned between the two much larger physically mapped scaffolds; thus, they are not truly conflicts. Importantly, other conflicts involved only the relative orientation of neighbouring scaffolds and occurred with scaffolds that were anchored with only a single FISH probe and whose orientations had thus not been confidently determined. In these cases, the synteny-based adjacencies therefore provided key complementary information and helped to correct the orientations of the physically mapped scaffolds. Comparisons with RNAseq-based adjacencies also provided support for many synteny-based predictions (Fig. 4b). Two thirds of the adjacencies unique to the RNAseq predictions were between scaffolds where one or both had no annotated orthologues. As Agouti is not restricted to large scaffolds preferred for physical mapping or scaffolds with annotated orthologues required for synteny-based approaches, it can provide complementary predictions that capture shorter non-annotated scaffolds that would otherwise not be recovered. While this would not substantially improve N50 values, it is nonetheless important for improving gene annotations as correcting such assembly breaks could allow for more complete gene models to be correctly identified.

The *A. funestus* PacBio-based AfunF2-IP assembly scaffolds facilitated the alignment-based ordering and orientation of AfunF1 scaffolds for comparisons with the adjacency predictions and physical mapping data (Fig. 5). These supported up to almost a quarter of *A. funestus* two-way consensus synteny adjacencies and about 40% of the physical mapping adjacencies. Importantly, most were neither supported nor in conflict, and conflicts generally occurred when the alignment-based adjacencies included short scaffolds that were not considered by the synteny-based or physical mapping approaches and thus could be resolved. Comparisons with the AfunF3 chromosome-level assembly showed generally very good agreement and highlighted few large-scale differences, i.e. a small number of rearrangements most likely due to erroneous superscaffolding. Instead, most differences were small-scale and local, i.e. rearrangements most likely resulting from small inversion errors, which Hi-C methods are prone to due to noise in the data [73]. For *A. farauti* and *A. merus*, the genome alignment-based comparisons of their initial assemblies with the re-scaffolded AfarF2 and AmerM2 assemblies provided much higher levels of support for the two-way consensus synteny adjacencies, with very few conflicts. This reflects the radically different approaches between re-scaffolding, where the additional ‘Fosill’ library data served to build longer scaffolds from the initial scaffolds, versus the Illumina-PacBio hybrid re-assembly of *A. funestus*. These comparisons therefore validate many of the synteny-based adjacency predictions while conceding that short intervening scaffolds may be overlooked due to the limitations of having to rely on scaffolds with annotated orthologues.

As modern long-read and long-range sequencing technologies are capable of producing highly contiguous assemblies [74], it is conceivable that many fragmented draft genomes will be completely superseded by new independently built high-quality reference assemblies. For example, single-molecule sequencing technologies were recently employed to produce assemblies of 15 *Drosophila* species, 14 of which already had previously reported sequenced genomes [75]. Re-sequencing to obtain proximity data to use in conjunction with contigs from draft assemblies can also achieve high-quality references to replace the fragmented initial versions, e.g. [9, 76]. Such new protocols and technologies have been successfully applied to build an assembly (372 scaffolds) for the Ngousso strain of *A. coluzzii* [77] and a new chromosome-scale assembly for *A. funestus* (1053 scaffolds) [63]. Alternatively, although reference-assisted assembly approaches may mask true genomic rearrangements [68], high-quality chromosome-level genomes of very close relatives can be used to improve draft assemblies, often employing alignment-based comparisons such as assisted assembly tools [78], reference-assisted chromosome assembly [79], Chromosomer [80], the Reference-based Genome Assembly and Annotation Tool [81], or the Ragout 2 reference-assisted assembly tool [82]. What role then is there for comparative genomics approaches that use evolutionary signals to predict scaffold adjacencies in draft assemblies?

Firstly, while recognising that downward trending costs of many new technologies are making sequencing-based approaches more accessible to even the smallest of research communities, the costs and time associated with experimental finishing or re-sequencing efforts remain non-trivial and acquired expertise is needed for high-quality sample preparation and library building. Furthermore, the disappointing reality is that re-sequencing and re-scaffolding does not always lead to vastly improved assemblies, albeit an anecdotal reality because failures are not reported in the published literature. Secondly, hybrid assembly approaches benefit from the complementarity of the different types of input data that they employ, and our comparisons show that synteny-based adjacencies can further complement the experimental data. In this regard, even if synteny-based results are not directly included in such hybrid approaches, they can nevertheless serve as a benchmark against which to quantify the effectiveness of different combinations of approaches (or different parameters used) and help guide re-assembly procedures towards producing the best possible improved assemblies. Thirdly, reference-assisted assembly approaches work best with good quality closely related reference and outgroup genomes, which are not always available. The anophelines analysed here shared a common ancestor some 100 million years ago, and only about 9% of the *A. gambiae* (PEST) genome was alignable to the most distant relatives [25]. Previous comparisons of *Ae. aegypti* and *A. gambiae* revealed that almost 80% of their single-copy orthologues were retained in the same genomic neighbourhood [83], and using protein sequence alignments identifies recognisable orthologues for about 80% of genes between the most distant pairs of anophelines. Multi-species gene synteny-based approaches are therefore well-suited to the analysis of datasets such as the 21 *Anopheles* assemblies.

Finally, our results show how physical mapping datasets can be augmented or even corrected through comparisons with synteny-based scaffold adjacency predictions. Where subsets of scaffolds have already been mapped to chromosomes (Figs. 6 and 7; Table 2), adding neighbouring scaffolds from synteny-based predictions can add to the overall total proportion anchored without more labour-intensive experimental work. Superscaffolding also reduces the total numbers of scaffolds to be mapped and thus allows for greater proportions of draft assemblies to be anchored using fewer markers. Comprehensive anchoring in multiple species in turn allows for greater confidence from cross-species comparisons to assign non-anchored scaffolds to chromosome arms. These new anopheline assemblies with enhanced chromosome mapping represent greatly improved genomic resources for a wide range of future studies. For example, chromosome anchoring and arm assignments have facilitated investigations such as rates of gene translocations between chromosome arms [25], genetics of saltwater tolerance [84] or resting behaviour and host preference [85], chromosome arm-specific patterns of polymorphism [86], sex-biased gene expression [87], dosage compensation [88], or evolution of sex chromosomes [89, 90].

## Conclusions

Our three-method consensus synteny-based scaffold adjacency prediction workflow is relatively easily implemented and may flexibly include results from additional adjacency predictors. Alternative sources of adjacency information may also be incorporated as evidenced with our various types of comparison datasets. Rather than prescribing a panacea to cure all assembly ailments, we conclude that the components of this workflow may be adapted, substituted, extended, or simplified according to the needs and resources of draft genome assembly improvement projects. Evaluating the performance of three comparative genomics approaches and comparing their results with available experimental data demonstrate their utility as part of assembly improvement initiatives, as well as highlighting their complementarity to experimental approaches. Although resulting improvements may vary depending on the contiguity of the input assemblies, the consensus predicted scaffold adjacencies can lead to substantial improvements of draft assemblies without requiring additional sequencing-based support. They can also add to and improve physical mapping efforts and chromosome arm assignments. These evolutionarily guided methods therefore augment the capabilities of any genome assembly toolbox with approaches to assembly improvements or validations that will help to propel the draft assemblies from similar species clusters along the journey towards becoming ‘finished’ reference genomes.

## Methods

### Synteny-based scaffold adjacency predictions

The synteny-based prediction tools require as input both delineated orthology and genomic location data for the annotated genes from each assembly. All gene annotations were retrieved from VectorBase [53, 54], and orthology data were retrieved from OrthoDB v9 [91]: versions of the genome assemblies and their annotated gene sets are detailed in Additional file 1: Table S3, along with counts of scaffolds, genes, and orthologues. With an average of 11,832 orthologues (standard deviation 1075), including 10,708 orthologous groups with genes from more than half of the 21 anophelines, these data provide a comprehensive set of genomic markers for gene synteny-based approaches. The complete ‘frozen’ input datasets of orthology relationships and genomic locations of the annotated genes for each of the 21 assemblies are presented in Additional file 3. ADseq analysis first builds reconciled gene trees for each orthologous group (gene family); then for pairs of gene families for which extant genomic adjacencies are observed, or suggested by sequencing data, a duplication-aware parsimonious evolutionary scenario is computed, via Dynamic Programming (DP), that also predicts extant adjacencies between genes at the extremities of contigs or scaffolds. This DP algorithm also accounts for scaffolding scores obtained from paired-end reads mapped onto contigs and provides a probabilistic score for each predicted extant adjacency, based on sampling optimal solutions [55]. ADseq was applied across the full anopheline input dataset to predict scaffold adjacencies (Additional file 1: Table S4). Gos-Asm (gene order scaffold assembler) employs an evolutionary rearrangement analysis strategy on multiple genomes utilising the topology of the species phylogenetic tree and the concept of the breakpoint graph [56]. Fragmented genomes with missing assembly ‘links’ between assembled regions are modelled as resulting from artificial ‘fissions’ caused by technological fragmentation that breaks longer contiguous genomic regions (chromosomes) into scaffolds [32]. Assembling these scaffolds is therefore reduced to a search for technological ‘fusions’ that revert non-evolutionary ‘fissions’ and glue scaffolds back into chromosomes. Gos-Asm was applied to the full anopheline input dataset to predict such scaffold ‘fusions’ (Additional file 1: Table S4). The OrthoStitch approach was first prototyped as part of the investigation of greater synteny conservation in lepidopteran genomes [72], and subsequently further developed as part of this study to include a scoring system and additional consistency checks. Searches are performed to identify orthologues (both single-copy and multi-copy orthologues are considered) at scaffold extremities in a given assembly that form neighbouring pairs in the other compared assemblies, thereby supporting the hypothesis that these scaffolds should themselves be neighbours. OrthoStitch was applied to the full anopheline input dataset to predict scaffold adjacencies (Additional file 1: Figures S5, S6 and Table S4). Further details of the assumptions, implemented algorithms, and tested performance of these three approaches are presented in Additional file 1. The Camsa tool [57] was used to compare and merge scaffold assemblies produced by the three methods by identifying adjacencies in three-way and two-way agreement (with no third-method conflict) (Additional file 1: Table S5). Camsa was also used to build merged assemblies using only conservative three-way consensus adjacencies and using liberal unions of all non-conflicting adjacencies. Quantifications of assembly improvements considered only scaffolds with annotated orthologous genes (because the synteny-based methods rely on orthology data) to count the numbers of scaffolds and compute scaffold N50s before and after merging (Fig. 2; Additional file 1: Figures S7, S8). The results of the Camsa merging procedure were used to quantify all agreements and conflicts amongst the different sets of predicted adjacencies (Fig. 3; Additional file 1: Figures S9, S10 and Table S5). A Docker container is provided that packages ADseq, Gos-Asm, OrthoStitch, and Camsa, as well as their dependencies, in a virtual environment that can run on a Linux server. See Additional file 1 for further details for all synteny-based predictions and their comparisons, and the Docker container.

### Integration of physical mapping and RNA sequencing data

Methods for chromosomal mapping of scaffolds [92, 93] are detailed for *A. albimanus* [27], *A. atroparvus* [25, 26, 58], *A. stephensi* (SDA-500) [25], *A. stephensi* (Indian) [21], and *A. sinensis* (Chinese) [23]. *A. funestus* mapping built on previous results [59–61] with additional FISH mapping (Additional file 1: Figure S11) used to further develop the physical map by considering several different types of mapping results. *A. stephensi* mapping also extended previous efforts [94] by aligning FISH probes to the AsteI2 scaffolds with BLAST, and designing and hybridising new probes targeting specific scaffolds to increase the coverage. The complete ‘frozen’ input datasets of the physically mapped scaffolds for each of the six assemblies are presented in Additional file 4, with the usable scaffold pair adjacencies in Additional file 1: Table S6, the definitive mapped *A. funestus* scaffolds in Additional file 1: Table S7, and the definitive chromosome-mapped scaffolds for each of the six assemblies as well as for *A. arabiensis* in Additional file 5. These adjacencies were compared with the Camsa-generated two-way consensus assemblies, as well as the predictions from each method and the conservative and liberal consensus assemblies (Fig. 4a; Additional file 1: Table S8). RNAseq-based scaffolding has been employed for very large genomes such as the Norway spruce [95] and the Loblolly pine [96], but is also applicable to smaller genomes where more compact gene structures would make it less likely to erroneously skip intervening intronic scaffolds/contigs. The RNAseq-based adjacency predictions used genome-mapped paired-end sequencing data for 13 of the anophelines available from VectorBase [53, 54] (Release VB-2017-02), including those from the *Anopheles* 16 Genomes Project [25] and an *A. stephensi* (Indian) male/female study [97]. Agouti [62] analyses were performed (requiring unique read mapping and a minimum coverage of 5 reads) to identify transcript-supported scaffold adjacencies for these 13 anophelines, complemented with Rascaf [98] predictions (Additional file 1: Table S9). These adjacencies were compared with the Camsa-generated two-way consensus assemblies, as well as the predictions from each method and the conservative and liberal consensus assemblies (Fig. 4b; Additional file 1: Table S10). See Additional file 1 for further details for physical mapping and Agouti adjacencies and their comparisons.

### Building the new assemblies

The new assemblies were built using the different datasets available for each of the anophelines (Additional file 1: Figure S1): synteny data only for six, *A. christyi*, *A. coluzzii*, *A. culicifacies*, *A. darlingi*, *A. maculatus*, and *A. melas*; synteny and Agouti data for eight, *A. arabiensis*, *A. dirus*, *A. epiroticus*, *A. farauti*, *A. merus*, *A. minimus*, *A. quadriannulatus*, and *A. sinsensis* (SINENSIS); synteny and physical mapping data for *A. sinensis* (Chinese); synteny, Agouti, and physical mapping data for four, *A. albimanus*, *A. atroparvus*, *A. stephensi* (SDA-500), and *A. stephensi* (Indian); and synteny, Agouti, physical mapping data, and the new PacBio-based assembly for *A. funestus*. The new *A. arabiensis* assembly additionally incorporated scaffold orders determined by alignments to the *A. gambiae* (PEST) X chromosome from [51] and to autosomes provided by Xiaofang Jiang and Brantley Hall. The new *A. funestus* assembly generated as part of this study was based on approximately 70× of PacBio sequencing data polished with Quiver (from PacBio’s SMRT Analysis software suite). This was combined with the reference assembly (AfunF1) using Metassembler [99] to generate a merged assembly, and this merged assembly was then scaffolded with Sspace [100] using the original Illumina sequencing data, and designated the *A. funestus* AfunF2-IP assembly. The AfunF2-IP assembly improves on the reference AfunF1 assembly at contig level but not at scaffold level (Additional file 1: Figure S12 and Table S11). Where AfunF2-IP scaffolds span the ends of AfunF1 scaffolds, they provide support for AfunF1 scaffold adjacencies. Thus, whole genome alignments of the two assemblies were performed using Lastz [101] and used to identify corresponding genomic regions that enabled the alignment-based ordering and orientation of AfunF1 scaffolds, which were then compared with the synteny-based, physical mapping-based, and Agouti-based adjacencies (Fig. 5, Additional file 1: Figure S13 and Table S12). Using the AfunF1 assembly as the basis, and incorporating evidence from the AfunF2-IP assembly through scaffold correspondences established from the whole genome alignments, the physical mapping data and the synteny-based and Agouti-based adjacency predictions were integrated to build the new AfunF2 reference assembly for *A. funestus*. The AfunF1 and AfunF2 assemblies were then compared to the new chromosome-scale AfunF3 assembly [63] using the Quality Assessment Tool for large genomes Quast-LG [64] and ‘dot plots’ built with D-Genies (Dot plot large Genomes in an Interactive, Efficient and Simple way) [65] (Additional file 1: Figure S14 and Table S13). The comprehensive update to the photomap employed BLAST searches to identify positions of the physically mapped DNA markers within the AfunF1 and AfunF2-IP assemblies, and whole genome pairwise alignments to reconcile these two assemblies with the new photomap. Whole genome alignments of versions 1 and 2 assemblies for *A. farauti* and *A. merus* were used to delineate corresponding scaffolds and identify supported, unsupported, and conflicting adjacencies (Additional file 1: Figure S15 and Table S14). Reconciling all adjacencies produced the resolved sets of scaffold adjacencies and superscaffolds (Additional file 6) that were used to build all the new assemblies and the definitive chromosome anchoring data for seven assemblies (Additional file 7). The input assemblies, superscaffolded assemblies, and chromosome-level assemblies (where available) were assessed for completeness in terms of expected gene content using the Benchmarking Universal Single-Copy Orthologue assessment tool [102] (Additional file 1: Table S1). These updated assemblies, their correspondingly updated gene annotations, the orthology data used as input for the gene synteny-based approaches, and the definitive anchoring data were employed to assign non-anchored scaffolds to chromosome arms (Additional file 1: Table S15; Additional file 2). See Additional file 1 for further details on the workflow to integrate different adjacency predictions and build the new assemblies, the PacBio assembly generation, the genome alignment based comparisons of the AfunF1 and AfunF2-IP assemblies, the lift-over of gene annotations to the new assemblies, and the assignment of non-anchored scaffolds and superscaffolds to chromosome arms.

## Supplementary information

**Additional file 1.** Supplementary online material

**Additional file 2.** Chromosome arm assignments. Lists of scaffold assignments to chromosome arms.

**Additional file 3.** Orthology anchor data. Input data for the synteny analyses of orthology relationships and genomic locations of annotated genes.

**Additional file 4.** Physical mapping data. Input datasets of the physically mapped scaffolds.

**Additional file 5.** Chromosome mapping data. Reconciled chromosome-mapped scaffolds.

**Additional file 6.** Adjacency and superscaffold data. Reconciled sets of scaffold adjacencies and corresponding superscaffolds.

**Additional file 7.** Anchored scaffolds and superscaffolds. Definitive chromosome anchoring data.

## Data Availability

The updated assemblies of 20 anophelines and their updated gene annotations, as well as the corresponding chromosome maps of all anchored scaffolds and superscaffolds, are available from VectorBase [53, 54]. Assembly and annotation versions are detailed in Additional file 1, along with software versions and parameters employed for the analyses. Full results of scaffold assignments to chromosome arms are presented in Additional file 2. The input data for the synteny analyses of orthology relationships and genomic locations of the annotated genes are presented in Additional file 3. The complete input datasets of the physically mapped scaffolds for each of the six assemblies are presented in Additional file 4. The reconciled sets of chromosome-mapped scaffolds for seven assemblies are presented in Additional file 5. The reconciled sets of scaffold adjacencies and superscaffolds for all assemblies are presented in Additional file 6. The definitive chromosome anchoring data for seven assemblies are presented in Additional file 7.

## Abbreviations

AD: ADseq
AGO: Agouti-based
Agouti: Annotated Genome Optimization Using Transcriptome Information tool
ALN: Alignment-based
Camsa: Comparative Analysis and Merging of Scaffold Assemblies tool
DP: Dynamic programming
FISH: Fluorescence in situ hybridization
GA: Gos-Asm
Gos-Asm: Gene order scaffold assembler
Kbp: Kilobase pairs
Mbp: Megabase pairs
OS: OrthoStitch
PacBio: Pacific Biosciences
PB: PacBio-based
PHY: Physical mapping-based
QTL: Quantitative trait loci
RNAseq: RNA sequencing
SYN: Synteny-based
