# Supplementary material for: Evolutionary superscaffolding and chromosome anchoring to improve Anopheles genome assemblies
Source: BMC Biol. 2020 Jan 2;18:1. doi: 10.1186/s12915-019-0728-3 (PMC6939337; doi:10.1186/s12915-019-0728-3)
Supplement: Supplementary file 1 — Additional file 1. Supplementary online material [file 12915_2019_728_MOESM1_ESM.pdf]

SUPPLEMENTARY ONLINE MATERIAL

Evolutionary superscaffolding and chromosome anchoring to improve *Anopheles* genome assemblies

Robert M. Waterhouse, Sergey Aganezov, Yoann Anselmetti, Jiyoung Lee, Livio Ruzzante, Maarten J.M.F. Reijnders, Romain Feron, Sèverine Bérard, Phillip George, Matthew W. Hahn, Paul I. Howell, Maryam Kamali, Sergey Koren, Daniel Lawson, Gareth Maslen, Ashley Peery, Adam M. Phillippy, Maria V. Sharakhova, Eric Tannier, Maria F. Unger, Simo V. Zhang, Max A. Alekseyev, Nora J. Besansky, Cedric Chauve, Scott J. Emrich, Igor V. Sharakhov

Author emails & ORCIDs

Name	Email	ORCID
Robert M. Waterhouse	robert.waterhouse@unil.ch	0000-0003-4199-9052
Sergey Aganezov	aganezov@cs.jhu.edu	0000-0003-2458-8323
Yoann Anselmetti	yoann.anselmetti@gmail.com	0000-0002-6689-1163
Jiyoung Lee	jylee43@vt.edu	0000-0003-1702-874X
Livio Ruzzante	livio.ruzzante@unil.ch	0000-0002-8693-8678
Maarten J.M.F. Reijnders	maarten.reijnders@unil.ch	0000-0002-5657-4762
Romain Feron	romain.feron@unil.ch	0000-0001-5893-6184
Sèverine Bérard	Severine.Berard@umontpellier.fr	0000-0002-3029-0964
Phillip George	phippl3@vt.edu	NA
Matthew W. Hahn	mwh@indiana.edu	0000-0002-5731-8808
Paul I. Howell	paulihowell@gmail.com	0000-0002-3834-4621
Maryam Kamali	Kamali@modares.ac.ir	0000-0001-9955-0683
Sergey Koren	sergey.koren@nih.gov	0000-0002-1472-8962
Daniel Lawson	daniel.lawson@imperial.ac.uk	0000-0001-7765-983X
Gareth Maslen	gmaslen@ebi.ac.uk	0000-0001-7318-3678
Ashley Peery	peerya2@vt.edu	NA
Adam M. Phillippy	adam.phillippy@nih.gov	0000-0003-2983-8934
Maria V. Sharakhova	msharakh@vt.edu	0000-0002-5790-3548
Eric Tannier	eric.tannier@inria.fr	0000-0002-3681-7536
Maria F. Unger	munger1@nd.edu	NA
Simo V. Zhang	svm.zhang@gmail.com	0000-0003-2154-2549
Max A. Alekseyev	maxal@gwu.edu	0000-0002-5140-8095
Nora J. Besansky	nbesansk@nd.edu	0000-0003-0646-0721
Cedric Chauve	cedric.chauve@sfu.ca	0000-0001-9837-1878
Scott J. Emrich	semrich@utk.edu	0000-0002-4804-7436
Igor V. Sharakhov	igor@vt.edu	0000-0003-0752-3747

Contents

[1] Data analysis and adjacency reconciliation workflow overview ... 3
Figure S1. Workflows applied to upgrade the 20 anopheline assemblies ... 4
[2] Superscaffolding and chromosome arm assignments ... 5
Table S1. Assessments of assemblies with Benchmarking Universal Single-Copy Orthologues ... 5
Table S2. Assignment of scaffolds and superscaffolds to chromosome arms ... 6
Figure S2. Superscaffolding genomic spans of 20 anopheline genome assemblies ... 7
Figure S3. Increases in pairs and trios of syntenic orthologues after superscaffolding ... 8
Figure S4. Structural variants between *Anopheles gambiae* and *Anopheles arabiensis* ... 9
[3] Sources of input data for predicting adjacencies ... 10
Table S3. Assembly and orthology input data ... 10
[4] ADSEQ: scaffolding genomes using gene trees, synteny and sequencing data ... 11
[5] GOS-ASM: multi-genome rearrangement-based gene order scaffolder ... 13
[6] ORTHOSTITCH: scaffold adjacencies from conserved orthologous neighbours ... 14
Figure S5. Example of ORTHOSTITCH adjacency evidence ... 15
Figure S6. Performance of ORTHOSTITCH adjacency recovery ... 17
Table S4. Synteny-based adjacency predictions ... 18
[7] CAMSA: comparative analysis and merging of scaffold assemblies ... 18
Table S5. Synteny-based adjacency agreements ... 19
Figure S7. Assembly improvements based on conservative set synteny predictions ... 20
Figure S8. Assembly improvements based on liberal union set synteny predictions ... 21
Figure S9. Comparisons of adjacency results from three synteny-based methods ... 23
Figure S10. Proportions of synteny-based adjacencies in agreement for each assembly ... 24
[8] Physical mapping data from six anophelines ... 25
Table S6. Physically mapped scaffolds from six anophelines ... 25
Figure S11. Fluorescence *in situ* hybridization (FISH) mapping in *Anopheles funestus*. ... 27
Table S7. Physically mapped *Anopheles funestus* scaffolds on the cytogenetic map ... 28
Table S8. Physical mapping and synteny-based adjacency comparisons ... 33
[9] RNA sequencing data from 13 anophelines ... 34
Table S9. AGOUTI-based scaffold adjacencies from 13 anophelines ... 35
Table S10. AGOUTI and synteny-based adjacency comparisons ... 36
[10] Building the PacBio-based *Anopheles funestus* assembly ... 38
Table S11. Comparisons of *Anopheles funestus* assemblies ... 39
Figure S12. Cumulative scaffold lengths for *Anopheles funestus* AfunF1 and AfunF2-IP assemblies ... 39
[11] Examining collinearity between *Anopheles funestus* assemblies ... 40
Table S12. Alignment-based adjacency comparisons for *Anopheles funestus* ... 41
Figure S13. Collinearity between *Anopheles funestus* AfunF1 and AfunF2-IP scaffolds ... 42
Table S13. QUAST comparisons for *Anopheles funestus* ... 43
Figure S14A. Dot plot of *Anopheles funestus* AfunF2 scaffolds and AfunF3 chromosomes ... 46
Figure S14B. Dot plot of *Anopheles funestus* AfunF2 scaffolds and AfunF3 chromosome X ... 47
Figure S14C. Dot plot of *Anopheles funestus* AfunF2 scaffolds and AfunF3 chromosome 2 ... 48
Figure S14D. Dot plot of *Anopheles funestus* AfunF2 scaffolds and AfunF3 chromosome 3 ... 49
[12] Reconciliation to build the new assemblies ... 50
Table S14. Version 2 assembly reconciliations for *Anopheles farauti* and *Anopheles merus* ... 51
Figure S15. Collinearity between *Anopheles farauti* AfarF1 and AfarF2 scaffolds ... 52
Table S15. Chromosome arm to element correspondences in anophelines ... 54
[13] Software and database availability ... 54
[14] Main text figure credits ... 55
Supplementary References ... 56

[1] Data analysis and adjacency reconciliation workflow overview

Robert M. Waterhouse

Details of all steps are presented in the following sections, here we provide an overview of the production of different sets of scaffold adjacencies for each of the anophelines and the different workflows that were followed to reconcile all the data to build the new assemblies (**Figure S1**). The simplest workflow (**A**, six assemblies) was used for *A. christyi*, *A. coluzzii*, *A. culicifacies*, *A. darlingi*, *A. maculatus*, and *A. melas*, for which only consensus synteny predictions were produced. Workflow **B** (eight assemblies) reconciled the synteny-based two-way consensus sets with the adjacency predictions from RNA sequencing (RNAseq) data using the AGOUTI (Zhang et al. 2016) and RASCAF (Song et al. 2016) tools to build new assemblies for *A. arabiensis*, *A. dirus*, *A. epiroticus*, *A. farauti*, *A. merus*, *A. minimus*, *A. quadriannulatus*, and *A. sinsensis* (SINENSIS). Workflow **C** (four assemblies) additionally incorporated reconciliations with the available physical mapping data for *A. albimanus*, *A. atroparvus*, *A. stephensi* (SDA-500), and *A. stephensi* (Indian). Workflow **D** was applied to *A. funestus* to also incorporate reconciliations with the adjacencies produced from comparing the reference assembly (AfunF1) with the new Pacific Biosciences (PacBio) assembly (AfunF2-IP). And finally, workflow **E** was adopted for *A. sinensis* (Chinese) that employed just the synteny-based two-way consensus set and the physical mapping data. Finally, chromosome mapping data from *A. arabiensis* were combined with the workflow B results to produce the new chromosome-anchored assembly.

We employed gene orthology data delineated using ORTHODB (Zdobnov et al. 2017), but alternative methodologies may be used to define orthologous relations amongst the annotated gene sets of the species to be analysed. With gene orthology data and genomic location data from VECTORBASE (Giraldo-Calderón et al. 2015) prepared, we performed adjacency predictions with GOS-ASM (Aganezov and Alekseyev 2016) and ORTHOSTITCH (this study) directly, while ADSEQ (Anselmetti et al. 2015, 2018) first required building sequence alignments and reconciled trees before scaffold neighbours were predicted (see the following sections for details). We then employed the CAMSA tool (Aganezov and Alekseyev 2017) for comparative analyses of the results from our different scaffold adjacency predictions to automatically build the most confident merged-scaffold assembly, and we used CAMSA's interactive visualisation framework to inspect conflicts in the assembly graph. For the species with no validation datasets we employed a simple two-way consensus approach with no third-method conflicts to define the final adjacencies. For the other species, all conflicts identified between the two-way consensus adjacencies and the alternative sources of adjacency information were manually resolved, the most complex being for *A. funestus* with the reconciliation of synteny, RNAseq (AGOUTI & RASCAF), PacBio-AfunF2-IP-alignment, and physical mapping data, and the construction of a new cytogenetic photomap.

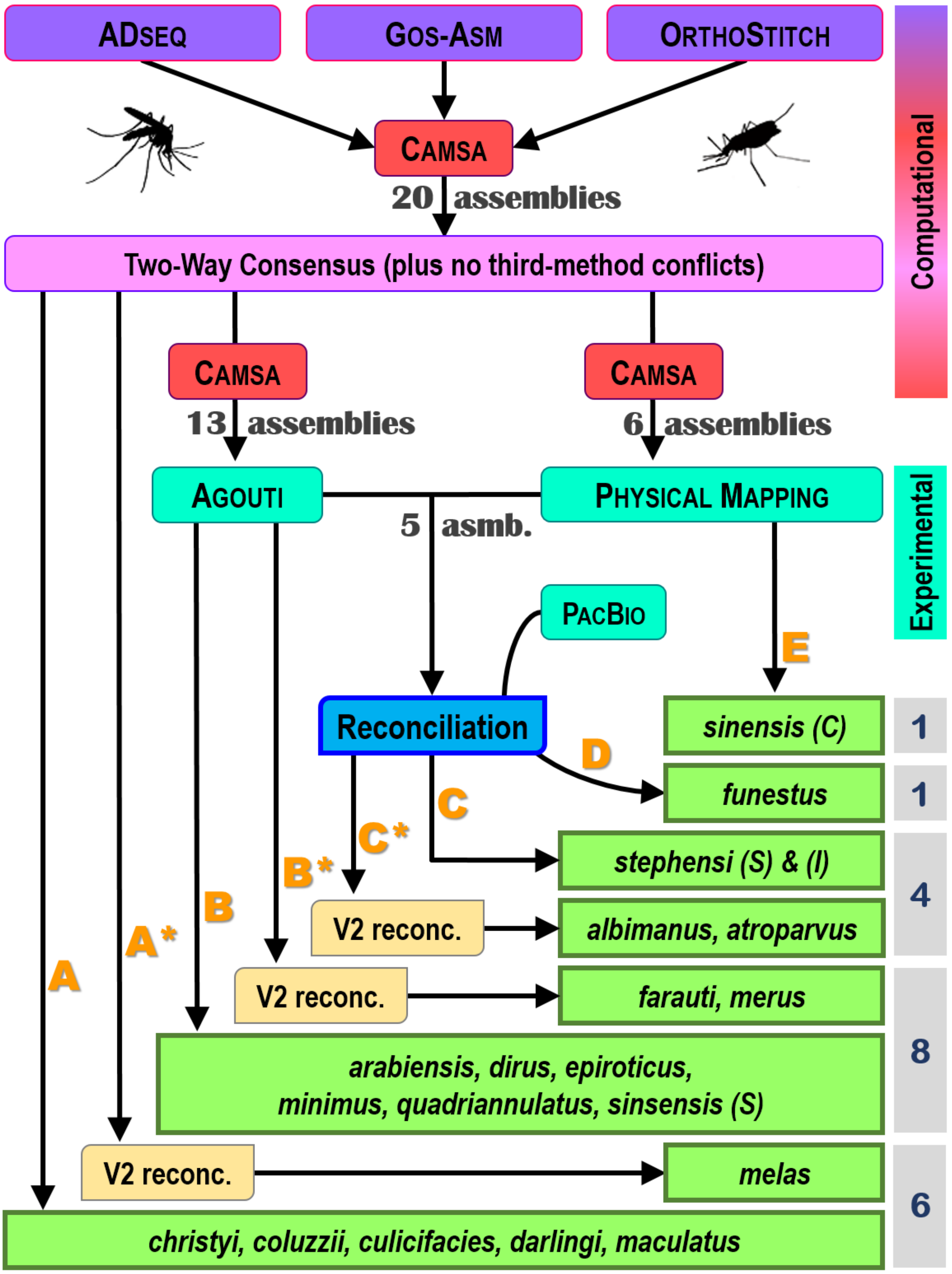

Figure S1. Workflows applied to upgrade the 20 anopheline assemblies

A: two-way synteny only. B: two-way synteny and AGOUTI. C: two-way synteny, AGOUTI, and physical mapping data. D: two-way synteny, AGOUTI, physical mapping data, and PacBio sequencing data. E: two-way synteny and physical mapping data. Asterisks (*) indicate additional reconciliation with version 2 assemblies (V2 reconc.) for a subset of species.

[2] Superscaffolding and chromosome arm assignments

Robert M. Waterhouse, Livio Ruzzante, Maarten J.M.F. Reijnders, Romain Feron

The integrated approach to reconciling the different sources of scaffold adjacencies with available experimental data outlined above and detailed in the sections below improved assembly contiguity through building well-supported superscaffolds (**Table 1, main text**). For several assemblies, the superscaffolding also resulted in the recovery of additional 'complete' Benchmarking Universal Single-Copy Orthologues (BUSCOs) (Simão et al. 2015; Waterhouse et al. 2018, 2019) (**Table S1**), indicating that superscaffolding helped to recover some genes that previously appeared to be fragmented or missing. Large increases in the numbers of recoverable BUSCOs are not expected as superscaffolding does not add new genomic sequence to the assemblies, but at least some partial genes at scaffold extremities now appear to be recoverable as 'complete' gene models.

Table S1. Assessments of assemblies with Benchmarking Universal Single-Copy Orthologues

Assessments with BUSCO v3.0.2 using the diptera_odb9 dataset (2799 BUSCOs). +/- numbers in parentheses indicate increases or decreases in the superscaffolded assemblies compared with chromosome or scaffold assemblies. See **Tables S2** and **S3** for the *Anopheles* species that corresponds to each assembly identifier.

Assembly	Status	Complete	Complete Single-Copy	Complete Duplicated	Fragmented	Missing
AalbS2	Chromosomes	2755 [98.4%]	2752 [98.3%]	3 [0.1%]	17 [0.6%]	27 [1.0%]
AalbS2	Scaffolds	2756 [98.5%]	2753 [98.4%]	3 [0.1%]	16 [0.6%]	27 [0.9%]
AalbS2	Superscaffolds	2756 [98.5%] (+1)	2753 [98.4%] (+1)	3 [0.1%] (0)	16 [0.6%] (-1)	27 [0.9%] (0)
AaraD1	Scaffolds	2752 [98.3%]	2747 [98.1%]	5 [0.2%]	19 [0.7%]	28 [1.0%]
AaraD1	Superscaffolds	2753 [98.4%] (+1)	2748 [98.2%] (+1)	5 [0.2%] (0)	16 [0.6%] (-3)	30 [1.0%] (+2)
AatrE1	Scaffolds	2753 [98.3%]	2741 [97.9%]	12 [0.4%]	27 [1.0%]	19 [0.7%]
AatrE3	Chromosomes	2749 [98.2%]	2737 [97.8%]	12 [0.4%]	23 [0.8%]	27 [1.0%]
AatrE3	Scaffolds	2753 [98.3%]	2741 [97.9%]	12 [0.4%]	27 [1.0%]	19 [0.7%]
AatrE3	Superscaffolds	2747 [98.1%] (-2)	2735 [97.7%] (-2)	12 [0.4%] (0)	25 [0.9%] (+2)	27 [1.0%] (0)
AchrA1	Scaffolds	2641 [94.3%]	2635 [94.1%]	6 [0.2%]	102 [3.6%]	56 [2.1%]
AchrA1	Superscaffolds	2654 [94.9%] (+13)	2647 [94.6%] (+12)	7 [0.3%] (+1)	92 [3.3%] (-10)	53 [1.8%] (-3)
AcolM1	Scaffolds	2509 [89.7%]	2504 [89.5%]	5 [0.2%]	158 [5.6%]	132 [4.7%]
AcolM1	Superscaffolds	2509 [89.6%] (0)	2505 [89.5%] (+1)	4 [0.1%] (-1)	159 [5.7%] (+1)	131 [4.7%] (-1)
AculA1	Scaffolds	2741 [97.9%]	2729 [97.5%]	12 [0.4%]	40 [1.4%]	18 [0.7%]
AculA1	Superscaffolds	2748 [98.2%] (+7)	2735 [97.7%] (+6)	13 [0.5%] (+1)	34 [1.2%] (-6)	17 [0.6%] (-1)
AdarC3	Scaffolds	2705 [96.7%]	2697 [96.4%]	8 [0.3%]	47 [1.7%]	47 [1.6%]
AdarC3	Superscaffolds	2709 [96.8%] (+4)	2701 [96.5%] (+4)	8 [0.3%] (0)	42 [1.5%] (-5)	48 [1.7%] (+1)
AdirW1	Scaffolds	2752 [98.4%]	2742 [98.0%]	10 [0.4%]	33 [1.2%]	14 [0.4%]
AdirW1	Superscaffolds	2753 [98.4%] (+1)	2743 [98.0%] (+1)	10 [0.4%] (0)	31 [1.1%] (-2)	15 [0.5%] (+1)
AepiE1	Scaffolds	2775 [99.2%]	2767 [98.9%]	8 [0.3%]	9 [0.3%]	15 [0.5%]
AepiE1	Superscaffolds	2773 [99.1%] (-2)	2765 [98.8%] (-2)	8 [0.3%] (0)	11 [0.4%] (+2)	15 [0.5%] (0)
AfarF2	Scaffolds	2741 [97.9%]	2736 [97.7%]	5 [0.2%]	34 [1.2%]	24 [0.9%]
AfarF2	Superscaffolds	2741 [97.9%] (0)	2736 [97.7%] (0)	5 [0.2%] (0)	34 [1.2%] (0)	24 [0.9%] (0)
AfunF1	Scaffolds	2758 [98.5%]	2743 [98.0%]	15 [0.5%]	26 [0.9%]	15 [0.6%]
AfunF1	Superscaffolds	2757 [98.5%] (-1)	2743 [98.0%] (0)	14 [0.5%] (-1)	27 [1.0%] (+1)	15 [0.5%] (0)
AfunF3	Chromosomes	2685 [96.0%]	2630 [94.0%]	55 [2.0%]	34 [1.2%]	80 [2.8%]
AgamP4	Chromosomes	2754 [98.3%]	2736 [97.7%]	18 [0.6%]	21 [0.8%]	24 [0.9%]
AgamP4	Scaffolds	2761 [98.6%]	2716 [97.0%]	45 [1.6%]	18 [0.6%]	20 [0.8%]
AmacM1	Scaffolds	1523 [54.5%]	1516 [54.2%]	7 [0.3%]	665 [23.8%]	611 [21.7%]
AmacM1	Superscaffolds	1547 [55.3%] (+24)	1539 [55.0%] (+23)	8 [0.3%] (+1)	636 [22.7%] (-29)	616 [22.0%] (+5)
AmelC2	Scaffolds	2582 [92.2%]	2497 [89.2%]	85 [3.0%]	153 [5.5%]	64 [2.3%]
AmelC2	Superscaffolds	2601 [93.0%] (+19)	2518 [90.0%] (+21)	83 [3.0%] (-2)	137 [4.9%] (-16)	61 [2.1%] (-3)
AmerM2	Scaffolds	2749 [98.2%]	2745 [98.1%]	4 [0.1%]	28 [1.0%]	22 [0.8%]
AmerM2	Superscaffolds	2754 [98.3%] (+5)	2750 [98.2%] (+5)	4 [0.1%] (0)	25 [0.9%] (-3)	20 [0.8%] (-2)
AminM1	Scaffolds	2767 [98.8%]	2761 [98.6%]	6 [0.2%]	18 [0.6%]	14 [0.6%]

AminM1	Superscaffolds	2764 [98.7%] (-3)	2758 [98.5%] (-3)	6 [0.2%] (0)	20 [0.7%] (+2)	15 [0.6%] (+1)
AquaS1	Scaffolds	2754 [98.4%]	2749 [98.2%]	5 [0.2%]	19 [0.7%]	26 [0.9%]
AquaS1	Superscaffolds	2753 [98.3%] (-1)	2747 [98.1%] (-2)	6 [0.2%] (+1)	21 [0.8%] (+2)	25 [0.9%] (-1)
AsinC2	Scaffolds	2688 [96.0%]	2671 [95.4%]	17 [0.6%]	64 [2.3%]	47 [1.7%]
AsinC2	Superscaffolds	2691 [96.2%] (+3)	2675 [95.6%] (+4)	16 [0.6%] (-1)	63 [2.3%] (-1)	45 [1.5%] (-2)
AsinS2	Scaffolds	2570 [91.8%]	2537 [90.6%]	33 [1.2%]	127 [4.5%]	102 [3.7%]
AsinS2	Superscaffolds	2573 [91.9%] (+3)	2540 [90.7%] (+3)	33 [1.2%] (0)	123 [4.4%] (-4)	103 [3.7%] (+1)
AsteI2	Scaffolds	2716 [97.0%]	2710 [96.8%]	6 [0.2%]	56 [2.0%]	27 [1.0%]
AsteI2	Superscaffolds	2710 [96.8%] (-6)	2704 [96.6%] (-6)	6 [0.2%] (0)	59 [2.1%] (+3)	30 [1.1%] (+3)
AsteS1	Scaffolds	2753 [98.3%]	2722 [97.2%]	31 [1.1%]	29 [1.0%]	17 [0.7%]
AsteS1	Superscaffolds	2752 [98.3%] (-1)	2721 [97.2%] (-1)	31 [1.1%] (0)	28 [1.0%] (-1)	19 [0.7%] (+2)

The superscaffolded assemblies also allowed for enhancing the anchoring of ordered and oriented scaffolds to chromosome arms (**Table 2, main text**), and the assignment of non-anchored scaffolds and superscaffolds to chromosome arms (**Table S2; Additional File 2**). The resulting superscaffolds had total spans ranging from more than 200 Mbps for *A. arabiensis* to fewer than 20 Mbps for *A. maculatus*, reflecting the contiguity of the input assemblies and the availability of complementary datasets to support superscaffolding (**Figure S2**). For ten assemblies the total span of superscaffolds comprised more than half the total assembly size, and they made up more than a quarter of a further seven assemblies (**Figure S2**). The enhanced chromosome anchoring for a subset of the anophelines (**Table 2, main text**) and the chromosomal-level assembly for *A. gambiae* PEST together allowed for the assignment of non-anchored scaffolds and superscaffolds to chromosome arms. Enumerating shared orthologues between non-anchored scaffolds and the eight species with chromosome-anchored scaffolds (see section [12] below for details) enabled assignments with support from multiple species (**Table S2; Additional File 2**).

Table S2. Assignment of scaffolds and superscaffolds to chromosome arms
Scaffold counts and proportions of the 20 updated assemblies with chromosome arm assignments.

Species	Assembly Version	Assigned Scaffolds or Superscaffolds [Also Anchored]	% Assembly Assigned
Anopheles albimanus	AalbS3	7 [7]	97
Anopheles arabiensis	AaraD2	5 [5]	88
Anopheles atroparvus	AatrE4	10 [10]	88
Anopheles christyi	AchrA2	154	8
Anopheles coluzzii	AcolM2	65	85
Anopheles culicifacies	AculA2	286	19
Anopheles darlingi	AdarC4	247	48
Anopheles dirus	AdirW2	36	91
Anopheles epiroticus	AepiE2	215	78
Anopheles farauti	AfarF3	29	97
Anopheles funestus	AfunF2	136 [81]	90
Anopheles maculatus	AmacM2	2	0
Anopheles melas	AmelC3	106	4
Anopheles merus	AmerM3	119	75
Anopheles minimus	AminM2	22	96
Anopheles quadriannulatus	AquaS2	105	80
Anopheles sinensis	AsinS3	222	56
Anopheles sinensis (Chinese)	AsinC3	165 [29]	70
Anopheles stephensi	AsteS2	150 [71]	89
Anopheles stephensi (Indian)	AsteI3	72 [60]	83

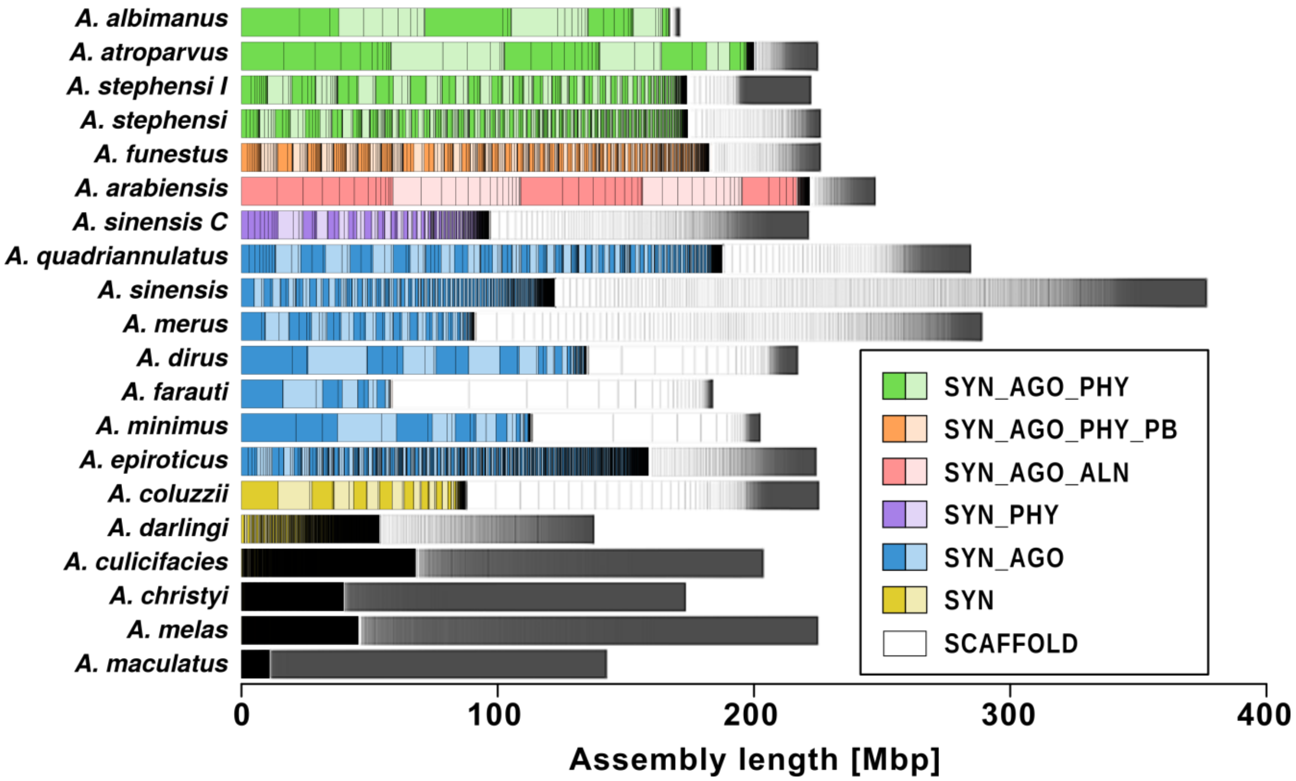

Figure S2. Superscaffolding genomic spans of 20 anopheline genome assemblies

Superscaffolds are shown as stacked bars of alternating dark and light colours with lines within each superscaffold indicating the sizes (y-axis, basepairs) of their constituent scaffolds, and with superscaffolds and scaffolds ordered from the largest (left) to the smallest (right). The stacked bars continue with scaffolds that are not part of superscaffolds in grey, again ordered from the largest to the smallest. The assemblies are grouped and coloured according to the types of data and approaches used to perform the superscaffolding as presented in the legend and in main text Table 1. Approaches: synteny-based (SYN), and/or RNAseq AGOUTI-based (AGO), and/or alignment-based (ALN), and/or physical mapping-based (PHY), and/or PacBio sequencing-based (PB). Results for two strains are shown for *Anopheles sinensis*, SINENSIS and Chinese (C), and *Anopheles stephensi*, SDA-500 and Indian (I).

The local impact of superscaffolding on improving the ability to identify syntenic orthologues between pairs of assemblies was assessed by enumerating pairs and trios of collinear orthologues before and after superscaffolding (**Figure S3**). From the full set of orthologous groups delineated across the 21 *Anopheles* assemblies (detailed in section [3] below), a subset of 10'657 groups were selected with orthologues in more than half of the assemblies and with more than half of these being single-copy orthologues. Being widely present and most single-copy, these orthologous groups represent a relatively evolutionarily stable

set of genes with which to assess local synteny. For each assembly, neighbouring pairs and trios of these genes with orthologues that were maintained as neighbours in the other assemblies were counted before and after superscaffolding. Comparing each superscaffolded assembly with its input assembly showed the greatest gains of almost 3’000 pairs and about 2’000 trios for *A. culicifacies*, *A. christyi*, and *A. melas*, all of which were built following workflow A (i.e. only synteny-based adjacencies). The global impact of superscaffolding is exemplified by comparing orthologue locations in the *A. gambiae* (PEST) genome and the new *A. arabiensis* assembly to reveal large-scale structural variants (**Figure S4**) that confirm the rearrangements identified from the previous scaffold-level assembly for *A. arabiensis* that was used to explore patterns of introgression in the species complex (Fontaine et al. 2015) and known from previous polytene chromosome studies (Coluzzi et al. 2002).

	Trios	AALBS	ADARC	AATRE	ASINC	ASINS	ADIRW	AFARF	AMACM	ASTEI	ASTES	AFUNF	ACULA	AMINM	AEPIE	ACHRA	AMERM	AMELC	AQUAS	AARAD	ACOLM	AGAMP	
Pairs		42	413	33	90	61	42	149	394	118	198	233	987	22	379	915	222	830	114	28	33	12	AALBS
AALBS	46		563	348	292	199	326	378	363	373	373	402	728	337	421	702	334	695	359	272	311	333	ADARC
ADARC	710	758		60	138	101	48	227	510	139	212	253	1197	31	397	1061	264	1054	137	25	52	25	AATRE
AATRE	72	636	100		186	153	121	228	493	191	213	254	979	96	388	902	219	906	174	75	118	86	ASINC
ASINC	140	584	208	218		172	85	158	360	125	144	193	699	83	243	636	113	623	129	67	102	70	ASINS
ASINS	196	474	252	302	317		59	279	553	188	271	355	1382	48	539	1288	245	1126	169	39	62	41	ADIRW
ADIRW	70	632	90	184	224	72		466	547	353	431	448	1366	245	599	1200	414	1174	317	192	231	189	AFARF
AFARF	228	680	340	368	362	374	520		807	654	639	573	637	611	637	540	573	483	579	509	513	632	AMACM
AMACM	1763	1536	2047	1941	1549	2164	2111	2680		229	391	433	1427	179	623	1347	352	1193	267	130	164	125	ASTEI
ASTEI	196	700	232	298	280	282	496	2310	280		370	545	1530	291	703	1434	399	1250	348	181	246	159	ASTES
ASTES	278	696	322	326	344	356	558	2346	514	430		496	1509	358	719	1308	439	1166	411	222	292	239	AFUNF
AFUNF	339	753	370	406	391	436	617	2209	582	676	578		2001	1541	1544	1259	1204	1261	1349	1055	1227	1255	ACULA
ACULA	1971	1509	2165	1871	1441	2390	2337	2248	2429	2596	2542	2957		28	539	1367	281	1187	162	31	54	5	AMINM
AMINM	30	626	64	148	212	68	318	2255	250	348	416	2565	28		710	1550	531	1329	575	366	445	417	AEPIE
AEPIE	548	804	604	584	526	716	822	2333	848	906	940	2605	692	811		1846	1130	1032	1340	1143	1173	1369	ACHRA
ACHRA	1970	1540	2154	1924	1404	2404	2316	1866	2468	2584	2426	2417	2468	2710	2942		963	1195	325	163	341	237	AMERM
AMERM	316	619	357	289	280	298	514	2108	474	499	564	2041	311	692	2084	1105		1728	1243	1048	1191	1243	AMELC
AMELC	1890	1580	2140	1918	1464	2282	2287	1880	2358	2446	2385	2669	2374	2568	2236	2225	2997		243	146	156	135	AQUAS
AQUAS	170	666	214	280	304	232	442	2144	394	458	536	2312	214	768	2395	368	2380	296		97	56	39	AARAD
AARAD	46	504	64	136	192	74	282	1926	206	250	333	1842	50	522	2071	232	2040	216	128		88	57	ACOLM
ACOLM	98	588	136	226	264	160	378	2030	304	388	447	2188	130	668	2236	412	2352	246	118	156		0	AGAMP
AGAMP	18	583	52	142	198	56	284	2263	192	247	362	2134	10	586	2392	271	2282	188	70	130	0		Trios
	AALBS	ADARC	AATRE	ASINC	ASINS	ADIRW	AFARF	AMACM	ASTEI	ASTES	AFUNF	ACULA	AMINM	AEPIE	ACHRA	AMERM	AMELC	AQUAS	AARAD	ACOLM	AGAMP	Pairs	

Figure S3. Increases in pairs and trios of syntenic orthologues after superscaffolding

Heatmaps of counts of additional neighbouring pairs (below the diagonal, from blue=low to yellow=high) and trios (above the diagonal, from purple=low to red=high) of genes with orthologues maintained as neighbours in pairs of assemblies after superscaffolding. The outlined cells along the diagonal present gained pairs and trios for each superscaffolded assembly compared with its input assembly. See **Table S3** for the species that corresponds to each assembly abbreviation.

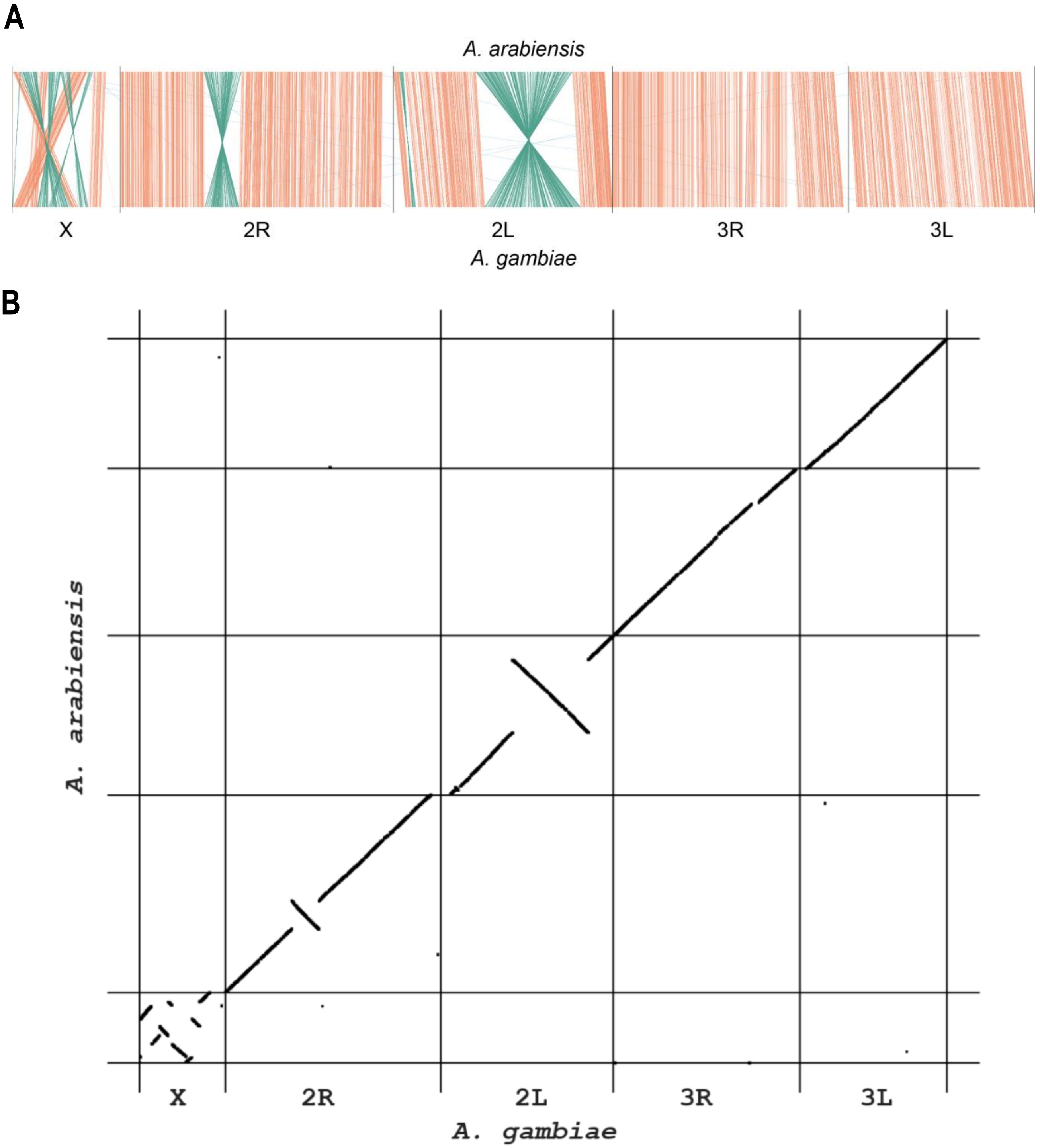

Figure S4. Structural variants between *Anopheles gambiae* and *Anopheles arabiensis*

(A) Lineplot showing the genomic locations of single-copy orthologues between *Anopheles gambiae* and *Anopheles arabiensis* are shown connected with orange lines for contiguous regions, green for inversions, and blue for arm translocations. **(B)** Traditional dotplot view of the locations of the same single-copy orthologues. These comparisons confirm the structural variants identified from the previous scaffold-level assembly for *A. arabiensis* that was used to explore patterns of introgression in the species complex (Fontaine et al. 2015).

[3] Sources of input data for predicting adjacencies

Robert M. Waterhouse

The orthology data used as inputs for each of the three synteny-based methods were retrieved from ORTHODB V9.1 (www.orthodb.org) (Zdobnov et al. 2017). These orthologous groups included all the anophelines apart from *A. sinensis* SINENSIS strain and *A. stephensi* Indian strain, so proteins from the gene sets of these two anophelines were mapped to the ORTHODB anopheline orthologous groups using the complete species mapping approach of ORTHODB. The protein sequences used by ORTHODB, and the gene annotations required for the adjacency predictions, were retrieved from VECTORBASE (Giraldo-Calderón et al. 2015). The versions of the genome assemblies and their annotated gene sets are detailed in **Table S3**, along with counts of scaffolds, genes, and orthologues.

Table S3. Assembly and orthology input data

Genome assembly versions, scaffold counts, gene set versions, gene counts, and ORTHODB orthologous groups (from ORTHODB V9.1) across 21 anophelines used as input data for the synteny-based scaffold adjacency predictions.

Species	Assembly	Scaffolds	Gene set	Total genes	Genes in orthogroups	Scaffolds with genes	Scaffolds with orthologs
Anopheles albimanus	AalbS1	204	AalbS1.3	12085	10637	57	52
Anopheles arabiensis	AaraD1	1214	AaraD1.3	13333	12132	340	289
Anopheles atroparvus	AatrE1	1371	AatrE1.3	13789	12249	476	384
Anopheles christyi	AchrA1	30369	AchrA1.2	10738	10103	5173	5064
Anopheles coluzzii	AcolM1	10521	AcolM1.2	14710	12998	1124	816
Anopheles culicifacies	AculA1	16162	AculA1.2	14335	13002	5715	5200
Anopheles darlingi	AdarC3	2221	AdarC3.2	10457	9871	2161	2055
Anopheles dirus	AdirW1	1266	AdirW1.3	12840	11488	302	250
Anopheles epiroticus	AepiE1	2673	AepiE1.3	12181	11549	1053	1004
Anopheles farauti	AfarF1	550	AfarF1.2	13217	12146	376	355
Anopheles funestus	AfunF1	1392	AfunF1.2	13344	11616	619	575
Anopheles gambiae	AgamP4	8	AgamP4.2	12843	12240	7	7
Anopheles maculatus	AmacM1	47797	AmacM1.2	14835	11777	12776	10297
Anopheles melas	AmelC1	20281	AmelC1.2	16149	14718	8855	8223
Anopheles merus	AmerM1	2753	AmerM1.2	13886	13076	1078	1036
Anopheles minimus	AminM1	678	AminM1.3	12663	11436	142	121
Anopheles quadriannulatus	AquaS1	2823	AquaS1.3	13484	12055	647	576
Anopheles sinensis	AsinS2	10448	AsinS2.1	12869	11037	1825	1486
Anopheles sinensis (Chinese)	AsinC2	9592	AsinC2.1	19352	11594	702	573
Anopheles stephensi	AsteS1	1110	AsteS1.3	13227	11645	502	479
Anopheles stephensi (Indian)	AsteI2	23371	AsteI2.2	11789	11100	906	660

The three synteny-based methods described below in sections **[4] ADSEQ**, **[5] GOS-ASM**, and **[6] ORTHOSTITCH** share the overarching goal of identifying blocks of collinear orthologues across several species that can be used to infer scaffold adjacencies in species where this collinearity has been broken due to assembly fragmentation. They operate in a framework where multiple rearrangements over the course of evolution have gradually eroded the collinearity of extant genomes with the ancestral organisation into shorter synteny blocks. Within these synteny blocks, broken collinearity in one or more species delineates putative rearrangement breakpoints, which may range in age from events that occurred early in the species radiation to younger lineage- or species-specific rearrangement events. Once these breakpoints have been identified, the methods then attempt to decide whether an observed breakpoint in an extant genome is the result of a true genomic rearrangement event or the result of assembly fragmentation, considering breakpoints at the extremities of contigs/scaffolds to be more likely due to assembly fragmentation than to true genomic rearrangement events.

[4] ADSEQ: scaffolding genomes using gene trees, synteny and sequencing data

Yoann Anselmetti, Sèverine Bérard, Eric Tannier, Cedric Chauve

Full descriptions of the algorithms implemented, underlying assumptions, and performance of ADSEQ are detailed in (Duchemin et al. 2017; Anselmetti et al. 2015, 2018). ADSEQ implements extensions to a group of approaches that aim to reconstruct evolutionary histories of gene adjacencies, based on the DECO algorithm (Bérard et al. 2012). ADSEQ computes ancestral genome segments and extant scaffolding adjacencies, taking advantage of sequencing data (e.g. paired-end reads) if available, and enabling inferences of various evolutionary events including gene duplications/losses/translocations along each branch of the provided species phylogeny. Previous simulations, described in (Anselmetti et al. 2018), of assembly fragmentation using a subset of *Anopheles* genomes have detailed performance of ADSEQ in terms of precision and recall statistics, including comparisons with the scaffolder BESST (Sahlin et al. 2014). Similarly, ART-DECO analyses, detailed in (Anselmetti et al. 2015), simulated fragmentation of tetrapod genomes to evaluate the ability to recover broken scaffold adjacencies.

Gene trees. Gene trees contain the information about how genes, and the traits they are related to, evolve along the history of the species. They give access to information about adaptations by substitutions, gene gains and losses, duplications, transfers. Gene trees can also be used to detect co-evolutionary elements in genomes. Moreover, gene trees are useful to reconstruct ancestral genomes and provide better assemblies for extant species, as shown in (Duchemin et al. 2017; Anselmetti et al. 2015,

2018). However, the quality of the results highly depends on the quality of the gene trees. For the *Anopheles* genomes, genes were clustered into ORTHODB orthologous groups (**Table S3**), and multiple alignments were computed for each group using MUSCLE v.3.8.425 (Edgar 2004). These were then used as input for RAXML (Stamatakis 2014) phylogenetic tree estimations, in a large-scale automatic effort. A substantial number of branches are probably incorrect, since multiple sequence alignments often do not contain enough signal to fully resolve the gene tree. We applied the gene tree correction program TREERECS (https://gitlab.inria.fr/Phylophile/Treerecs) to correct these gene trees and our preliminary analysis shows that the corrected trees are of better quality (in terms of ancestral gene content) than the original ones.

Scaffolding extant and ancestral genomes. In a second step we used these improved reconciled gene trees to reconstruct jointly ancestral and extant gene adjacencies. The general approach is described in our recent papers (Anselmetti et al. 2015, 2018): we consider pairs of gene families for which extant adjacencies (synteny) is observed and compute, from the reconciled gene trees, a duplication-aware parsimonious evolutionary scenario in terms of adjacency gain/breaks that can also create extant adjacencies between genes at the extremities of contigs/scaffolds. The method has been modified to include sequencing data for the inference of potential extant scaffolding adjacencies, thus it is based on a combination of evolutionary signal and sequence data. We used all sequencing data available for the 21 anophelines to associate a prior score to potential extant scaffolding adjacencies with the scaffolder BESST (Sahlin et al. 2014). The new method, using both sentence evolution and sequencing data is called ADSEQ; it includes a probabilistic version of the algorithm that allows sampling of optimal solutions uniformly and to associate to potential scaffolding (both extant and ancestral) a posterior score defined as the frequency of observing the adjacencies in this sample. Finally, if adjacency conflicts are observed (e.g. the same contig extremity is deemed to be adjacent to more than one other contig extremity), we use a Maximum Weight Matching algorithm to resolve these conflicts, using the posterior score of the adjacencies as edge weights. Resulting counts of predicted scaffold adjacencies for each of the anopheline assemblies are presented in **Table S4**.

Data and code availability. Input data and results obtained with ADSEQ are available from the GitHub repository https://github.com/YoannAnselmetti/DeCoSTAR_pipeline in the directory named "21Anopheles_dataset". This contains a pipeline written in snakemake, a python workflow management system (Köster and Rahmann 2012), allowing users to generate input data required for ADSEQ and execute it from standard genomic format files. Input gene trees and adjacencies were produced from ORTHODB orthologous groups and gene locations available in **Additional File 3**.

[5] GOS-ASM: multi-genome rearrangement-based gene order scaffolder

Sergey Aganezov, Max A. Alekseyev

Full descriptions of the algorithms implemented, underlying assumptions, and performance of GOS-ASM are detailed in (Aganezov et al. 2015; Aganezov and Alekseyev 2016; Avdeyev et al. 2016). GOS-ASM reconstructs global gene orders along chromosomes from gene sub-orders along scaffolds, relying on the concept of the multiple breakpoint graph as developed for the Multiple Genome Rearrangements and Ancestors (MGRA) reconstruction tool (Alekseyev and Pevzner 2009; Avdeyev et al. 2016). These gene sub-orders are interpreted as arising from both evolutionary rearrangement events and technological fragmentation (i.e. assembly failures), where the latter is modelled by artificial 'fissions' that break the chromosome-level gene orders into scaffold-level gene sub-orders. GOS-ASM performs superscaffolding by searching for putative 'fusions' that revert such technological 'fissions' and join fragmented scaffolds back together. Previous simulations of assembly fragmentation separately using *Anopheles* and mammalian genomes have detailed performance of GOS-ASM in terms of true and false positive rates and are described in (Aganezov et al. 2015).

GOS-ASM (Aganezov and Alekseyev 2016) (https://github.com/aganezov/gos-asm, Gene order scaffold assembler) starts with an assumption that the constructed scaffolds that make up the input assemblies are accurate and long enough to allow for the identification of orthologous genes. The scaffolds can then be represented as ordered sequences of oriented genes and the scaffold assembly problem can be posed as the reconstruction of the global gene order (along genome chromosomes) from the gene sub-orders defined by the scaffolds. Such gene sub-orders are viewed as the result of both evolutionary events and artificial "technological" fragmentation in the genome. Evolutionary events that change gene orders are genome rearrangements, most common of which are reversals, fusions, fissions, and translocations. Technological fragmentation is modelled by artificial "fissions" that break genomic chromosomes into scaffolds. Scaffold assembly can therefore be reduced to the search for "fusions" that revert technological "fissions" and glue scaffolds back into chromosomes. This observation inspired us to employ the genome rearrangement analysis techniques for scaffolding purposes. Rearrangement analysis of multiple genomes relies on the concept of the breakpoint graph and utilizes the topology of the organisms' phylogenetic tree. While traditionally the breakpoint graph is constructed for complete genomes, it can also be constructed for fragmented genomes, where we treat scaffolds as "chromosomes". We demonstrate that the breakpoint graph of multiple genomes possesses an important property that its connected components are robust with respect to the technological genome fragmentation. In other words, connected components of the breakpoint graph mostly retain information about the complete genomes, even when the breakpoint graph is constructed on their scaffolds. We thus use the topology of the species phylogenetic tree and the structure of the connected components in the corresponding breakpoint graph

to reconstruct the "reverse evolution" of the input genomes along the branches of the phylogenetic tree, distinguishing between signatures of evolutionary and technological fissions. Identified technological fissions are then used as guidance for the gluing of input scaffolds back into complete chromosomes. Resulting counts of predicted scaffold adjacencies from applying GOS-ASM to the full set of anopheline assemblies are presented in **Table S4**.

[6] ORTHOSTITCH: scaffold adjacencies from conserved orthologous neighbours

Robert M. Waterhouse

Using gene orthology data from cross-species comparisons, ORTHOSTITCH identifies genes located at scaffold extremities and evaluates the evidence from the locations of orthologous genes from other species to predict likely scaffold adjacencies. The analysis proceeds in a stepwise manner, first identifying the most likely neighbour for each scaffold end and then requiring best neighbours to be reciprocal in order to identify putative adjacencies. The evaluations are not limited to single-copy orthologues as analyses of all paralogues are performed such that all possible neighbour relationships are examined. Putative neighbours at scaffold extremities are scored by how many of the species with orthologues show the same neighbour relationship (**Figure S5**), requiring at least two species to do so. ORTHOSTITCH was developed as part of the synteny-focused analyses of the comparative analysis of the *Manduca sexta* genome (Kanost et al. 2016), it is described in detail below and the code is available from the GitLab project page: https://gitlab.com/rmwaterhouse/OrthoStitch

ORTHOSTITCH requires as input an anchor groups file and an anchor locations file. The anchor groups file may be generated from any orthology delineation procedure, and consists of just three columns of data: the orthologous group identifier, the gene identifier, and the species identifier. The anchor locations file may be generated from general feature format (GFF) or general transfer format (GTF) files that indicate the genomic locations of annotated features (genes) for each assembly. Like GFF or GTF files, the anchor locations file consists of nine columns, with only the coding sequence (CDS) lines selected from GFF or GTF files, and with the 'source' column (2nd column) containing the species identifier, and with the 'attribute' column (9th column) containing only the gene identifier. The gene and species identifiers used in both the groups file and the locations file must match exactly, and gene identifiers must be unique across the complete dataset of all species. The anchor locations file may contain the locations of genes that are not present in the anchor groups file, i.e. some genes with known locations

may not have been assigned to any orthologous group, however, the anchor groups file may not contain any genes that are not present in the anchor locations files, i.e. all genes in orthologous groups must have known locations.

Species	Scaffold	GeneID [GroupID] #neighbours		GeneID [GroupID] #neighbours	Scaffold
AFUNE	KB668690 [+]	AFUN010217 [EOG09170540] 1	---x>+-x-->	AFUN000326 [EOG091701PP] 1	KB668920 [+]
AALBI	KB672397	AALB002374 [EOG09170540] 2	--x--o--x--	AALB002371 [EOG091701PP] 2	KB672397
AARAB	KB704451	AARA004744 [EOG09170540] 2	---x---x---	AARA004745 [EOG091701PP] 2	KB704451
AATRO	KI421897	AATE015926 [EOG09170540] 2	---x---x---	AATE019573 [EOG091701PP] 2	KI421897
ACHRI	KB698096	ACHR007688 [EOG09170540] 0	-x--	noortho [NOOG]	na
ACOLU	scf_1925491386	ACOM037460 [EOG09170540] 2	---x---x---	ACOM037465 [EOG091701PP] 2	scf_1925491386
ACULI	KI423732	ACUA013460 [EOG09170540] 0	-x-- --x-	ACUA014152 [EOG091701PP] 1	KI424031
ADARL	na	noortho [NOOG]	.... --x-	ADAC004066 [EOG091701PP] 2	scaffold_20
ADIRU	KB672868	ADIR002642 [EOG09170540] 2	---x---x---	ADIR002641 [EOG091701PP] 2	KB672868
AEPIR	KB672164	AEPI009033 [EOG09170540] 2	-x-- --x-	AEPI005575 [EOG091701PP] 2	KB671247
AFARA	KI421545	AFAF012506 [EOG09170540] 2	---x---x---	AFAF012254 [EOG091701PP] 2	KI421545
AGAMB	2R	AGAP002925 [EOG09170540] 2	---x---x---	AGAP002926 [EOG091701PP] 2	2R
AMACU	AXCL01014811	AMAM000110 [EOG09170540] 0	-x-- --x-	AMAM010795 [EOG091701PP] 0	AXCL01051139
AMELA	KI429153	AMEC010445 [EOG09170540] 1	---x---x---	AMEC021444 [EOG091701PP] 2	KI429153
AMERU	KI438982	AMEM010727 [EOG09170540] 2	---x---x---	AMEM012929 [EOG091701PP] 2	KI438982
AMINI	KB663832	AMIN000747 [EOG09170540] 2	---x---x---	AMIN000748 [EOG091701PP] 2	KB663832
AQUAD	KB666065	AQUA009280 [EOG09170540] 2	---x---x---	AQUA009279 [EOG091701PP] 2	KB666065
ASINC	AS2_scf7180000695544	ASIC004586 [EOG09170540] 2	--x--o--x--	ASIC004611 [EOG091701PP] 2	AS2_scf7180000695544
ASINS	KI916183	ASIS000523 [EOG09170540] 2	--x--o--x--	ASIS000668 [EOG091701PP] 2	KI916183
ASTEI	scaffold_00001	ASTEI00055 [EOG09170540] 2	---x---x---	ASTEI00054 [EOG091701PP] 2	scaffold_00001
ASTES	KB665265	ASTE007167 [EOG09170540] 2	---x---x---	ASTE007166 [EOG091701PP] 2	KB665265

Figure S5. Example of ORTHOSTITCH adjacency evidence

This putative adjacency is identified in *A. funestus* (AFUNE, blue) with both scaffolds in the forward orientation, where orthologous genes from 12 other anophelines support the neighbour relationship (green). In three other anophelines one or more intervening genes disrupt the neighbour relationship of these pairs of orthologues (orange). In the remaining five anophelines there are no orthologues or the orthologues have no neighbouring genes and thus they offer neither support nor evidence against the putative neighbour relationship (yellow), or there are orthologues with neighbours but they do not support the putative adjacency (purple). So this adjacency is supported by evidence from 12 species out of a possible 16 for scaffold KB668690 and out of a possible 18 for scaffold KB668920, giving a synteny score of 0.71 and a universality score of 0.85 with a final adjacency score of 0.60.

ORTHOSTITCH options allow for the genomic location of each anchor gene to be set as the start, middle, or end of the input coding sequence genomic coordinates, and the analyses can be run using only genes with orthologues or with all genes in the locations file. All predicted adjacencies are further classified into confident, and superconfident subsets. Confident adjacencies require more than a third of comparison species to have orthologues and more than a third of those that do have orthologues to support the predicted scaffold adjacency. Superconfident adjacencies additionally require the same of their upstream or downstream neighbours. The adjacency score for each pair of putatively neighbouring scaffolds is computed as the product of a synteny score *(S)* and a universality score *(U)*, based on the numbers of

species with orthologues that support the adjacency where *Sup* = the number of supporting species, *Pos* = the number of possible species, and *Tot* = the total number of species thus:

$$S = \frac{\left(\frac{Sup1}{Pos1} + \frac{Sup2}{Pos2}\right)}{2} \qquad U = \frac{\left(\frac{Pos1 + Pos2}{2}\right)}{Tot - 1}$$

Orthology data from ORTHODB v9 (Zdobnov et al. 2017), were used to produce the input anchor groups file and the anchor locations were produced from GFF files from VECTORBASE (Giraldo-Calderón et al. 2015) (see **Table S3**). The ORTHOSTITCH (v1.6) analysis was run using data from all 21 available anophelines with the options of anchor locations set to 'middle' and using all annotated genes, and the resulting adjacency counts are presented in **Table S4**.

The performance of ORTHOSTITCH in terms of the ability to recover true adjacencies versus false adjacencies was assessed using the same input dataset from the 21 anophelines with the introduction of artificial scaffold/chromosome breaks. Four different types of randomly positioned scaffold/chromosome-splitting breaks were introduced and analysed separately, (i) between any (ANY) neighbouring pair of orthologues; (ii) between neighbouring orthologue pairs both from orthologous groups containing at least a third (1/3) of the 21 species; (iii) between neighbouring orthologue pairs both from orthologous groups with more than half (1/2) of the 21 species, a gene-to-species ratio of no more than 1.5 (i.e. limiting the numbers of duplicated copies), and restricted to scaffolds/chromosomes with at least 25 orthologues in total (i.e. avoiding splitting shorter scaffolds); and (iv) the same as (iii) but also requiring the neighbouring pair to have been part of the supporting sets that defined the superconfident adjacencies (1/2+SYN) in **Table S4** (i.e. known to provide synteny support). 100 random scaffold/chromosome breaks were introduced and then analysed to predict putative adjacencies and assess how many of the artificially introduced breaks were correctly recovered as predicted adjacencies and how many were incorrectly recovered, repeated 100 times for each of the four different types of neighbouring orthologues. True adjacencies are those that correctly predict the split pairs of orthologues as neighbours, false adjacencies are those that incorrectly predict a different neighbour for either or both of the split orthologues. These were assessed for the 'all' and 'confident' sets of adjacencies predicted by ORTHOSTITCH.

ORTHOSTITCH options were selected as for the complete analysis above, with anchor locations set to 'middle' and using all annotated genes. Median true recoveries for the sets of all adjacencies were 74%, 82%, 87%, and 96% for the four split types, ANY, 1/3, 1/2, 1/2+SYN, respectively, versus median false recoveries for the same sets of 2, 2, 2, and 1 (**Figure S6**). True recoveries increased according to split type from ANY to 1/3 to 1/2 to 1/2+SYN, as more orthologues and more syntenic orthologues at

breakpoints allow for better predictions. True recoveries decreased for the confident datasets as the more stringent prediction criteria filter out real adjacencies. False recoveries were very low across all analysed datasets, with a few more from the all versus the confident predictions. Thus for similar datasets ORTHOSTITCH is expected to be able to recover about three quarters of true adjacencies, when the genes at the scaffold extremities have orthologues in more than a third or more than half the species then recovery levels are expected to increase, and when these orthologues provide synteny support then the adjacencies are almost always recovered.

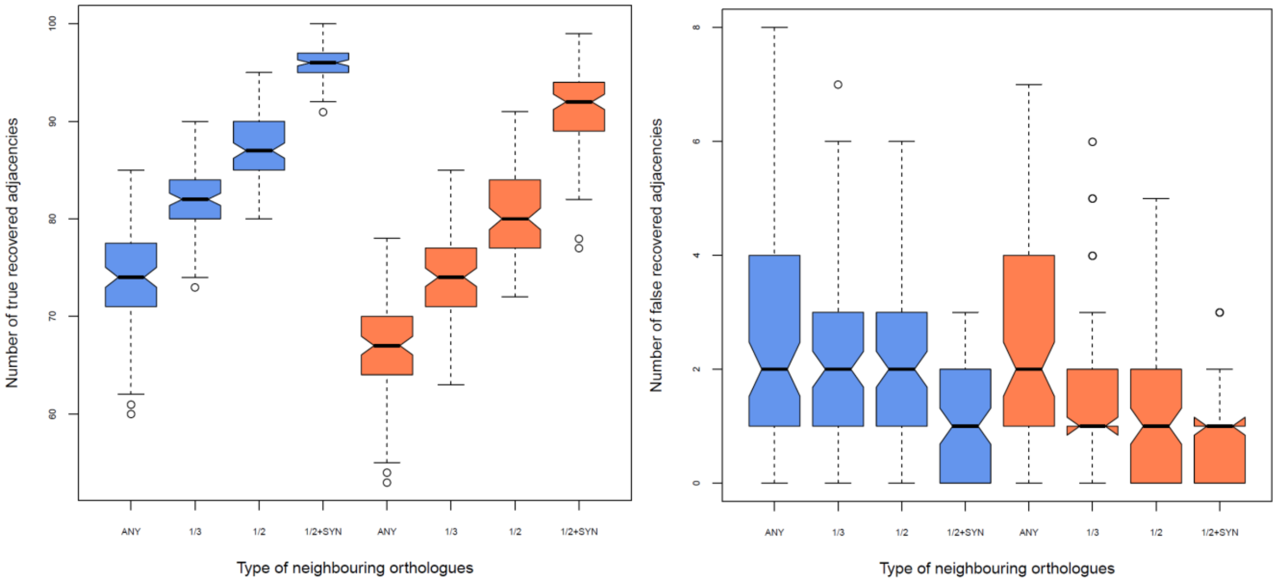

Figure S6. Performance of ORTHOSTITCH adjacency recovery

For each of four different types of neighbouring orthologues (ANY, 1/3, 1/2, 1/2+SYN, see text for details), a total of 100 random scaffold/chromosome breaks were introduced into the gene locations data. These were then analysed to predict putative adjacencies and assess how many introduced breaks were recovered as predicted adjacencies. This was repeated 100 times for each of the four different types of neighbouring orthologues. Results were assessed for two levels of confidence estimated by OrthoStitch, namely all (blue) and confident (orange) adjacencies to enumerate true recovered adjacencies (left panel), i.e. those that correctly predict the split pairs of orthologues as neighbours, and false recovered adjacencies (right panel), i.e. those that incorrectly predict a different neighbour for either or both of the split orthologues.

Table S4. Synteny-based adjacency predictions
Counts of predicted adjacencies from running three methods across 21 anophelines.

Species	ADSEQ	GOS-ASM	ORTHOSTITCH		
			All	Confident	Superconfident
Anopheles albimanus	1	2	4	4	3
Anopheles arabiensis	48	11	33	27	7
Anopheles atroparvus	42	6	29	24	2
Anopheles christyi	3220	1176	2031	1820	371
Anopheles coluzzii	199	71	134	114	14
Anopheles culicifacies	3594	2055	1821	1620	373
Anopheles darlingi	678	290	684	551	109
Anopheles dirus	63	19	42	36	10
Anopheles epiroticus	608	143	471	369	190
Anopheles farauti	177	75	119	92	36
Anopheles funestus	331	100	211	167	78
Anopheles gambiae	0	0	0	0	0
Anopheles maculatus	6366	1859	2411	2284	74
Anopheles melas	5081	3080	2181	2001	350
Anopheles merus	590	220	422	326	114
Anopheles minimus	19	7	14	8	4
Anopheles quadriannulatus	238	122	163	130	38
Anopheles sinensis	336	196	218	190	10
Anopheles sinensis (Chinese)	158	166	78	60	14
Anopheles stephensi	277	106	182	134	64
Anopheles stephensi (Indian)	201	69	155	124	43

[7] CAMSA: comparative analysis and merging of scaffold assemblies

Robert M. Waterhouse, Sergey Aganezov, Livio Ruzzante, Maarten J.M.F. Reijnders, Max A. Alekseyev

The CAMSA tool automates the process of comparing and merging scaffold assemblies produced by alternative methods as well as providing interactive visualisations that enable detailed manual inspections of the scaffold adjacency agreements and conflicts identified during the merging process (Aganezov and Alekseyev 2017). CAMSA allows working with both oriented and (partially) un-oriented scaffold assemblies under the same unifying framework, thus greatly simplifying the downstream analysis process when working with data produced by both computational and wet-lab based methods. CAMSA (version 1.1.0b14, https://github.com/compbiol/CAMSA) was applied to the predicted adjacencies from each of the three synteny-based methods to produce three consensus sets for each of the 20 anopheline assemblies: conservative three-way consensus adjacency sets, two-way consensus adjacency sets with no third-method conflicts, and liberal union sets of all non-conflicting adjacencies. Pre-filtering of the predicted adjacencies first removed any pairs of scaffolds where one or both remained un-oriented (i.e., semi-un-oriented assembly pairs were removed). Thus common adjacencies must agree both at the level of being predicted neighbours and their relative orientations. Conflicting adjacencies occur when one or both scaffolds in a pair predicted by one method are predicted to be paired with a different scaffold (or the same scaffold but the opposite orientation) by another method. The remaining unique and non-conflicting adjacencies from each method formed part of the liberal union sets.

Adjacencies in three-way and two-way agreement in the resulting CAMSA-produced consensus sets (**Table S5**) were used to build the synteny-improved assemblies and compute scaffold N50 values and counts before and after merging. As the synteny-based methods rely on orthologous anchors as their input data they cannot predict adjacencies for scaffolds with no annotated orthologous genes, thus N50 values and counts were computed based only on scaffolds with annotated orthologues (**Fig. 2, main text; Figures S7 and S8**). Linear regressions plotted with 95% confidence intervals computed with the geom_smooth() function from the R package ggplot2, specifying the 'lm' method.

Table S5. Synteny-based adjacency agreements

Counts of input (All) and filtered (Use) adjacencies from three synteny-based methods and their agreements or conflicts, reported two-way agreements are required not to conflict with the third method.

Species	ADSEQ		GOS-ASM		ORTHOSTITCH		3-Way Agreement	2-Way Agreement	ADSEQ & GOS-ASM	GOS-ASM & ORTHOSTITCH	ADSEQ & ORTHOSTITCH
	All	Use	All	Use	All	Use					
Anopheles albimanus	1	1	2	2	4	4	0	1	0	1	0
Anopheles arabiensis	48	48	11	11	33	29	2	19	3	0	16
Anopheles atroparvus	42	42	6	6	29	25	1	14	1	0	13
Anopheles christyi	3220	3220	1176	1176	2031	1937	474	1041	238	17	786
Anopheles coluzzii	199	199	71	71	134	123	27	54	8	1	45
Anopheles culicifacies	3594	3594	2055	2055	1821	1742	659	910	494	29	387
Anopheles darlingi	678	678	290	290	684	640	102	281	31	23	227
Anopheles dirus	63	63	19	19	42	39	9	27	5	0	22
Anopheles epiroticus	608	608	143	143	471	456	74	327	28	3	296
Anopheles farauti	177	177	75	75	119	116	43	62	12	2	48
Anopheles funestus	331	331	100	100	211	208	47	171	32	2	137
Anopheles gambiae	0	0	0	0	0	0	0	0	0	0	0
Anopheles maculatus	6366	6366	1859	1859	2411	2312	377	1076	401	26	649
Anopheles melas	5081	5081	3080	3080	2181	2116	773	1075	696	36	343
Anopheles merus	590	590	220	220	422	413	118	254	35	9	210
Anopheles minimus	19	19	7	7	14	14	3	9	3	0	6
Anopheles quadriannulatus	238	238	122	122	163	148	49	81	23	2	56
Anopheles sinensis	336	335	196	196	218	204	30	90	43	7	40
Anopheles sinensis (Chinese)	158	158	166	166	78	77	27	65	45	5	15
Anopheles stephensi	277	277	106	106	182	177	53	124	24	3	97
Anopheles stephensi (Indian)	201	201	69	69	155	144	40	90	12	2	76

A

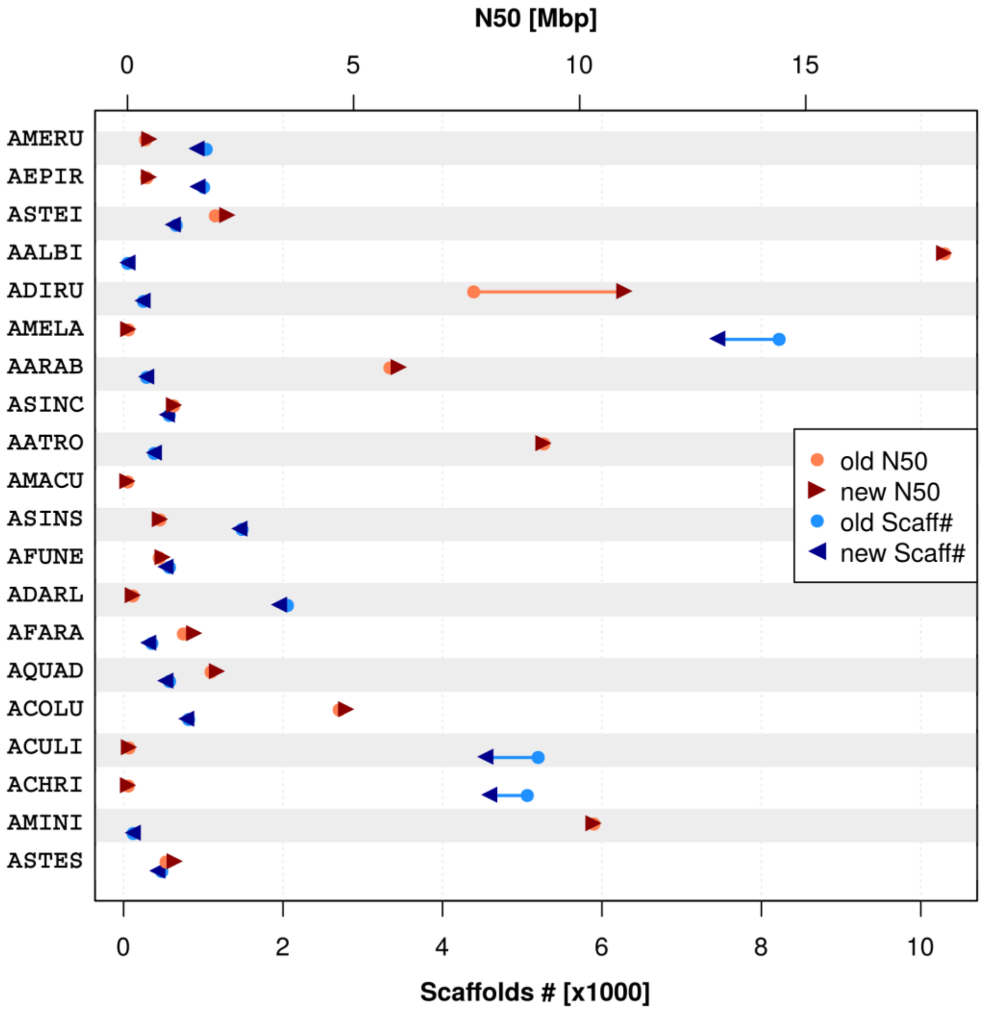

B

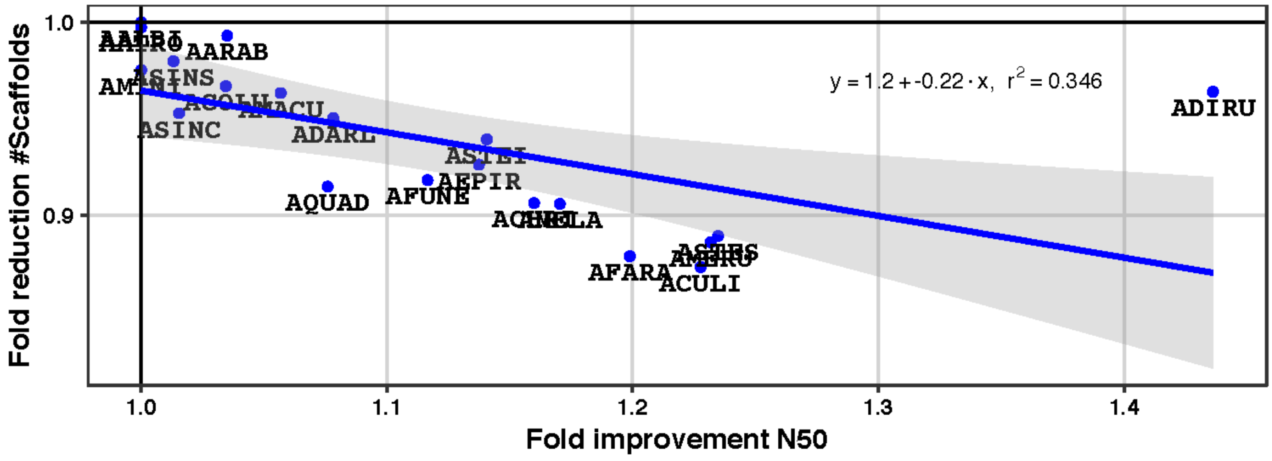

Figure S7. Assembly improvements based on conservative set synteny predictions
For details see Fig. 2, main text.

A

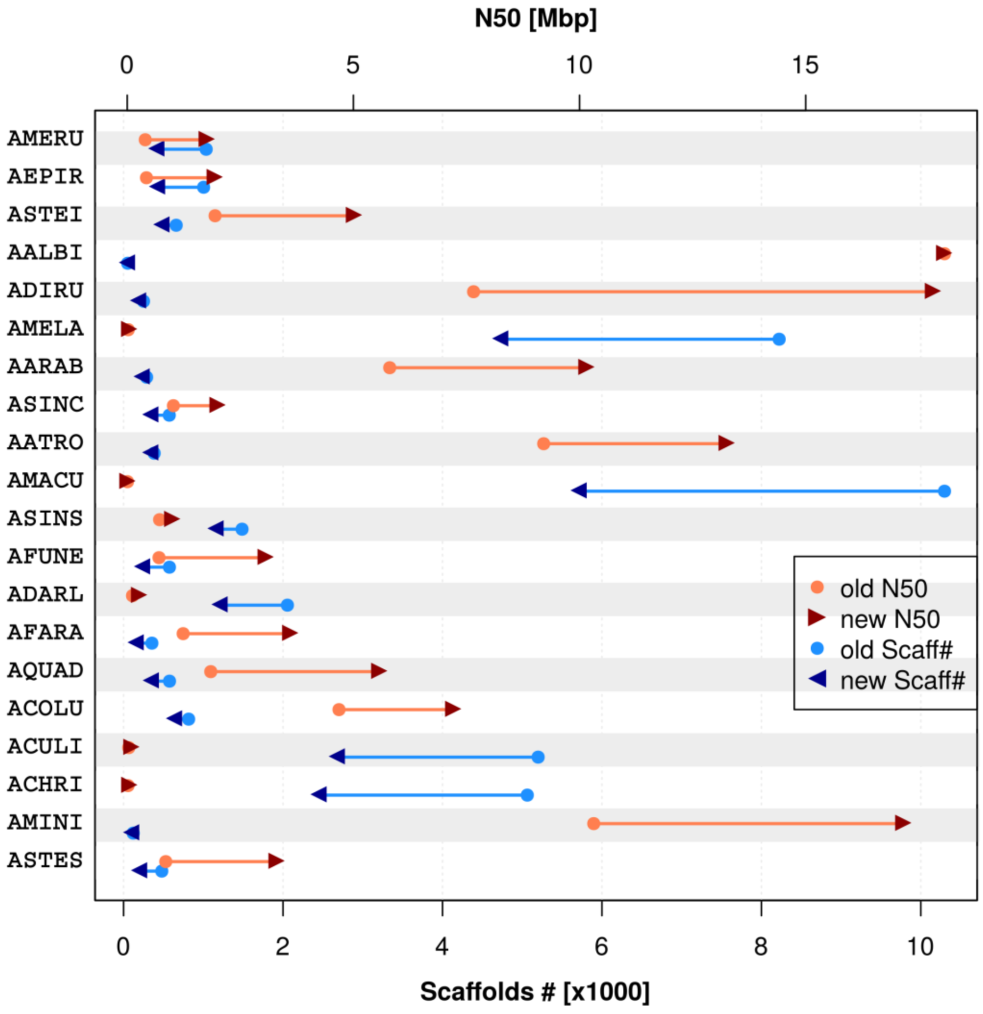

B

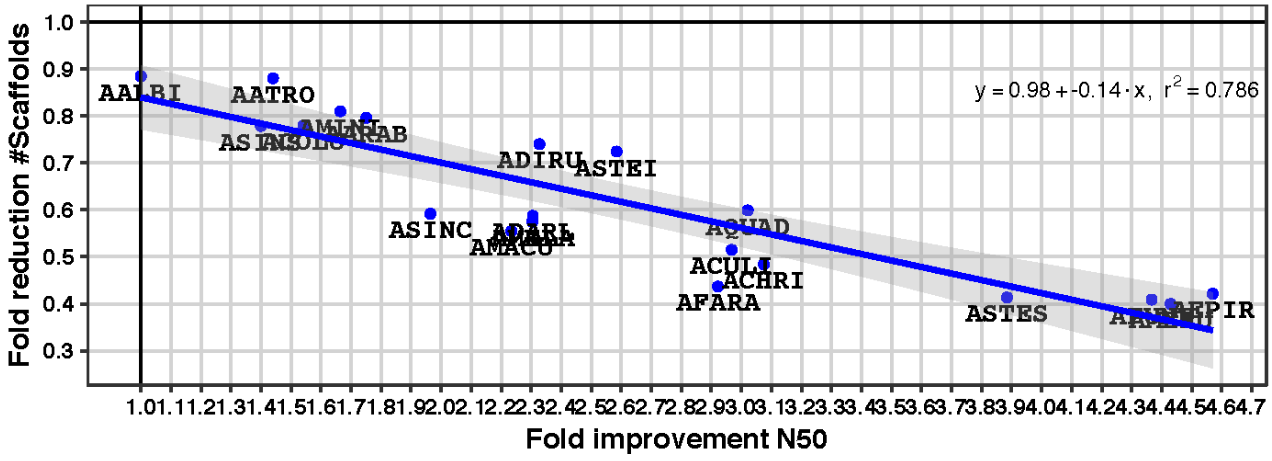

Figure S8. Assembly improvements based on liberal union set synteny predictions

For details see Fig. 2, main text.

Synteny-based method comparisons

Comparing the CAMSA-produced two-way consensus sets with the input adjacencies from each of the three methods quantified agreements (**Table S5**) as well as conflicting and unique adjacencies predicted by each method for each assembly (**Fig. 3, main text; Figure S9**). A total of 29’418 distinct scaffold adjacencies were identified from the combined results of all 42’923 predictions from the three methods. These were classified according to whether they were in three-way agreement, in two-way agreement with no third-method conflict, in two-way agreement but with conflict(s), unique to an individual method with no conflict(s) with the other methods, or unique to an individual method but with conflict(s).

Comparing all 42’923 predictions identified 29’418 distinct scaffold adjacencies, 36% of which were supported by at least two methods. Overall, 10% of the distinct adjacencies were predicted by all three methods, and a further 26% were predicted by two methods but this was reduced to 20% when adjacencies that conflicted with the third method were removed. These 8’878 supported predictions were used to build the two-way consensus sets of scaffold adjacencies for synteny-based assembly improvements presented in **Fig. 2**. Main text **Fig. 3B** shows the overlaps amongst the three methods, plotted as an area-proportional Euler diagram with EULERAPE v3.0.0 (Micallef and Rodgers 2014). Adjacencies in three-way agreement made up 30% of GOS-ASM and 27% of ORTHOSTITCH predictions, and 13% of ADSEQ predictions (as there were about double the number of ADSEQ predictions compared with the other two methods). The much larger total number of ADSEQ predictions resulted in a higher proportion of unique adjacencies (54%) compared with GOS-ASM (35%) and ORTHOSTITCH (31%). Pairwise method comparisons: ADSEQ supported 61% of GOS-ASM and 65% of ORTHOSTITCH predictions; ORTHOSTITCH supported 32% of ADSEQ and 34% of GOS-ASM adjacencies; and GOS-ASM supported 27% of ADSEQ and 30% of ORTHOSTITCH predictions.

Considering only the liberal union sets of all non-conflicting adjacencies, the adjacencies in three-way agreement made up 16.5% of the total, 45.6% of GOS-ASM, 39.1% of ORTHOSTITCH, and 18.6% of ADSEQ predictions (**Fig. 3B, main text**). From the two-way consensus adjacency sets with no third-method conflicts, three-way consensus adjacencies made up 32.8% of the total, 53.8% of GOS-ASM, 44.4% of ORTHOSTITCH, and 33.4% of ADSEQ predictions (**Fig. 3B, main text**). These two-way consensus adjacencies that were employed to build the new superscaffolded assemblies were therefore supported by ADSEQ (98.1%), and/or ORTHOSTITCH (73.7%), and/or GOS-ASM (60.9%), with a third being supported by all three methods. Thus, comparing the results from the three methods and employing a two-way agreement with no third-method conflict filter improved the overall level of three-way agreement from a tenth to a third.

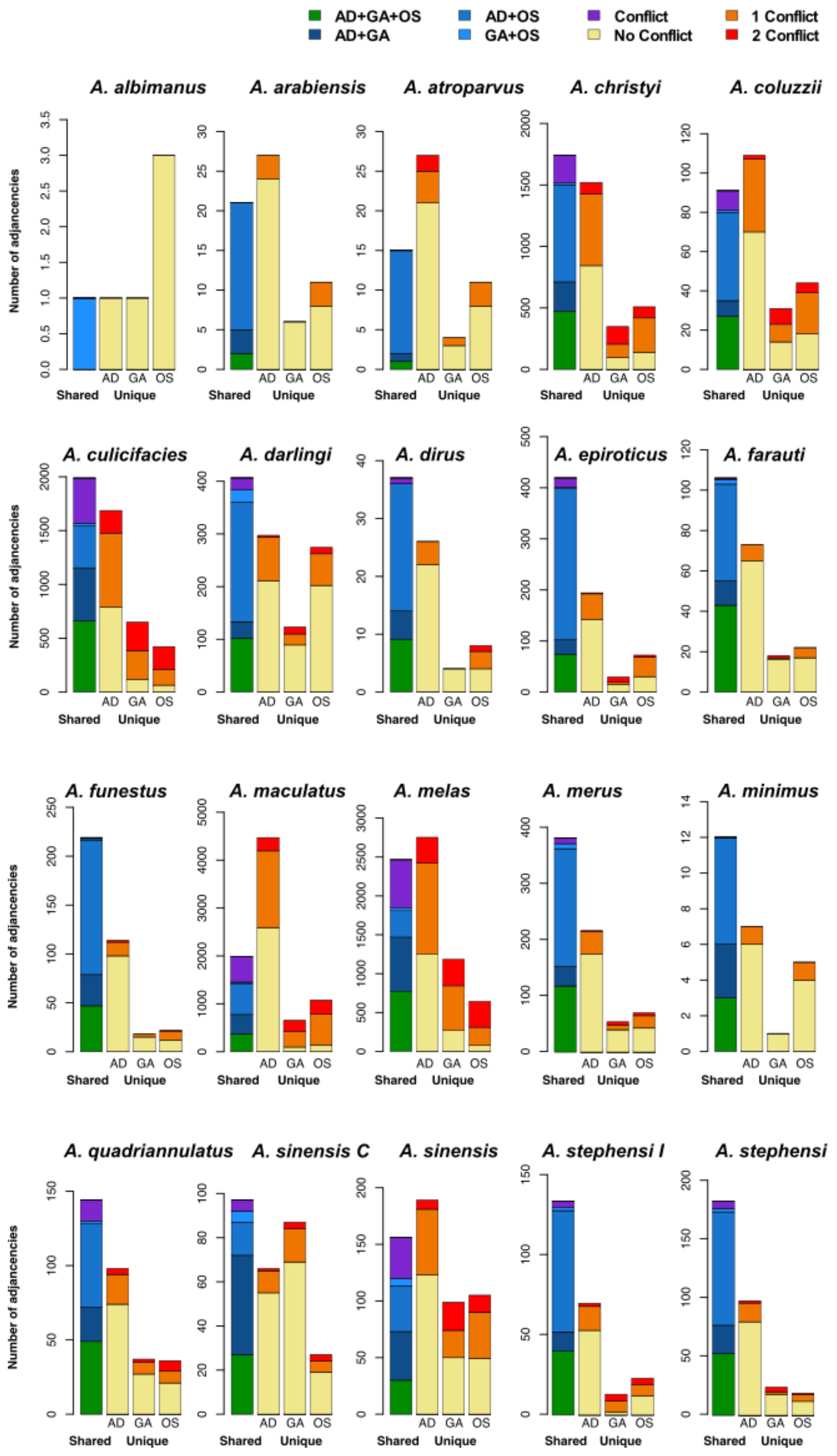

Figure S9. Comparisons of adjacency results from three synteny-based methods

Comparisons of synteny-based scaffold adjacency predictions from ADSEQ (AD), GOS-ASM (GA), and ORTHOSTITCH (OS). Bar charts show counts of predicted adjacencies (pairs of neighbouring scaffolds) that are shared amongst all three methods (green), or two methods without (blues) and with (purple) third method conflicts, or that are unique to a single method and do not conflict (yellow) or do conflict with predictions from one (orange) or both (red) of the other methods. Note variable maxima for y-axes.

Examining the results from each individual assembly (selected assemblies shown **Fig. 3C, main text**; all assemblies shown in **Figures S9 and S10**), showed generally good agreement for at least eight of the assemblies (more than 48% of distinct adjacencies were found to be in at least two-way agreement with no third-method conflict), with *A. funestus* achieving the highest consistency at 58%. Some of the most fragmented input assemblies produced the some of the largest sets of distinct adjacency predictions but the agreement amongst these predictions was generally lower than the other assemblies, e.g. *A. maculatus* with 8'179 distinct adjacencies of which only of which only 18% showed at least two-way agreement with no conflicts (**Figure S10**). *A. albimanus* showed a very low level of agreement (16.7%), but this is primarily because of the very few predicted adjacencies: just six distinct adjacencies with only one being shared between two of the methods.

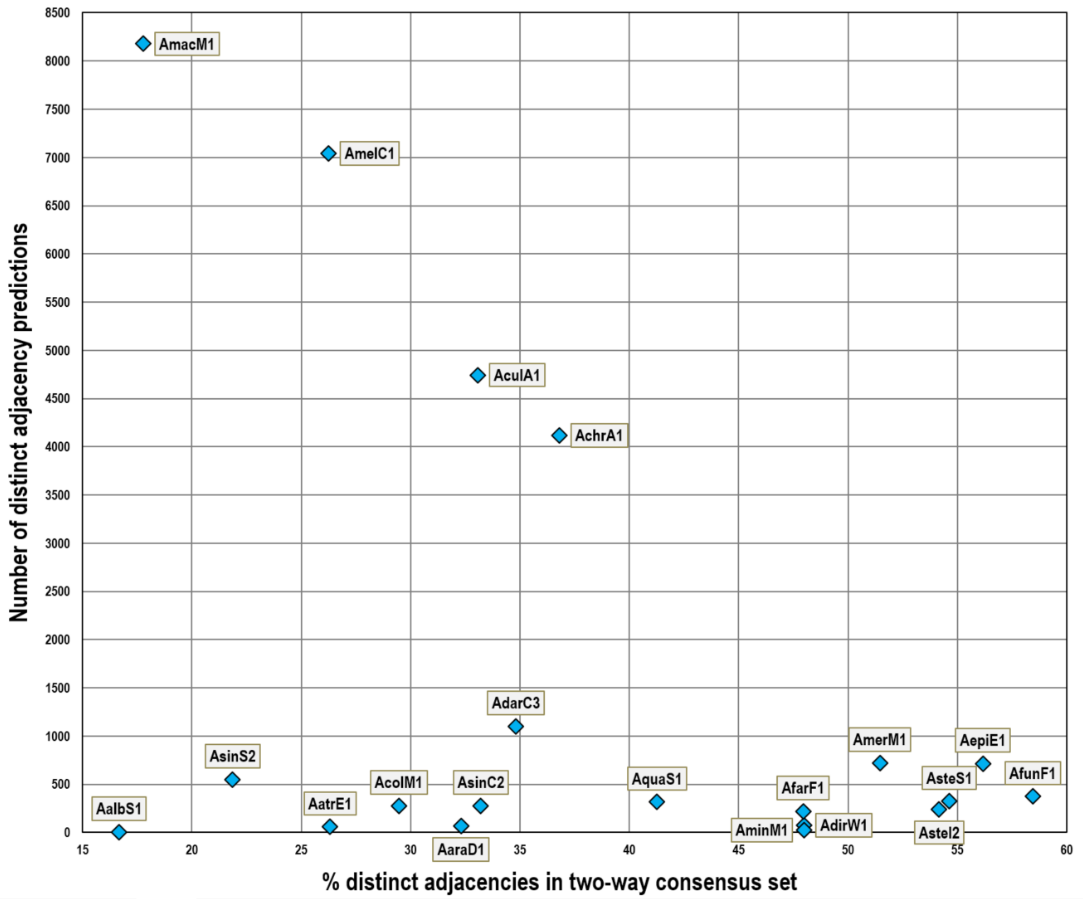

Figure S10. Proportions of synteny-based adjacencies in agreement for each assembly
Comparisons of the number of distinct adjacencies and the proportion of which were common to at least two methods with no third method conflict. Two-way consensus adjacencies made up 48% or more of the distinct predictions for eight assemblies, while some of the most fragmented assemblies with the most predicted adjacencies showed lower levels of agreement. For AalbS1 (*Anopheles albimanus*), only one of the six distinct adjacency predictions was in the two-way consensus set. See **Table S3** for the species that corresponds to each assembly identifier.

[8] Physical mapping data from six anophelines

Jiyoung Lee, Phillip George, Maryam Kamali, Ashley Peery, Maria V. Sharakhova, Maria F. Unger, Igor V. Sharakhov

Methods of chromosomal mapping of scaffolds (Sharakhova et al. 2019; Artemov et al. 2018b) are detailed for *A. albimanus* (Artemov et al. 2017), *A. atroparvus* (Artemov et al. 2015; Neafsey et al. 2015; Artemov et al. 2018a), *A. sinensis* Chinese strain (Wei et al. 2017), *A. stephensi* SDA-500 strain (Neafsey et al. 2015), and *A. stephensi* Indian strain (Jiang et al. 2014). *A. stephensi* mapping added to existing mapping data (Sharakhova et al. 2006, 2010), and *A. funestus* mapping built on previous results (Sharakhov et al. 2002, 2004; Xia et al. 2010) to further develop the physical map as described in detail below. Counts of mapped scaffolds and the resulting scaffold adjacencies, i.e. pairs of neighbouring mapped scaffolds, are summarised in **Table S6** and the complete 'frozen' datasets of the physically mapped scaffolds for each of the six assemblies are presented in **Additional File 4** (final reconciled physical mapping data are presented in **Additional File 5**).

Table S6. Physically mapped scaffolds from six anophelines
Counts of physically mapped scaffolds and adjacencies available for six of the anophelines.

Species	Number of Mapped Scaffolds	Usable Scaffold Pair Adjacencies	Reference(s)
Anopheles albimanus	31	31	(Artemov et al. 2017)
Anopheles atroparvus	46	31	(Artemov et al. 2015; Neafsey et al. 2015; Artemov et al. 2018a)
Anopheles funestus	202	85	(Sharakhov et al. 2002, 2004; Xia et al. 2010; Neafsey et al. 2015) & this study
Anopheles sinensis (Chinese)	52	20	(Wei et al. 2017)
Anopheles stephensi	99	3	(Neafsey et al. 2015)
Anopheles stephensi (Indian)	118	6	(Jiang et al. 2014) & this study

Mosquito strain and ovary preservation:

The FUMOZ strain of *A. funestus* was maintained in the insectary of the Eck Institute, the University of Notre Dame USA. The strain was originally colonized from the Matolo Province of Mozambique, and deposited at the Malaria Research and Reference Reagent Resource (MR4) at the Biodefense and Emerging Infections Research Resources Repository (BEI) under catalogue number MRA-1027. Mosquitoes were raised in a growth chamber at 27°C, with a 12-hour cycle of light and darkness. Approximately 20-21 hours post-blood feeding, ovaries of adult females were pulled out and fixed in Carnoy's solution (3 : 1 ethanol : glacial acetic acid by volume). Ovaries were preserved in fixative solution from 24 h up to 1 month at -20°C.

Chromosome preparation:

Isolated ovaries were bathed in a drop of 50% propionic acid for 5 minutes and squashed as previously described (Sharakhova et al. 2014). The quality of the preparation was assessed with an Olympus CX41 phase contrast microscope (Olympus America Inc., Melville, NY). High-quality chromosome preparations were then flash frozen in liquid nitrogen and immediately placed in cold 50% ethanol. After that, preparations were dehydrated in an ethanol series (50%, 70%, 90%, and 100%) and air-dried. Unstained chromosomes were observed using an Olympus BX41 phase contrast microscope with attached CCD camera Qcolor5 (Olympus America Inc., Melville, NY).

Probe preparation and fluorescence *in situ* hybridization:

Gene-specific primers were designed to amplify unique exon sequences from the beginning and end of each scaffold using the primer-BLAST program (Ye et al. 2012) available at NCBI (http://www.ncbi.nlm.nih.gov/tools/primer-blast/). The primer design was based on gene annotations from the AfunF1 genome assembly available at VECTORBASE (https://www.vectorbase.org/organisms/anopheles-funestus/fumoz/afunf1) (Giraldo-Calderón et al. 2015). PCR was performed using 2X Immomix DNA polymerase (Bioline USA Inc., MA, USA) and a standard Immomix amplification protocol. Amplified fragments were labelled with fluorescein, Cy3 or Cy5 dyes (GE Health Care, UK Ltd, Buckinghamshire, UK and Enzo Biochem, Enzo Life Sciences Inc., Farmingdale, NY) using a Random Primers DNA Labelling System (Invitrogen, Carlsbad, CA, USA). By combining different dyes in one reaction, we labelled and used up to four probes corresponding to two genomic scaffolds in the same FISH experiment. FISH was performed according to the previously described standard protocol (Sharakhova et al. 2014). DNA probes were hybridized to the chromosomes at 39°C for 10-15 hours in a hybridization solution (50% Formamide; 10% Sodium Dextran sulfate, 0.1% Tween 20 in 2XSSC, pH 7.4). Chromosome preparations were washed in 0.2X SSC (Saline-Sodium Citrate: 0.03M Sodium Chloride, 0.003M Sodium Citrate) and counterstained with DAPI in ProLong Gold Antifade Mountant (Thermo Fisher Scientific Inc., USA).

Linking Illumina scaffolds with PacBio contigs and PacBio merged scaffolds:

Illumina scaffolds of the *A. funestus* Anop_fune_FUMOZ_V1 assembly were downloaded from GENBANK (https://www.ncbi.nlm.nih.gov/assembly/GCA_000349085.1). PacBio contigs which were longer than one million bp were aligned to the Illumina scaffolds using BLASTN 2.2.31+ (http://blast.ncbi.nlm.nih.gov/Blast.cgi) with default settings. The PacBio assembly was generated with approximately 70X of PacBio sequencing data and polished by Quiver (see section below on 'Building the PacBio-based *Anopheles funestus* assembly'). To see the reverse alignment relationships, we used Illumina scaffolds as query sequences and align them to PacBio merged scaffolds with the standalone BLASTN 2.2.30+ program installed on a server, and we built BLAST databases. The PacBio merged

scaffolds were obtained by merging PacBio contigs with the Illumina assembly using METASSEMBLER (Wences and Schatz 2015) and then by scaffolding with SSPACE (Boetzer et al. 2011) using available Illumina sequencing data. The BLASTN was performed with the 97% identity and 1e-50 e-value thresholds. Illumina and PacBio merged scaffolds longer than 0.2 million bps were chosen for FISH (**Figure S11**). CIRCOLETTO was used to visualize sequence similarity between linked Illumina scaffolds with merged PacBio scaffolds, their order and orientation (Darzentas 2010). Illumina scaffolds were ordered and oriented within large PacBio contigs and merged PacBio scaffolds, and the resulted arrangements were anchored to chromosomes by FISH as described above.

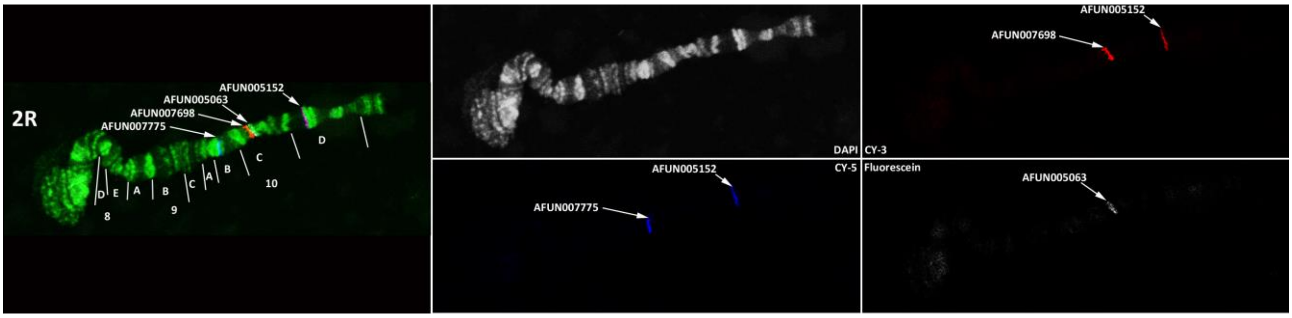

Figure S11. Fluorescence *in situ* hybridization (FISH) mapping in *Anopheles funestus*.
Multicolour FISH of four DNA probes designed based on gene sequences. Polytene chromosomes are from ovarian nurse cells of *A. funestus*.

Chromosome mapping:

Illumina scaffolds and merged Illumina-PacBio arrangements were anchored to chromosomes by several different ways. (1) Scaffolds without adjacency and orientation were placed on chromosomes with only one FISH probe. (2) Oriented scaffolds without adjacency were placed on chromosomes with at least two FISH probes, but they did not have any neighbours. (3) Scaffolds with adjacency but without orientation consisted of two or several neighbouring scaffolds mapped with one FISH probe each. Alternatively, several Illumina scaffolds were predicted to be adjacent within a PacBio contig or PacBio-merged scaffolds by BLAST but the whole assembly was anchored to chromosome by only one FISH probe. (4) Ordered and oriented scaffolds were placed on chromosomes by multiple FISH probes (**Figure S11**) or their adjacency and orientation were inferred from the alignment to a mapped and oriented PacBio contigs or PacBio-merged scaffolds. The resulting physical genome map for *A. funestus* includes 202 AfunF1 scaffolds (**Table S7**).

Table S7. Physically mapped *Anopheles funestus* scaffolds on the cytogenetic map

Chromosomal locations and orientation (if determined) of AfunF1 genomic scaffolds on the *Anopheles funestus* cytogenetic map from 126 previously FISH-mapped (Sharakhov et al. 2002, 2004; Xia et al. 2010) and 66 newly FISH-mapped DNA markers. Note that these mappings incorporate additions and corrections that were made during the reconciliation process and thus there are some differences with the 'input' physical mapping data presented as part of **Additional File 4**.

AfunF1 scaffolds	Scaffold orientation	Chromosome region	Scaffold size	Method of placement to the map
KB668763	-	X:1A	305818	PACBIO OVERLAP
KB669058	+	X:1AB	2367365	MAPPED
KB668322	-	X:1B	615127	MAPPED
KB668245	-	X:1C	629234	MAPPED
KB669125	-	X:1C	833292	MAPPED
KB669181	-	X:1C	789512	PACBIO OVERLAP
KB668600	-	X:1D	671960	MAPPED
KB668720	+	X:1D	419310	PACBIO OVERLAP
KB668844	+	X:1D	215519	PACBIO OVERLAP
KB668852	+	X:1D	216557	MAPPED
KB668755	+	X:2A	379841	PACBIO OVERLAP
KB668367	?	X:2B	636359	MAPPED
KB668936	+	X:2BC	1176300	MAPPED
KB669143	-	X:2C	12834	PACBIO OVERLAP
KB669145	-	X:3A	12715	PACBIO OVERLAP
KB668668	-	X:3AB	583467	MAPPED
KB669003	-	X:3CD	1206901	MAPPED
KB668797	+	X:3D	250364	PACBIO OVERLAP
KB668522	+	X:4A	703988	MAPPED
KB669029	+	X:4A	88731	PACBIO OVERLAP
KB669078	-	X:4AB	51937	PACBIO OVERLAP
KB668688	?	X:5C	429614	MAPPED
KB668389	?	X:5C	547300	PACBIO OVERLAP
KB668765	?	X:6	305606	PACBIO OVERLAP
KB668660	?	X:6	504041	PACBIO OVERLAP
KB668760	?	X:6	504041	MAPPED
KB669536	?	X:6	625123	MAPPED
KB668728	?	2R:7A	333164	MAPPED
KB668825	?	2R:7BC	1174813	MAPPED
KB668221	+	2R:8AE	3832769	MAPPED
KB668954	+	2R:9A	66343	PACBIO OVERLAP
KB669004	+	2R:9A	57566	PACBIO OVERLAP
KB669169	+	2R:9A-10B	1771395	MAPPED
KB668759	-	2R:10BC	1353732	MAPPED
KB668737	+	2R:10C	1261231	MAPPED
KB668555	-	2R:10D-11A	1493947	MAPPED
KB668845	?	2R:11B	188083	MAPPED
KB668753	-	2R:11C	343795	PACBIO OVERLAP
KB668871	+	2R:11C	218729	PACBIO OVERLAP
KB668467	-	2R:12A	563073	MAPPED
KB669369	+	2R:12B	804489	MAPPED
KB668822	?	2R:12B	194798	MAPPED
KB668793	?	2R:12C	254162	MAPPED

KB668745	?	2R:12C	346721	PACBIO OVERLAP
KB668672	-	2R:12D	584724	MAPPED
KB668775	-	2R:12D	355205	PACBIO OVERLAP
KB668785	-	2R:12E	408060	MAPPED
KB669081	-	2R:12E-13A	1051734	MAPPED
KB668706	?	2R:13A	572386	MAPPED
KB668715	?	2R:13B	364622	MAPPED
KB668766	?	2R:13C	300363	MAPPED
KB668757	?	2R:13C	303086	PACBIO OVERLAP
KB668411	?	2R:13CD	533316	PACBIO OVERLAP
KB668679	?	2R:13D	436776	MAPPED
KB668478	?	2R:14B	598359	MAPPED
KB669525	?	2R:14C	624454	MAPPED
KB668835	-	2R:14D	178699	MAPPED
KB669358	-	2R:15B	786284	MAPPED
KB668911	?	2R:15C	97490	MAPPED
KB669547	?	2R:15E	618077	MAPPED
KB668914	+	2R:15E-16A	1038133	MAPPED
KB668837	?	2R:16A	1126662	MAPPED
KB669192	?	2R:16B	922711	MAPPED
KB668748	?	2R:16C	1398093	MAPPED
KB668670	-	2R:17AB	1428115	MAPPED
KB668947	+	2R:17C-18A	2413216	MAPPED
KB668742	-	2R:18A	317854	PACBIO OVERLAP
KB668234	+	2R:18AB	602066	MAPPED
KB668734	-	2R:18C	433875	MAPPED
KB668836	+	2R:18CD	2772343	MAPPED
KB668289	-	2R:18D	644640	PACBIO OVERLAP
KB669247	+	2R:19A	782462	PACBIO OVERLAP
KB668866	-	2R:19B	140173	PACBIO OVERLAP
KB669114	-	2R:19C	878476	MAPPED
KB668942	-	2R:19C	101067	PACBIO OVERLAP
KB668870	-	2R:19CD	1070199	MAPPED
KB668694	-	2R:19DE	522677	PACBIO OVERLAP
KB669281	?	2L:20BC	897800	MAPPED
KB669092	?	2L:20C	887634	MAPPED
KB669070	?	2L:20D	943178	MAPPED
KB668589	+	2L:21BC	555648	PACBIO OVERLAP
KB668770	+	2L:21C	1246493	MAPPED
KB668781	-	2L:21CD	1311501	MAPPED
KB668222	-	2L:21D-22A	1876834	MAPPED
KB668692	-	2L:22AC	1835194	MAPPED
KB668872	-	2L:22C	248811	MAPPED
KB669502	?	2L:22D	1948688	MAPPED
KB668882	+	2L:23A	121550	MAPPED
KB668803	+	2L:24AB	1311425	MAPPED
KB669036	+	2L:24B	850007	PACBIO OVERLAP
KB668433	-	2L:24B	552028	PACBIO OVERLAP
KB669280	+	2L:24CD	1738428	MAPPED
KB668854	?	2L:26A	204352	PACBIO OVERLAP
KB669047	?	2L:26A	999242	MAPPED
KB668681	+	2L:26C	1609593	MAPPED
KB668702	-	2L:26C	460558	PACBIO OVERLAP
KB668764	-	2L:26CD	327039	MAPPED
KB668693	+	2L:26D	518197	MAPPED
KB668278	-	2L:27A	702492	PACBIO OVERLAP

KB669136	-	2L:27AB	882720	MAPPED
KB668813	?	2L:27C	258639	PACBIO OVERLAP
KB668795	?	2L:27C	281738	MAPPED
KB669214	?	2L:27CD	874018	PACBIO OVERLAP
KB668992	?	2L:27D	953533	MAPPED
KB668751	?	2L:27E	335862	MAPPED
KB668892	?	2L:27E	1102954	PACBIO OVERLAP
KB668725	?	2L:28A	3134932	MAPPED
KB668881	?	2L:28C	976588	MAPPED
KB668378	?	3R:29B	571908	MAPPED
KB669236	?	3R:29C	713413	MAPPED
KB668851	?	3R:29C	164446	PACBIO OVERLAP
KB668683	?	3R:29CD	458860	MAPPED
KB669458	-	3R:29D-30A	679292	MAPPED
KB668792	?	3R:30AC	1431544	MAPPED
KB668633	?	3R:30C	577339	MAPPED
KB669089	?	3R:30C	19591	PACBIO OVERLAP
KB668687	?	3R:30C	589177	PACBIO OVERLAP
KB668808	+	3R:30C	233839	MAPPED
KB668705	+	3R:30CD	400982	PACBIO OVERLAP
KB668695	?	3R:31C	480392	PACBIO OVERLAP
KB668812	?	3R:31CD	217759	MAPPED
KB668644	?	3R:32B	475666	MAPPED
KB669580	?	3R:32B	640366	PACBIO OVERLAP
KB668789	?	3R:33A	318576	MAPPED
KB668661	+	3R:33C	494022	MAPPED
KB668533	?	3R:33C	534943	MAPPED
KB669347	?	3R:33D	864369	MAPPED
KB668723	?	3R:33D	382759	MAPPED
KB668818	?	3R:34A	257953	PACBIO OVERLAP
KB668746	?	3R:34A	323294	MAPPED
KB668750	-	3R:34B	390086	PACBIO OVERLAP
KB668848	-	3R:34BC	1452984	MAPPED
KB668671	?	3R:35B	710502	MAPPED
KB668853	?	3R:35B	306342	MAPPED
KB668790	?	3R:35C	381342	MAPPED
KB668700	-	3R:35CD	478136	PACBIO OVERLAP
KB668684	+	3R:35D	499467	PACBIO OVERLAP
KB669103	-	3R:35DE	1272063	MAPPED
KB669336	+	3R:35E	1014753	MAPPED
KB668456	+	3R:35EF	708050	MAPPED
KB669011	-	3R:35F	39851	MAPPED
KB668709	-	3R:35F	491865	MAPPED
KB668752	-	3R:35F	447341	PACBIO OVERLAP
KB668816	+	3R:35F	290561	MAPPED
KB668880	+	3R:35F	119321	MAPPED
KB669403	+	3R:35F	972476	MAPPED
KB669034	+	3R:35F	31323	PACBIO OVERLAP
KB668756	-	3R:35F	460866	PACBIO OVERLAP
KB668777	-	3R:36A	381111	MAPPED
KB668731	-	3R:36A	405878	MAPPED
KB668779	-	3R:36A	398220	PACBIO OVERLAP
KB668987	-	3R:36AB	46664	PACBIO OVERLAP
KB669414	+	3R:36B	997879	PACBIO OVERLAP
KB668810	+	3R:36B	310469	PACBIO OVERLAP
KB669380	+	3R:36B	770134	PACBIO OVERLAP

KB668858	+	3R:36B	254377	PACBIO OVERLAP
KB668667	+	3R:36B	580684	MAPPED
KB668806	+	3R:36C	292235	MAPPED
KB668874	+	3R:36C	134456	MAPPED
KB668959	+	3R:36D	938304	MAPPED
KB668732	+	3R:36D	346637	MAPPED
KB668999	-	3R:36D	106097	PACBIO OVERLAP
KB669391	-	3R:36DE	2124680	MAPPED
KB669436	-	3R:36E	911299	MAPPED
KB668819	-	3R:36E	222314	PACBIO OVERLAP
KB668744	-	3R:36E	359052	PACBIO OVERLAP
KB669469	+	3R:36F	758275	MAPPED
KB669203	+	3R:36F	774987	PACBIO OVERLAP
KB668970	+	3R:37A	879108	PACBIO OVERLAP
KB668726	+	3R:37AB	1292245	MAPPED
KB668762	-	3R:37C	351750	PACBIO OVERLAP
KB668805	-	3L:38A	229600	MAPPED
KB668859	+	3L:38B	1041161	MAPPED
KB668823	?	3L:38C	194609	MAPPED
KB669325	?	3L:39A	699719	MAPPED
KB668717	?	3L:39A	402095	MAPPED
KB668578	?	3L:39A	505528	MAPPED
KB668676	?	3L:39A	439401	PACBIO OVERLAP
KB668659	?	3L:39B	1545011	MAPPED
KB669014	?	3L:40A	892505	MAPPED
KB668773	?	3L:40A	296798	PACBIO OVERLAP
KB668868	?	3L:40B	141784	MAPPED
KB668918	?	3L:40B	93149	MAPPED
KB668444	?	3L:41A	1547433	MAPPED
KB668754	?	3L:41D	348546	MAPPED
KB668830	?	3L:41D	181046	MAPPED
KB668333	+	3L:42AB	1525199	MAPPED
KB668422	?	3L:42D	532383	MAPPED
KB668500	?	3L:43A	547302	MAPPED
KB668925	?	3L:43B	940405	MAPPED
KB669025	?	3L:44B	910511	MAPPED
KB669264	?	3L:44B	32220	MAPPED
KB669603	?	3L:44C	2248	MAPPED
KB668849	?	3L:44C	159962	MAPPED
KB668784	?	3L:45A	266933	MAPPED
KB668948	-	3L:46B	917728	PACBIO OVERLAP
KB668682	-	3L:46B	470092	MAPPED
KB668714	-	3L:46BC	1305928	MAPPED
KB668703	-	3L:46CD	1311118	MAPPED
KB669207	?	3L:46D	62723	MAPPED
KB668252	?	3L:46D	2000	MAPPED
KB668265	?	3L:46D	1954	MAPPED

As for the comparisons of the synteny-based results, CAMSA was used to compare the two-way consensus sets, as well as the conservative three-way consensus sets and the liberal union sets of all non-conflicting adjacencies, with the physical mapping adjacencies from each of the six assemblies and quantify agreements as well as conflicting and unique adjacencies (**Table S8**).

For *A. albimanus*, the two-way consensus synteny-based predictions produced only a single adjacency, and this was confirmed by the physical mapping data. Five of the 15 two-way consensus synteny-based predictions were confirmed by physical mapping of *A. atroparvus* scaffolds and only one conflict (resolved) was identified (**Fig. 4A, main text**). The mapped scaffolds for the *A. stephensi* assemblies resulted very few adjacencies, the three SDA-500 strain adjacencies were all in conflict with synteny-based predictions, and of the six Indian strain adjacencies three were shared and one was in conflict with the two-way consensus synteny-based predictions. These conflicts were resolved by correcting the orientations of the physically mapped scaffolds, as the probe designs meant that mapping misorientations were possible.

Comparing the 20 *A. sinensis* (Chinese) mapped scaffolds confirmed three of the synteny-based adjacencies, but none of these were in the consensus sets, and identified conflicts with just two of the 92 two-way consensus adjacencies, both of which were resolved as they involved scaffolds that had not been selected for physical mapping. And finally, *A. funestus* presented the most adjacencies from both physical mapping and the synteny-based predictions where 12-17% of the different sets of synteny-based adjacencies were confirmed and just 4-8% were in conflict (**Fig. 4A, main text**). Amongst the 14 physically mapped neighbouring pairs that conflicted with 13 synteny-based adjacencies from the two-way consensus set, five conflicts were resolved because the synteny-based neighbour was short and not used for physical mapping. An additional four conflicts were resolved by switching the orientation of physically mapped scaffolds, which were anchored by only a single FISH probe and therefore their orientations were not confidently determined. All but one of these adjacency conflicts were resolved either because the scaffolds involved had not been selected for physical mapping or because the orientation determined by physical mapping was not confident and was thus inverted.

Table S8. Physical mapping and synteny-based adjacency comparisons

Comparisons of physical mapping and synteny-based adjacencies for six of the anophelines.

Species	Synteny Set	Physical mapping with conflicts	Physical mapping with no conflicts	Common to physical mapping & synteny	Synteny with no conflicts	Synteny with conflicts
Anopheles albimanus	3-way	0	31	0	0	0
	2-way	0	30	1	0	0
	liberal	2	26	3	1	2
	ADSEQ	0	31	0	1	0
	GOS-ASM	1	29	1	0	1
	ORTHOSTITCH	1	27	3	0	1
Anopheles atroparvus	3-way	0	30	1	0	0
	2-way	1	25	5	9	1
	liberal	4	18	9	33	4
	ADSEQ	3	21	7	31	4
	GOS-ASM	2	26	3	1	2
	ORTHOSTITCH	3	23	5	17	3
Anopheles funestus	3-way	3	74	8	37	2
	2-way	14	40	31	174	13
	liberal	19	21	45	272	23
	ADSEQ	20	18	47	258	26
	GOS-ASM	11	62	12	80	8
	ORTHOSTITCH	16	40	29	165	14
Anopheles sinensis (Chinese)	3-way	0	20	0	27	0
	2-way	2	18	0	90	2
	liberal	5	12	3	225	6
	ADSEQ	5	15	0	152	6
	GOS-ASM	5	13	2	159	5
	ORTHOSTITCH	1	18	1	75	1
Anopheles stephensi (SDA-500)	3-way	3	0	0	51	2
	2-way	3	0	0	174	3
	liberal	3	0	0	278	3
	ADSEQ	3	0	0	274	3
	GOS-ASM	3	0	0	104	2
	ORTHOSTITCH	3	0	0	174	3
Anopheles stephensi (Indian)	3-way	0	4	2	38	0
	2-way	1	2	3	124	1
	liberal	1	2	3	184	1
	ADSEQ	2	1	3	195	3
	GOS-ASM	1	3	2	66	1
	ORTHOSTITCH	1	2	3	140	1

[9] RNA sequencing data from 13 anophelines

Robert M. Waterhouse, Matthew W. Hahn, Simo V. Zhang

Transcriptome data from RNA sequencing (RNAseq) experiments can provide additional information about putative scaffold adjacencies when individual transcripts (or paired-end reads) reliably map to scaffold extremities. For example, extensive RNAseq data were applied to the Norway spruce genome to produce 11’528 new scaffolds from 13’811 new edges through RNAseq scaffolding (Nystedt et al. 2013), and transcript-based scaffolding of the Loblolly pine genome linked together 31’231 scaffolds into 9’170 larger scaffolds (Zimin et al. 2014). Although large introns could potentially result in scaffold skipping and introduce large gaps, the *Anopheles* genomes are all relatively small (as shown in **Figure 1, main text**), and long introns are rare: e.g. the best annotated *An. gambiae* has a mean intron length of 1577 bp and only ~1.5% are longer than 20kbp; average of mean lengths, 776 bp; with an average 101 introns per assembly longer than 20Kbp). The presence of highly similar paralogues could also lead to incorrect read mapping that can hinder the correct identification of scaffold-spanning transcripts, but confident adjacencies can be identified by using uniquely-mapping reads with good coverage.

The Annotated Genome Optimization Using Transcriptome Information (AGOUTI) tool (Zhang et al. 2016) employs RNAseq data to identify such adjacencies as well as correcting any fragmented gene models at the ends of scaffolds. AGOUTI identifies pairs of reads that are mapped to different contigs/scaffolds (joining-pairs) and uses only those joining-pairs that are uniquely mapped with a default minimum coverage of five reads. Performance of AGOUTI was previously evaluated by randomly fragmenting the genome of *Caenorhabditis elegans* (N2 strain) with six different levels of fragmentation (Zhang et al. 2016), and compared the results with another RNAseq-based scaffolder, RNAPATH (Mortazavi et al. 2010).

AGOUTI v0.3.3-24-g64c2a76 was applied to 13 anopheline assemblies using genome-mapped paired-end RNAseq data available from VECTORBASE (Giraldo-Calderón et al. 2015) (Release VB-2017-02), including those from the *Anopheles* 16 Genomes Project (Neafsey et al. 2015) and an *A. stephensi* (Indian) male/female study (Jiang et al. 2015). These data were downloaded from VECTORBASE in the form of pre-computed BAM files – RNAseq reads aligned to the assemblies using HISAT2 version 2.0.4 (Kim et al. 2015). All BAM files were sorted by read name (required by AGOUTI), and where more than one BAM file was available for a given assembly they were first merged, both sorting and merging was performed using SAMTOOLS version 0.1.19-44428cd (Li et al. 2009). AGOUTI was run in scaffold mode with default parameters, e.g. for *A. dirus* ‘python2 agouti.py scaffold -assembly anopheles-dirus.fa -bam AdirW1.sorted.bam -gff anopheles-dirus.gff3 -outdir ADIRU’. The numbers of resulting predicted adjacencies ranged from just two for *A. albimanus* to more than 200 *A. sinensis* (SINENSIS) (**Table S9**).

Validation of the AGOUTI-predicted adjacencies was performed using the alternative RNAseq-based approach of RASCAF (Song et al. 2016), GitHub version 10.07.2018, with minimum support for connecting two contigs of five and the coordinate-sorted alignment BAM files. RASCAF consistently predicted more adjacencies than AGOUTI and full support for the AGOUTI-predicted adjacencies ranged from 2/2 for *An. albimanus* to just 5/39 for *An. atroparvus* (**Table S9**). Adjacencies predicted by both methods were given priority during reconciliation with the scaffold adjacencies from synteny and physical mapping data.

Table S9. AGOUTI-based scaffold adjacencies from 13 anophelines
Assemblies with paired-end RNAseq BAM files from VECTORBASE used to run AGOUTI and RASCAF to predict scaffold adjacencies from transcriptome data.

Species	Assembly	Gene set	RNAseq Dataset(s)	Adjacencies
Anopheles albimanus	AalbS1	AalbS1.4	SRS259216_Generic_RNAseq_for_gene_prediction_AalbS1	2 [2]
Anopheles arabiensis	AaraD1	AaraD1.5	SRS259215_Generic_RNAseq_for_gene_prediction_AaraD1	34 [12]
Anopheles atroparvus	AatrE1	AatrE1.4	SRP021065_Generic_RNAseq_for_gene_prediction_AatrE1	39 [5]
Anopheles dirus	AdirW1	AdirW1.4	SRP021066_Generic_RNAseq_for_gene_prediction_AdirW1	21 [7]
Anopheles epiroticus	AepiE1	AepiE1.4	SRP043018_Generic_RNAseq_for_gene_prediction_AepiE1	27 [23]
Anopheles farauti	AfarF1	AfarF1.2	SRP020562_merged_AfarF1	48 [27]
Anopheles funestus	AfunF1	AfunF1.5	SRP021067_Generic_RNAseq_for_gene_prediction_AfunF1	94 [58]
Anopheles merus	AmerM1	AmerM1.2	SRP020545_merged_AmerM1	159 [94]
Anopheles minimus	AminM1	AminM1.4	SRP021068_Generic_RNAseq_for_gene_prediction_AminM1	16 [7]
Anopheles quadriannulatus	AquaS1	AquaS1.5	SRS259214_Generic_RNAseq_for_gene_prediction_AquaS1	96 [56]
Anopheles sinensis	AsinS2	AsinS2.2	SRP035663_Generic_RNAseq_for_gene_prediction_AsinS2	210 [120]
Anopheles stephensi	AsteS1	AsteS1.4	SRP020546_Generic_RNAseq_for_gene_prediction_(AGC)_AsteS1<br>SRP052094-SRP052164_MSQ43_cell_line_AsteS1	99 [45]
Anopheles stephensi (Indian)	AsteI2	AsteI2.3	SRS866621-SRS866625_Male_AsteI2<br>SRS866626-SRS866630_Female_AsteI2	198 [68]

As for the comparisons of the physical mapping results with the synteny-based results, CAMSA was used to compare the two-way consensus sets, as well as the conservative three-way consensus sets and the liberal union sets of all non-conflicting adjacencies, with the AGOUTI-based adjacencies from each of the 13 assemblies and quantify agreements as well as conflicting and unique adjacencies (**Table S10**). The AGOUTI-based scaffold adjacencies supported up to 17-20% of two-way consensus synteny-based adjacencies in some species, with generally few conflicts but up to 11% and 14% conflicting for *A. stephensi* (Indian) and *A. sinensis* (SINENSIS), respectively, which had the most AGOUTI-based scaffold adjacencies. Across all 13 assemblies, 18% of AGOUTI-based scaffold adjacencies supported the two-way consensus synteny-based adjacencies, with only 7% in conflict and 75% were unique to the AGOUTI sets.

Nearly 200 AGOUTI-based scaffold adjacencies for *A. stephensi* (Indian) confirmed only eight and conflicted with 14 of the two-way consensus set adjacencies (**Fig. 4B, main text**). In contrast, about half as many AGOUTI-based scaffold adjacencies each for *A. stephensi* (SDA-500) and *A. funestus* confirmed four to five times as many two-way consensus set adjacencies and conflicted with only five and six, respectively. Notably, 68% of the AGOUTI-based scaffold adjacencies that produced conflicts with the two-way consensus set adjacencies comprised scaffolds with no annotated orthologues. Such non-annotated scaffolds were also numerous amongst the adjacencies that were unique to AGOUTI where for 66% either one or both scaffolds had no annotated orthologues.

Table S10. AGOUTI and synteny-based adjacency comparisons

Comparisons of AGOUTI and synteny-based adjacencies for 13 of the anophelines.

Species	Synteny Set	AGOUTI with conflicts	AGOUTI with no conflicts	Common to AGOUTI & synteny	Synteny with no conflicts	Synteny with conflicts
Anopheles albimanus	3-way	0	2	0	1	0
	2-way	0	2	0	6	0
	liberal	0	2	0	1	0
	ADSEQ	0	2	0	2	0
	GOS-ASM	0	2	0	4	0
	ORTHOSTITCH	0	34	0	2	0
Anopheles arabiensis	3-way	2	32	0	19	2
	2-way	5	27	2	50	7
	liberal	5	28	1	40	7
	ADSEQ	1	32	1	9	1
	GOS-ASM	3	31	0	26	3
	ORTHOSTITCH	0	39	0	1	0
Anopheles atroparvus	3-way	1	37	1	13	1
	2-way	5	32	2	39	5
	liberal	7	30	2	33	7
	ADSEQ	0	39	0	6	0
	GOS-ASM	4	34	1	20	4
	ORTHOSTITCH	0	20	1	8	0
Anopheles dirus	3-way	1	18	2	33	1
	2-way	3	16	2	60	3
	liberal	1	18	2	60	1
	ADSEQ	1	18	2	16	1
	GOS-ASM	2	18	1	36	2
	ORTHOSTITCH	0	25	2	72	0
Anopheles epiroticus	3-way	2	16	9	391	2
	2-way	5	11	11	565	5
	liberal	5	12	10	593	5
	ADSEQ	1	22	4	138	1
	GOS-ASM	4	14	9	443	4
	ORTHOSTITCH	0	45	3	40	0
Anopheles farauti	3-way	4	33	11	90	4
	2-way	6	16	26	167	7
	liberal	5	22	21	150	6
	ADSEQ	1	36	11	63	1
	GOS-ASM	6	32	10	100	6
	ORTHOSTITCH	1	82	11	35	1
Anopheles funestus	3-way	5	49	40	172	6
	2-way	14	29	51	272	17
	liberal	14	27	53	261	17
	ADSEQ	1	72	21	77	2
	GOS-ASM	11	50	33	164	11
	ORTHOSTITCH	3	142	14	101	3
Anopheles merus	3-way	13	103	43	318	11
	2-way	23	68	68	536	20

	liberal	20	76	63	509	18
	ADSEQ	5	131	23	192	5
	GOS-ASM	19	99	41	356	16
	ORTHOSTITCH	0	14	2	1	0
Anopheles minimus	3-way	0	14	2	10	0
	2-way	2	12	2	19	2
	liberal	2	12	2	15	2
	ADSEQ	0	14	2	5	0
	GOS-ASM	0	14	2	12	0
	ORTHOSTITCH	4	86	6	39	4
Anopheles quadriannulatus	3-way	7	69	20	102	7
	2-way	14	54	28	192	15
	liberal	13	56	27	197	14
	ADSEQ	10	69	17	95	10
	GOS-ASM	15	65	16	118	14
	ORTHOSTITCH	7	199	4	20	6
Anopheles sinensis (SINENSIS)	3-way	18	178	14	87	17
	2-way	38	147	25	271	41
	liberal	37	146	27	271	37
	ADSEQ	33	161	16	149	31
	GOS-ASM	25	172	13	167	24
	ORTHOSTITCH	9	188	1	30	9
Anopheles stephensi (Indian)	3-way	14	176	8	106	14
	2-way	23	164	11	153	24
	liberal	23	162	13	164	24
	ADSEQ	11	184	3	55	11
	GOS-ASM	17	174	7	120	17
	ORTHOSTITCH	2	86	11	40	2
Anopheles stephensi (SDA-500)	3-way	6	58	35	137	5
	2-way	12	32	55	216	10
	liberal	9	36	54	214	9
	ADSEQ	8	77	14	86	6
	GOS-ASM	9	52	38	132	7
	ORTHOSTITCH	0	2	0	1	0

[10] Building the PacBio-based *Anopheles funestus* assembly

Paul I. Howell, Sergey Koren, Adam M. Phillippy, Nora J. Besansky, Scott J. Emrich

A new *A. funestus* assembly, AfunF2-IP, was generated using approximately 70X of PacBio sequencing data and polished with QUIVER (PacBio's SMRT Analysis software suite). This was merged with the reference assembly (AfunF1) using METASSEMBLER (Wences and Schatz 2015) to generate a merged assembly. Finally, the merged assembly was scaffolded with SSPACE (Boetzer et al. 2011) using the available Illumina sequencing data. Summary statistics for the reference AfunF1, PacBio only, Illumin+PacBio Merged, and Merged+Scaffolded AfunF2-IP assemblies (using 225 Mbp as a genome size) are presented in **Table S11** and **Figure S12** (to compute contig statistics, the scaffolds were split at three consecutive Ns).

At the contig level the new AfunF2-IP assembly is an improvement over the reference AfunF1, e.g. the number of contigs is reduced from 9'880 to 4'170 and the NG50 increases from 47 Kbp to 194 Kbp. However, longer-range scaffolding of these contigs unfortunately failed to produce a better quality scaffold-level assembly. In terms of gene content, analysis with 2'799 dipteran Benchmarking Universal Single-Copy Orthologues (BUSCOs) (Simão et al. 2015; Waterhouse et al. 2018, 2019) indicates that despite the better contigs fewer BUSCOs are found as complete genes in the AfunF2-IP assembly (**Table S11**). For comparison, the new chromosomal-level assembly for *A. funestus* (Ghurye et al. 2019a) (AfunF3) achieves slightly lower BUSCO completeness with 96.0% 'complete' (**Table S1**).

The AfunF1 assembly has a very high level of N's, 15.63% compared with just 0.90% for the AfunF2-IP assembly, reflecting how scaffolding improves N50 measures but mainly by joining contigs with stretches of unknown nucleotides (N's). When the scaffolds are artificially de-scaffolded by splitting them at consecutive runs of 3, 300, and 1'000 Ns the new AfunF2-IP assembly is clearly much better (**Figure S12**). The stringent splitting at N>=3 also indicates the greater integrity of the sequence quality of the AfunF2-IP assembly as this does not result in high fragmentation levels as it does for AfunF1 (i.e. from 3'772 scaffolds to 4'186 contigs for AfunF2-IP but from 1'391 scaffolds to 9'878 contigs for AfunF1).

Table S11. Comparisons of *Anopheles funestus* assemblies

Statistics describing the *Anopheles funestus* old reference AfunF1, PacBio only, Illumina+PacBip Merged, and Merged+Scaffolded AfunF2-IP assemblies.

		scaffolds	contigs	BUSCO Scores (% of 2'799 dipteran BUSCOs) Complete[Single-Copy,Duplicated],Fragmented,Missing
**Reference AfunF1**	Count	1,392	9,880	C:98.4%[S:97.9%,D:0.5%],F:1.0%,M:0.6%,
	Total Basepairs	225,223,604	190,015,44	
	NG50	671,960	47,164	
	Maximum	3,832,769	563,645	
**PacBio only**	Count	N/A	18,595	N/A
	Total Basepairs	N/A	445,128,909	
	NG50	N/A	147,143	
	Maximum	N/A	4,813,330	
**Illumina+PacBio Merged**	Count	N/A	4,653	N/A
	Total Basepairs	N/A	260,811,249	
	NG50	N/A	146,657	
	Maximum	N/A	3,313,857	
**Merged+Scaffolded AfunF2-IP**	Count	3,773	4,170	C:92.5%[S:85.8%,D:6.7%],F:4.7%,M:2.8%
	Total Basepairs	263,192,532	260,811,631	
	NG50	244,910	194,030	
	Maximum	7,451,746	3,313,857	

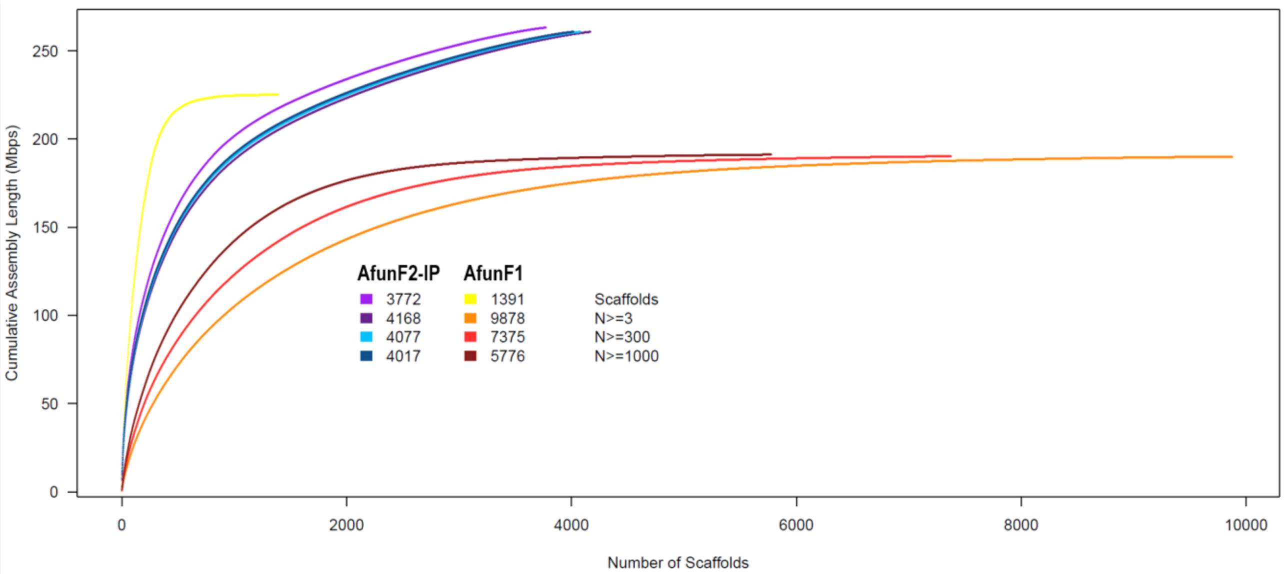

Figure S12. Cumulative scaffold lengths for *Anopheles funestus* AfunF1 and AfunF2-IP assemblies

Cumulative assembly length plots for the reference AfunF1 and the new AfunF2-IP *Anopheles funestus* scaffold-level assemblies. Lengths are summed and plotted from the longest to the shortest scaffold for each assembly. These are replotted for each assembly after splitting scaffolds at consecutive runs of 3, 300, and 1'000 Ns, i.e. effectively de-scaffolding them and slicing at ambiguous or low-quality regions.

[11] Examining collinearity between *Anopheles funestus* assemblies

Robert M. Waterhouse, Livio Ruzzante, Romain Feron

Despite the lack of longer-range scaffolding information from the AfunF2-IP assembly, the scaffolds are nonetheless useful for the purposes of identifying potential adjacencies of the AfunF1 scaffolds through whole genome alignment analyses. The first step towards delineating the order and orientation of *A. funestus* AfunF1 scaffolds along those of the AfunF2-IP assembly was to mask each assembly with a library of anopheline repeats using REPEATMASKER (Smit et al. 2015) and then perform a pairwise LASTZ (Harris 2007) whole genome alignment with default parameters. The resulting alignment blocks were then interrogated with a custom Perl script to define alignment blocks of more than 10 basepairs (bps) from AfunF1 allowing for insertions or deletions of no more than 10 bps in either assembly and requiring AfunF1 genomic regions to be unique (basepairs falling in regions that appeared in more than one alignment block were ignored unless the second-best scoring block scored less than 75% of the best-scoring block, in which case only the best-scoring block was considered). This identified a total of 124'926 links connecting 1'098 AfunF1 scaffolds to 2'845 AfunF2-IP scaffolds with a mean length of 1'234 bps, median of 650 bps, and maximum of 31'044 bps.

Links were then bundled into larger link-regions allowing a maximum of 30 Kbps between links from the same pairs of scaffolds with the same orientations. The largest bundle (by genomic span of the bundled links) for each AfunF1 scaffold was used to define the corresponding AfunF2-IP scaffold and its mapping location was set at the midpoint of the bundle's genomic span on the AfunF2-IP scaffold, thereby ordering and orientating *A. funestus* AfunF1 scaffolds along their corresponding AfunF2-IP scaffolds and producing a final set of 321 alignment-based scaffold adjacencies. Each set of predicted adjacencies, the consensus adjacencies, the physical mapping adjacencies, and the AGOUTI adjacencies were compared with the set of alignment-based scaffold adjacencies (**Table S12**). As the alignments consider scaffolds regardless of whether they were targeted for physical mapping or if they have any annotated orthologues, short un-annotated scaffolds may be ordered and oriented that then result in conflicts with the synteny-based or physical mapping based adjacencies that do not consider such scaffolds. Ignoring short scaffolds (<5 Kbps) or scaffolds with less than 30% aligned sequence reduces the total number of alignment-based scaffold adjacencies by about half to just 154, but this results in additional supported adjacencies being recovered for all the comparison sets, increased support for the synteny-based sets from 14-17.5% to 19-23% and for AGOUTI predictions from 15% to 17% (**Table S12**). The ordered and oriented scaffolds were visualised using CIRCOS (Krzywinski et al. 2009) to display alignments greater than 100 bps, and bundled links greater than 3 Kbps and examine the concordance between the different adjacency predictions (**Figure 5, main text; Figure S13**).

Table S12. Alignment-based adjacency comparisons for *Anopheles funestus*

Comparisons of adjacencies based on alignments of *Anopheles funestus* AfunF1 and AfunF2-IP assemblies with synteny-based, AGOUTI-based, and physical mapping based adjacencies.

Adjacency Set	Adjacencies	Alignment-based with conflicts	Alignment-based with no conflicts	Common to alignment-based & other	Other with conflicts	Other with no conflicts	Additional Supported adjacencies
ADSEQ	331	101	162	58	197	76	18
GOS-ASM	100	26	281	14	66	20	5
ORTHOSTITCH	208	66	223	32	130	46	14
LIBERAL UNION	340	102	164	55	208	77	18
2-WAY CONSENSUS	218	61	223	37	136	45	14
3-WAY CONSENSUS	47	13	303	5	32	10	4
PHYSICAL MAPPING	85	65	237	19	24	42	14
AGOUTI	94	29	278	14	56	24	2

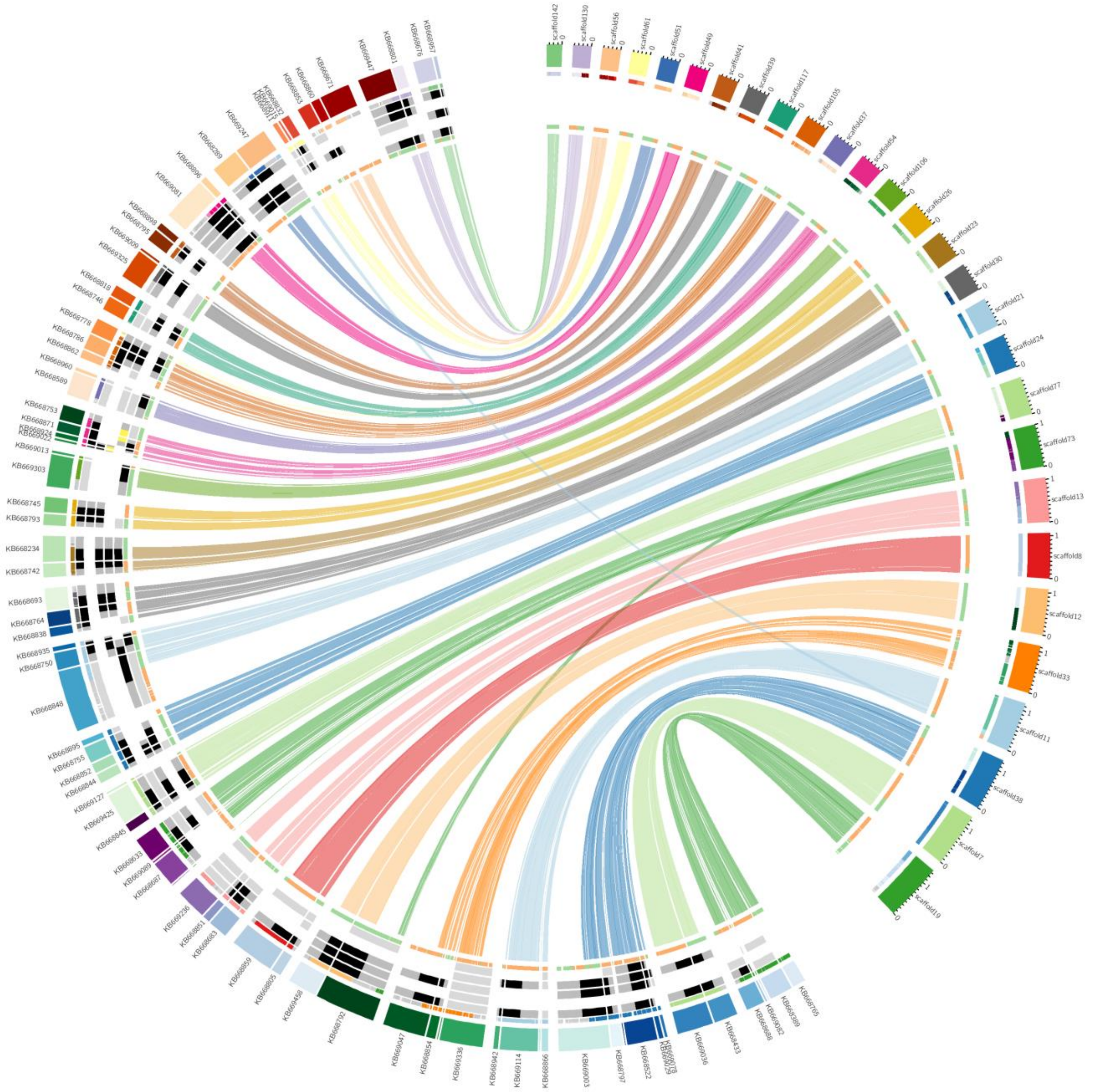

Figure S13. Collinearity between *Anopheles funestus* AfunF1 and AfunF2-IP scaffolds

Anopheles funestus scaffold adjacencies supported by collinearity with the new AfunF2-IP assembly. The plot shows correspondences of AfunF1 scaffolds with AfunF2-IP scaffolds based on whole genome alignment data, with links coloured according to their AfunF2-IP scaffold. Synteny-based adjacency predictions between AfunF1 scaffolds are highlighted with a track showing confirmed neighbours (black), supported neighbours with conflicting orientations (yellow), scaffolds with predicted adjacencies that are not supported by the alignments (light grey) for: from outer to inner tracks, ADSEQ, GOS-ASM, ORTHOSTITCH, physical mapping, and AGOUTI. The innermost track shows alignments in forward (green) and reverse (orange) orientations. The outermost track shows alignments coloured according to the corresponding scaffold in the other assembly (if they align to scaffold not shown on the plot they appear light grey). AfunF1 scaffolds are labelled KB66XXXX and the AfunF2-IP scaffolds are labelled scaffoldX.

The recent availability of a new chromosomal-level assembly for *A. funestus* (Ghurye et al. 2019a) (AfunF3), which used long-reads and Hi-C data from the same *A. funestus* FUMOZ colony, enabled structural comparisons of the original AfunF1 assembly and the AfunF2 superscaffolded assembly with the AfunF3 as a high-quality reference genome. Comparisons were performed with the QUality ASsessment Tool for large genomes (QUAST-LG v5.0.2), which measures completeness and correctness of an assembly against a high-quality reference genome (Mikheenko et al. 2018): 'quast.py AfunF2.fa AfunF1.fa -r AfunF3.fa -o Afun_QUAST -e -t 6 --large --circos -u -m 1'. QUAST-LG aligns query assemblies to a reference assembly and reports differences as misassemblies including relocations (same chromosome), translocations (different chromosomes), and inversions (**Table S13**). QUAST-LG reported totals of 1'980 differences for AfunF1 and an additional 211 differences for AfunF2, with the same proportion of scaffold differences being relocations (both 94%), i.e. mostly putative local rearrangements.

Table S13. QUAST comparisons for *Anopheles funestus*

QUAST-LG comparisons of *Anopheles funestus* AfunF1 and AfunF2 assemblies to the new AfunF3 chromosomal-scale genome assembly.

Assembly	AfunF1	AfunF2	Comments
[1] Genome statistics			
# contigs (>= 0 bp)	1392	1091	As reported in Table 1, main text
# contigs (>= 1000 bp)	1392	1091	
# contigs (>= 5000 bp)	894	601	
# contigs (>= 10000 bp)	793	503	
# contigs (>= 25000 bp)	602	331	
# contigs (>= 50000 bp)	492	240	
Total length (>= 0 bp)	225223604	225253704	Difference due to 301 x 100 Ns added during superscaffolding
Total length (>= 1000 bp)	225223604	225253704	
Total length (>= 5000 bp)	224348209	224394113	
Total length (>= 10000 bp)	223496410	223564719	
Total length (>= 25000 bp)	220556007	220972423	
Total length (>= 50000 bp)	216528867	217621070	
# contigs	1392	1091	
Largest contig	3832769	7691133	AfunF2 superscaffold AFUNE_SS000007 comprises 12 AfunF1 scaffolds
Total length	225223604	225253704	Difference due to 301 x 100 Ns added during superscaffolding
Reference length	210975222	210975222	AfunF3 assembly is slightly shorter than AfunF1 and AfunF2
GC (%)	41.59	41.59	
Reference GC (%)	41.68	41.68	
N50	671960	2051444	As reported in Table 1, main text
NG50	718903	2344827	Like N50, but relative to length of the reference genome, i.e. AfunF3
N75	379841	909998	
NG75	429614	1141772	Like N75, but relative to length of the reference genome, i.e. AfunF3
L50	100	29	The number of scaffolds equal to or longer than N50
LG50	90	26	Like L50, but relative to length of the reference genome, i.e. AfunF3
L75	211	69	The number of scaffolds equal to or longer than N75
LG75	184	58	Like L75, but relative to length of the reference genome, i.e. AfunF3
[2] Misassemblies – differences with AfunF3 reference			
# misassemblies	1980	2191	Number of positions in the contigs (breakpoints) where (i) left flanking sequence aligns over 1 kbp away from right flanking sequence on the reference, or (ii) flanking sequences overlap on more than 1 kbp, or (iii) flanking sequences align to different strands or different chromosomes
# contig misassemblies	467	470	
# c. relocations	401	406	Breakpoints on same chromosome

# c. translocations	37	35	Breakpoints on different chromosomes
# c. inversions	29	29	Flanking sequences align on opposite strands of the same chromosome
# scaffold misassemblies	1513	1721	
# s. relocations	1425	1620	Breakpoints on same chromosome
# s. translocations	65	78	Breakpoints on different chromosomes
# s. inversions	23	23	Flanking sequences align on opposite strands of the same chromosome
# misassembled contigs	475	295	All contigs with any type of a misassembly event
Misassembled contigs length	191997051	212430769	All contigs with any type of a misassembly event
# local misassemblies	5073	5214	Similar to above but the gap or overlap between left and right flanking sequences is less than 1 kbp
# scaffold gap ext. mis.	601	625	
# scaffold gap loc. mis.	3846	3924	
# possible TEs	170	176	
# unaligned mis. contigs	62	52	
# unaligned contigs	257 + 584 part	251 + 361 part	
Unaligned length	21945789	21971307	
Genome fraction (%)	78.551	78.544	Same proportions of AfunF1 and AfunF2 alignable to AfunF3, meaning that this comparison is like-for-like
Duplication ratio	1.227	1.227	
# N's per 100 kbp	15632.54	15643.81	
# mismatches per 100 kbp	1325.21	1335.64	
# indels per 100 kbp	128.41	128.48	
Largest alignment	1463183	1463117	
Total aligned length	168354076	168267075	
NA50	106678	113514	Similar to above, but aligned blocks instead of contigs are considered
NGA50	121583	129205	
NGA75	20556	22319	
LA50	460	434	
LGA50	397	375	
LGA75	1335	1237	

Using 'dot plots' built with D-GENIES (Dot plot large Genomes in an Interactive, Efficient and Simple way) (Cabanettes and Klopp 2018), all AfunF2 scaffolds and superscaffolds that were assigned to chromosomal elements were compared to the newly available chromosomal-level AfunF3 assembly for *A. funestus* (Ghurye et al. 2019a). The AfunF2 scaffolds and superscaffolds were compared to all three chromosomes together (**Figure S14A**), and separately for chromosome X (**Figure S14B** CM012070.1), chromosome 2 (**Figure S14C** CM012071.1), and chromosome 3 (**Figure S14D** CM012072.1). The whole genome comparison showed overall good concordance and a high level of coverage (the main missing region from AfunF2 corresponds to the centromere of chromosome 3). Additionally, while there were evident mismatches that indicate putative translocation events, none of these translocations occurred between chromosomes or chromosome arms.

These comparisons highlighted 50 inversion and/or translocation events between the two assemblies, three fifths of which were local inversions i.e. correct placements but inverted orientations with respect to the AfunF3 reference. For example, on the X chromosome there were just three events: (i) within SS000007 and corresponding to ~7.1M on chromosome X there was an apparent translocation (with no inversion). This corresponds to scaffold KB669181 that was manually placed using PacBio overlap (overriding synteny evidence that did not support this placement). (ii) within SS000029 and corresponding to ~12.5M on chromosome X there was an apparent local inversion (i.e. correct placement but incorrect orientation). This corresponds to scaffold KB669078 that was manually placed using PacBio overlap with no synteny evidence to support or reject this placement. (iii) within SS000019 and corresponding to the very end of chromosome X there was an apparent translocation and inversion event. This corresponds to scaffold KB669082 and this adjacency was predicted by 2-way synteny.

The observation that most differences were small-scale and local, i.e. rearrangements most likely resulting from small inversions, suggests that these could be due to the resolution of Hi-C methods where such small inversions can be frequent due to noise in the data (Ghurye et al. 2019b).

Figure S14A. Dot plot of *Anopheles funestus* AfunF2 scaffolds and AfunF3 chromosomes

Anopheles funestus AfunF2 scaffolds and superscaffolds that were assigned to chromosomal elements compared to their best matching locations in the new AfunF3 chromosomes. The diagonal from bottom left to top right indicate matching contiguously aligned regions. Short regions arranged on the opposite diagonal indicate putative inversions in the AfunF2 scaffolds and superscaffolds with respect to the AfunF3 chromosomes. Regions that are neighbours on the y-axis but not on the x-axis indicate putative translocations in the AfunF2 scaffolds and superscaffolds with respect to the AfunF3 chromosomes.

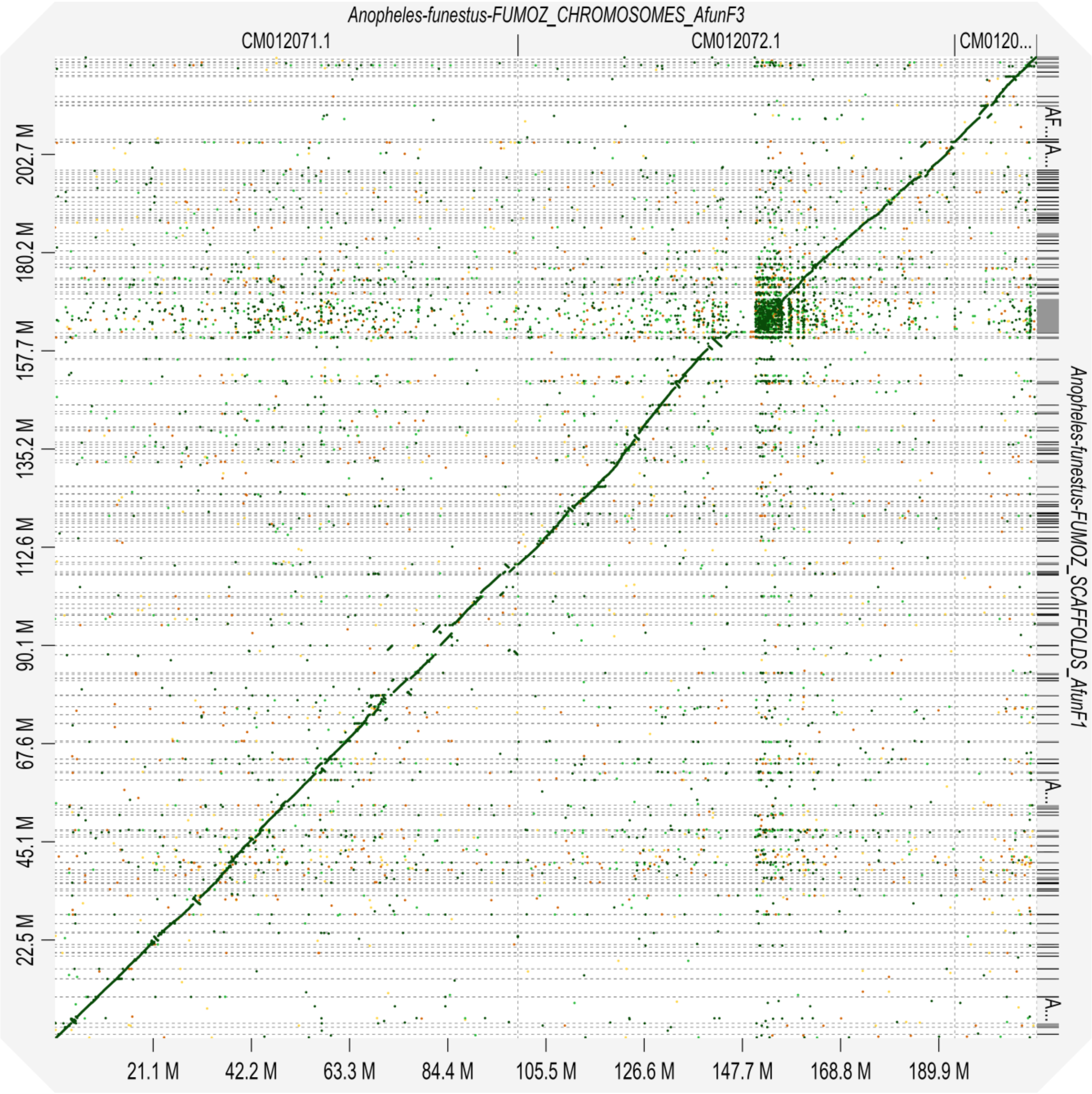

Figure S14B. Dot plot of *Anopheles funestus* AfunF2 scaffolds and AfunF3 chromosome X

Anopheles funestus AfunF2 scaffolds and superscaffolds that were assigned to chromosomal elements compared to their best matching locations in the new AfunF3 chromosome X. The diagonal from bottom left to top right indicate matching contiguously aligned regions. Short regions arranged on the opposite diagonal indicate putative inversions in the AfunF2 scaffolds and superscaffolds with respect to the AfunF3 chromosomes. Regions that are neighbours on the y-axis but not on the x-axis indicate putative translocations in the AfunF2 scaffolds and superscaffolds with respect to the AfunF3 chromosomes.

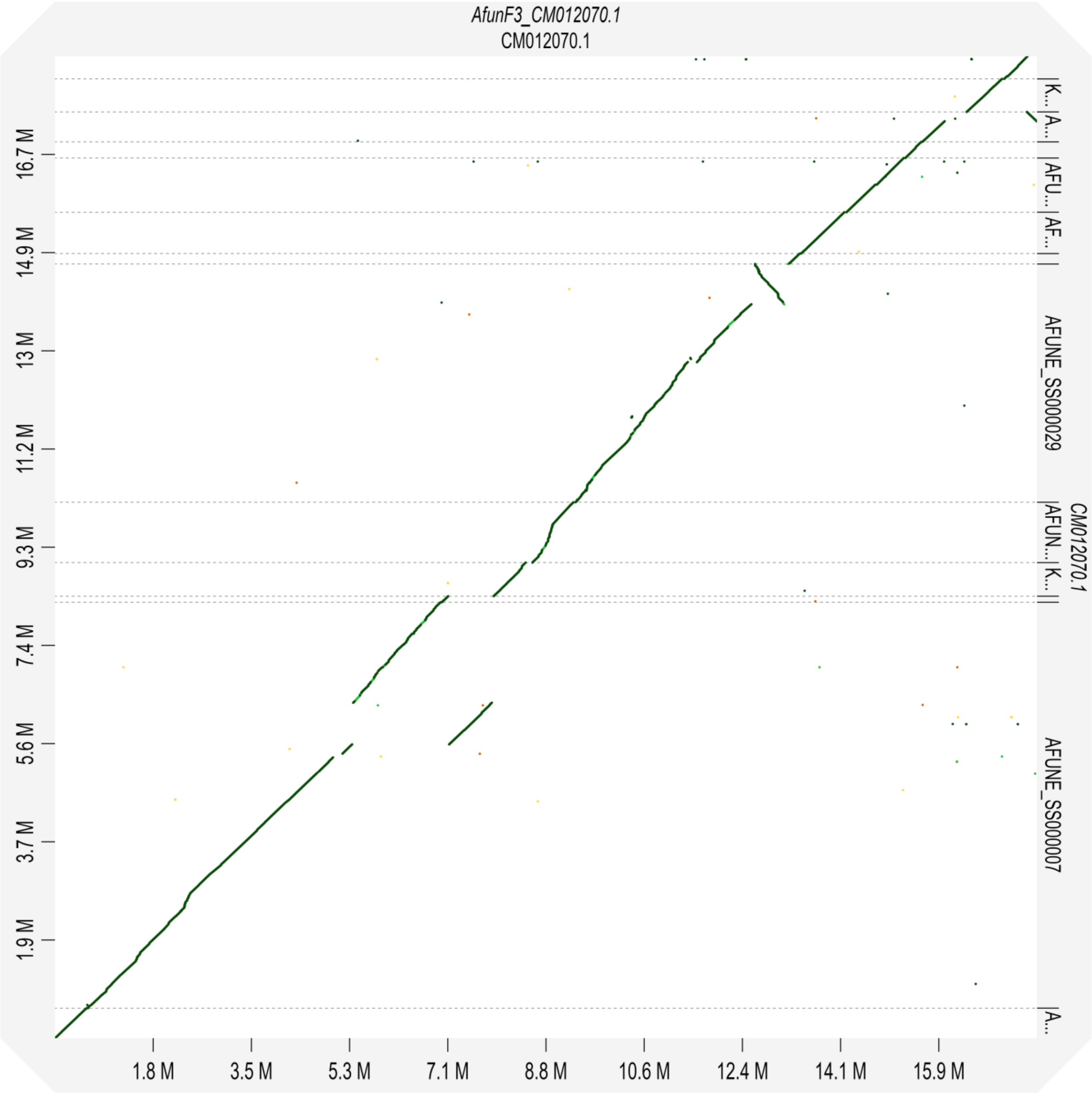

Figure S14C. Dot plot of *Anopheles funestus* AfunF2 scaffolds and AfunF3 chromosome 2

Anopheles funestus AfunF2 scaffolds and superscaffolds that were assigned to chromosomal elements compared to their best matching locations in the new AfunF3 chromosome 2. The diagonal from bottom left to top right indicate matching contiguously aligned regions. Short regions arranged on the opposite diagonal indicate putative inversions in the AfunF2 scaffolds and superscaffolds with respect to the AfunF3 chromosomes. Regions that are neighbours on the y-axis but not on the x-axis indicate putative translocations in the AfunF2 scaffolds and superscaffolds with respect to the AfunF3 chromosomes.

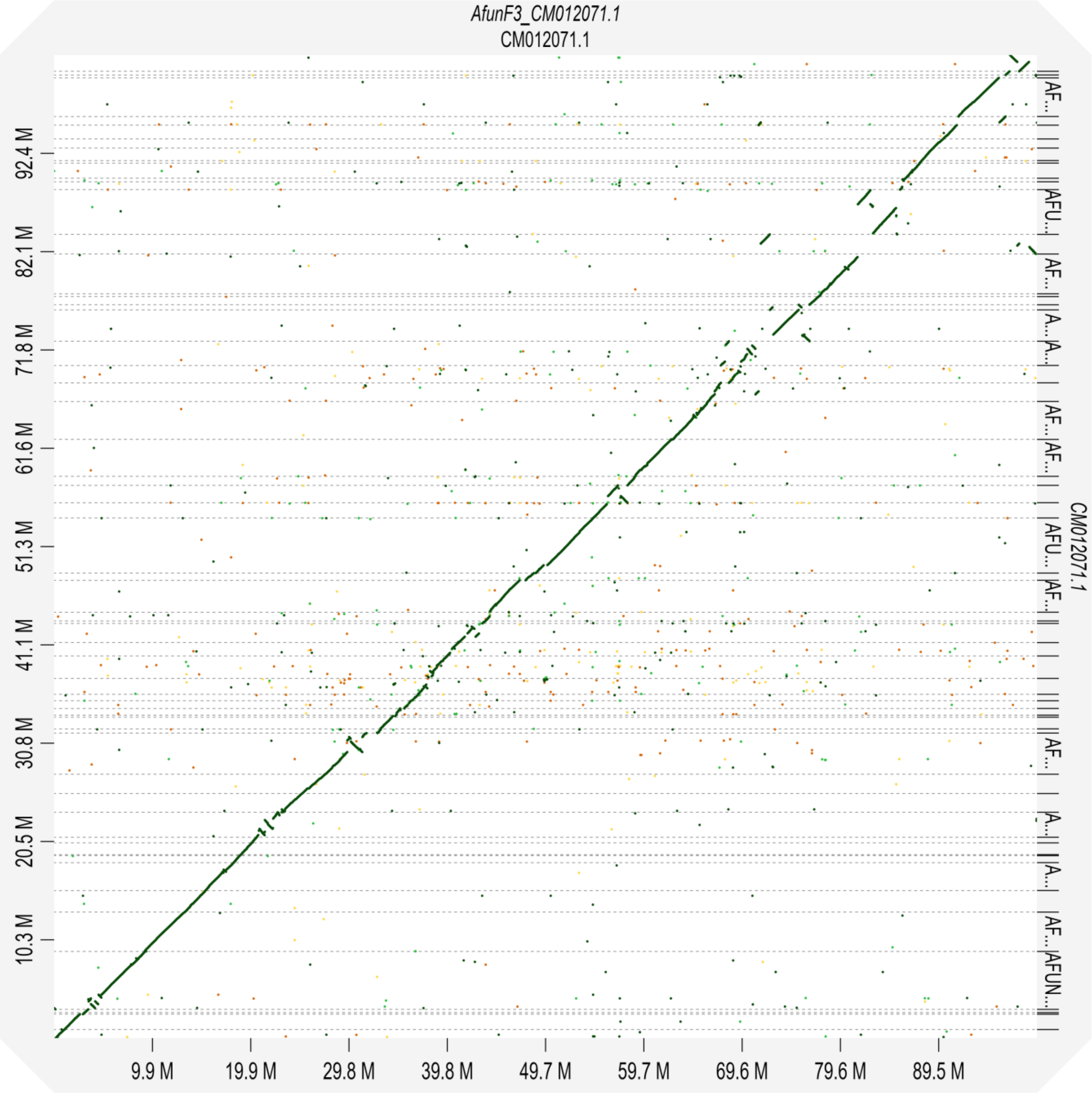

Figure S14D. Dot plot of *Anopheles funestus* AfunF2 scaffolds and AfunF3 chromosome 3

Anopheles funestus AfunF2 scaffolds and superscaffolds that were assigned to chromosomal elements compared to their best matching locations in the new AfunF3 chromosome 3. The diagonal from bottom left to top right indicate matching contiguously aligned regions. Short regions arranged on the opposite diagonal indicate putative inversions in the AfunF2 scaffolds and superscaffolds with respect to the AfunF3 chromosomes. Regions that are neighbours on the y-axis but not on the x-axis indicate putative translocations in the AfunF2 scaffolds and superscaffolds with respect to the AfunF3 chromosomes.

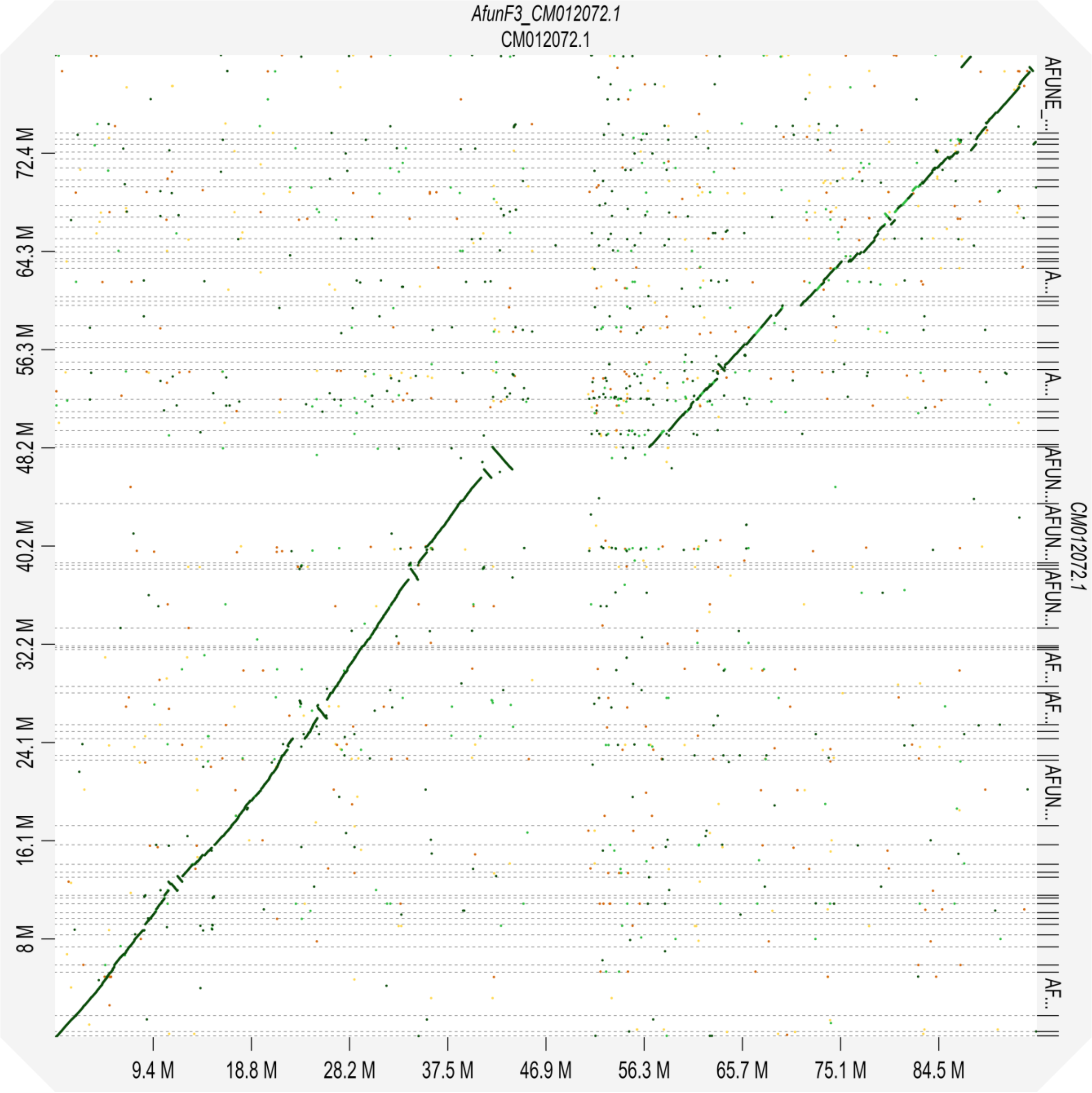

[12] Reconciliation to build the new assemblies

Robert M. Waterhouse, Jiyoung Lee, Livio Ruzzante, Maarten J.M.F. Reijnders, Romain Feron, Daniel Lawson, Gareth Maslen, Igor V. Sharakhov

In order to build the new assemblies for *A. albimanus*, *A. atroparvus*, *A. farauti*, *A. melas*, and *A. merus*, results from the two-way consensus synteny predictions, and AGOUTI and physical mapping data (where available), had to be compared and reconciled with their version 2 reference assemblies. For the published *A. albimanus* AalbS2 assembly, new physical mapping data (also used in this study) was used to improve the assembly by correcting nine misassemblies and anchoring 98% to chromosomes (Artemov et al. 2017). This splitting of the misassembled scaffolds resulted in an increase from 204 AalbS1 scaffolds to 236 AalbS2 scaffolds. The single synteny-based prediction from the two-way consensus set was in agreement with the physical mapping data, as were two of the three adjacencies unique to ORTHOSTITCH, and were therefore already present in the upgraded AalbS2 chromosomal assembly. AGOUTI predicted only two adjacencies, both of which were between very short scaffolds (1'148 bp and 1'012 bp) with no gene annotations and much longer already anchored scaffolds (**Table 2, main text**).

For the published *A. atroparvus* AatrE2 assembly, and later AatrE3, additional physical mapping data (also used in this study) was used to anchor 56 scaffolds (201 Mbps, 89.6% of the assembly) to chromosomes, leaving 1'315 scaffolds unmapped (Artemov et al. 2018a). The *A. melas* AmelC2 assembly was produced from the AmelC1 assembly following the removal of several duplicated scaffolds and regions of scaffolds thereby reducing the number of scaffolds by 52 to 20'229 scaffolds with an unchanged scaffold N50 of 18 Kbps. This affected only 112 scaffolds that were part of 121 adjacencies, and where removed regions made up less than 25% of the original scaffold and they were removed from scaffold ends not involved in any adjacencies then these adjacencies were retained. Thus 95% of AmelC1 adjacencies (97% of scaffolds) were reconciled with the AmelC2 assembly and were used to build the AmelC3 assembly.

The version 2 assemblies for *A. farauti* (AfarF2) and *A. merus* (AmerM2) were derived from re-scaffolding efforts that included the addition of a large-insert 'fosill' sequencing library constructed from high molecular weight DNA, which reduced the numbers of scaffolds from 550 to 310 and 2'753 to 2'027 and increased N50 values from 1'197 Kbps to 12'895 Kbps and 342 Kbps to 1'490 Kbps, respectively. The version 1 assemblies were aligned to the version 2 assemblies using BLAST+ (Camacho et al. 2009) and all scaffolds involved in the synteny-based or AGOUTI-based adjacency predictions were visualised with their corresponding version 2 scaffolds using CIRCOS (Krzywinski et al. 2009). In this way, the predicted adjacencies from version 1 assemblies were assessed to identify adjacencies fully supported by alignments to version 2 scaffolds, e.g. seven *A. farauti* synteny-based two-way consensus set adjacencies confirmed by the alignment with a single AfarF2 scaffold (**Figure S15**). These assessments also identified adjacencies

without support from the version 2 assemblies but which were nonetheless not in conflict (i.e. predicted neighbouring scaffolds that were not joined during the re-scaffolding process), supported neighbours but conflicting orientations, and adjacencies where the arrangements in corresponding version 2 scaffolds precluded the possibility of being neighbours (**Table S14**). The comparisons identified full support for the majority (87% and 82%) of the two-way synteny consensus set adjacencies and unresolvable conflicts for just 5% and 10%, while the AGOUTI-based adjacencies achieved similarly high levels of full support (81% and 67%), but with slightly greater proportions of conflicts.

Table S14. Version 2 assembly reconciliations for *Anopheles farauti* and *Anopheles merus*
Reconciliation of adjacencies for *A. farauti* and *A. merus* with their version 2 assemblies.

Species	Prediction Set	Number of Adjacencies	Fully Supported	Non-Conflicting	Conflicting
Anopheles farauti	Agouti	48	39 (81.2%)	2 (4.2%)	7 (14.6%)
	Two-way synteny	105	91 (86.7%)	9 (8.6%)	5 (4.7%)
Anopheles merus	Agouti	159	106 (66.7%)	20 (12.6%)	33 (20.7%)
	Two-way synteny	372	305 (82.0%)	31 (8.3%)	36 (9.7%)

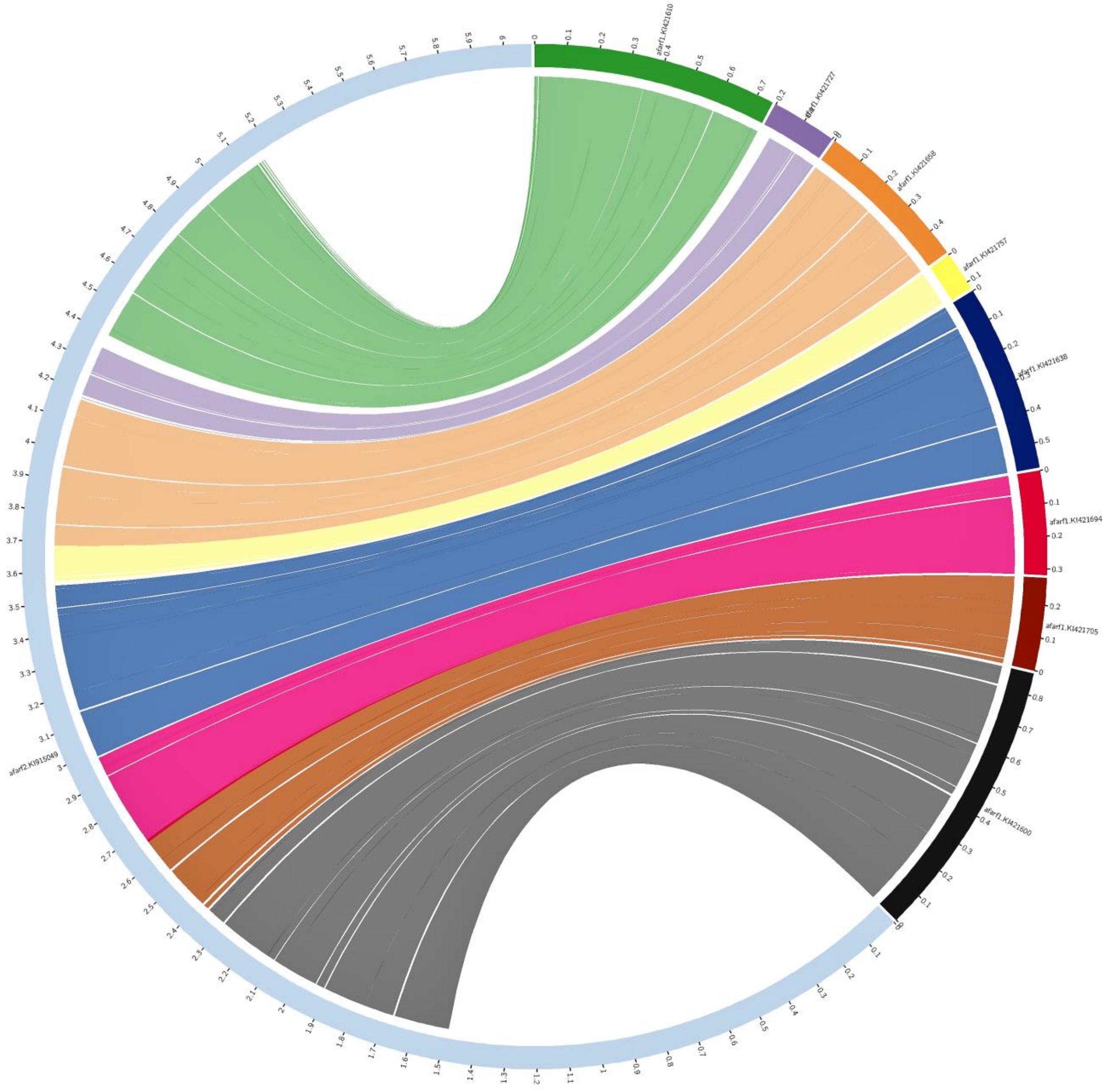

Figure S15. Collinearity between *Anopheles farauti* AfarF1 and AfarF2 scaffolds

Anopheles farauti AfarF1 scaffold adjacencies supported by collinearity with the subsequent AfarF2 assembly. Seven adjacencies from the *A. farauti* synteny-based two-way consensus set predicted the order and orientation of eight AfarF1 scaffolds that are fully supported by the alignment with a single AfarF2 scaffold. Scaffold lengths are shown in increments of 0.1 Mbps. AfarF2 KI915049 aligned with AfarF1 KI421600, KI421705, KI421694, KI421638, KI421757, KI421658, KI421727, KI421610.

New assembly FASTA files and annotation 'lift-over' details

The final lists of pairwise adjacencies and the superscaffolds (**Additional File 6**), with superscaffolds presented in a GRIMM-like format (http://grimm.ucsd.edu/GRIMM/grimm_instr.html) were combined with the VECTORBASE (Release VB-2019-06) assembly sequence data (FASTA format) and assembly annotation data (GFF3 and GTF formats) to produce the new updated assemblies and their corresponding annotations. The adjacencies defined the neighbouring scaffolds that were fused together with an insertion of a stretch of 100 N's to indicate a sequence gap, and with reversed scaffold orientations as required by the relative orientations of the pairwise adjacencies and superscaffolds. Coordinate systems for annotated features were updated to reflect the fusions and insertions to create the superscaffolds with all mapped features. Annotation versions used for lift-overs were: AalbS2.6, AaraD1.11, AatrE3.1, AchrA1.7, AcolM1.8, AculA1.6, AdarC3.8, AdirW1.8, AepiE1.7, AfarF2.6, AfunF1.10, AmacM1.5, AmelC2.6, AmerM2.9, AminM1.8, AquaS1.11, AsinS2.5, AsinC2.2, AsteS1.7, AsteI2.3. For the eight assemblies with chromosome-mapped scaffolds and superscaffolds (**Additional File 7**), AGP (A Golden Path) formatted files were built or updated to assign all finalised scaffolds to chromosomal locations. The authors acknowledge the help provided by Vasily Sitnik at VECTORBASE with the process of updating and submitting the new assemblies and annotations and AGP files.

Chromosome arm assignment using updated assemblies and annotations

Several whole-arm translocations in the anophelines (Neafsey et al. 2015) mean that the five chromosomal elements that make up the X chromosome and the two autosomes correspond to different named chromosome arms in different species (**Table S15**), and thus results are presented as assignments to elements one to five rather than named chromosome arms. Combining orthology data delineated for genes from all 21 assemblies (see section [3] above) and chromosome arm locations for genes from the eight assemblies with chromosomal anchoring data, orthologues of genes on each scaffold were enumerated for each element from each of the eight chromosome-anchored assemblies (**Additional File 2**). To be considered for assignment, the scaffold was required to have a minimum of ten genes with annotated orthologues. The scaffold was then assigned to an element when at least 75% of these orthologues were located on a single element. Confident assignments reported in **Table S2** and main text **Fig. 1** were required to be confirmed by data from at least two species, and conflicting assignments were excluded as they could represent translocation events (assignments with only single-species support or with conflicting species support are reported in **Additional File 2** but flagged as not assigned).

Table S15. Chromosome arm to element correspondences in anophelines

For each of the eight assemblies with chromosome anchoring data, the table presents correspondences between chromosomal elements one to five and the named chromosome arms.

Species	Element 1	Element 2	Element 3	Element 4	Element 5
A. gambiae	X	2R	2L	3R	3L
A. arabiensis	X	2R	2L	3R	3L
A. funestus	X	2R	3R	2L	3L
A. stephensi	X	2R	3L	3R	2L
A. stephensi (Indian)	X	2R	3L	3R	2L
A. sinensis (Chinese)	X	3R	2L	2R	3L
A. atroparvus	X	3R	2L	2R	3L
A. albimanus	X	2R	3L	2L	3R

[13] Software and database availability

ADSEQ: https://github.com/YoannAnselmetti/ADseq-Anopheles-APBC2018, and https://github.com/YoannAnselmetti/DeCoSTAR_pipeline (Anselmetti et al. 2018)

AGOUTI: https://github.com/svm-zhang/AGOUTI (Zhang et al. 2016)

BESST: https://github.com/ksahlin/BESST, (Sahlin et al. 2014)

BLAST+: ftp://ftp.ncbi.nlm.nih.gov/blast/executables/blast+, (Camacho et al. 2009)

BUSCO: https://busco.ezlab.org, (Waterhouse et al. 2018)

CAMSA: https://github.com/compbiol/CAMSA, (Aganezov and Alekseyev 2017)

CIRCOLETTO: https://github.com/infspiredBAT/Circoletto, (Darzentas 2010)

CIRCOS: http://circos.ca, (Krzywinski et al. 2009)

D-GENIES: http://dgenies.toulouse.inra.fr, (Cabanettes and Klopp 2018)

EULERAPE: http://www.eulerdiagrams.org/eulerAPE, (Micallef and Rodgers 2014)

GOS-ASM: https://github.com/aganezov/gos-asm, (Aganezov and Alekseyev 2016)

HISAT: http://www.ccb.jhu.edu/software/hisat/index.shtml, (Kim et al. 2015)

LASTZ: http://www.bx.psu.edu/miller_lab/dist/README.lastz-1.02.00/README.lastz-1.02.00a.html, (Harris 2007)

METASSEMBLER: https://sourceforge.net/projects/metassembler, (Wences and Schatz 2015)

MUSCLE: https://www.drive5.com/muscle, (Edgar 2004)

ORTHODB: https://www.orthodb.org, (Zdobnov et al. 2017)

ORTHOSTITCH: https://gitlab.com/rmwaterhouse/OrthoStitch, (this study)

PRIMERBLAST: https://www.ncbi.nlm.nih.gov/tools/primer-blast, (Ye et al. 2012)

QUAST-LG: https://github.com/ablab/quast, (Mikheenko et al. 2018)

QUIVER: https://github.com/PacificBiosciences/GenomicConsensus, (PacBio's SMRT Analysis software suite)

RASCAF: https://github.com/mourisl/Rascaf, (Song et al. 2016)

RAXML: https://sco.h-its.org/exelixis/web/software/raxml/index.html, (Stamatakis 2014)

REPEATMASKER: http://www.repeatmasker.org, (Smit et al. 2015)

SAMTOOLS: https://github.com/samtools, (Li et al. 2009)

SSPACE: https://www.baseclear.com/services/bioinformatics/basetools/sspace-standard, and https://github.com/nsoranzo/sspace_basic, (Boetzer et al. 2011)

TREERECS: https://gitlab.inria.fr/Phylophile/Treerecs, and https://project.inria.fr/treerecs

VECTORBASE: https://www.vectorbase.org, (Giraldo-Calderón et al. 2015)

DOCKER container

A DOCKER container is provided that packages ADSEQ, GOS-ASM, ORTHOSTITCH, and CAMSA, as well as their dependencies, in a virtual environment that can run on a Linux server, this is available from: https://hub.docker.com/r/mreijnders/synteny/

[14] Main text figure credits

Figure 1. Genomic spans of scaffolds and superscaffolds with and without chromosome anchoring or arm assignments for 20 *Anopheles* assemblies. *Robert M. Waterhouse, Livio Ruzzante, Romain Feron*

Figure 2. Improved genome assemblies for 20 anophelines from synteny-based scaffold adjacency predictions. *Robert M. Waterhouse, Livio Ruzzante, Maarten J.M.F. Reijnders*

Figure 3. Comparisons of synteny-based scaffold adjacency predictions from ADSEQ (AD), GOS-ASM (GA), and ORTHOSTITCH (OS). *Robert M. Waterhouse, Livio Ruzzante*

Figure 4. Scaffold adjacency validations with physical mapping and RNA sequencing data. *Robert M. Waterhouse, Maarten J.M.F. Reijnders*

Figure 5. Whole genome alignment comparisons of selected *Anopheles funestus* AfunF1 and AfunF2-IP scaffolds. *Robert M. Waterhouse*

Figure 6. The *Anopheles funestus* photomap of straightened polytene chromosomes with anchored scaffolds from the AfunF1 and AfunF2-IP assemblies. *Jiyoung Lee, Maria V. Sharakhova, Igor V. Sharakhov*

Figure 7. The *Anopheles stephensi* photomap of straightened polytene chromosomes with anchored scaffolds from the AsteI2 assembly. *Jiyoung Lee, Maria V. Sharakhova, Igor V. Sharakhov*

Supplementary References

Aganezov S, Sitdykova N, Alekseyev MA. 2015. Scaffold assembly based on genome rearrangement analysis. *Computational Biology and Chemistry*.

Aganezov SS, Alekseyev MA. 2017. CAMSA: a tool for comparative analysis and merging of scaffold assemblies. *BMC Bioinformatics* **18**: 496.

Aganezov SS, Alekseyev MA. 2016. Multi-genome scaffold co-assembly based on the analysis of gene orders and genomic repeats. In *Lecture Notes in Computer Science (including subseries Lecture Notes in Artificial Intelligence and Lecture Notes in Bioinformatics)*, Vol. 9683 of, pp. 237–249, Springer, Cham.

Alekseyev MA, Pevzner PA. 2009. Breakpoint graphs and ancestral genome reconstructions. *Genome Res* **19**: 943–957.

Anselmetti Y, Berry V, Chauve C, Chateau A, Tannier E, Bérard S. 2015. Ancestral gene synteny reconstruction improves extant species scaffolding. *BMC Genomics* **16**: S11.

Anselmetti Y, Duchemin W, Tannier E, Chauve C, Bérard S. 2018. Phylogenetic signal from rearrangements in 18 Anopheles species by joint scaffolding extant and ancestral genomes. *BMC Genomics* **19**: 96.

Artemov GN, Bondarenko SM, Naumenko AN, Stegniy VN, Sharakhova M V., Sharakhov I V. 2018a. Partial-arm translocations in evolution of malaria mosquitoes revealed by high-coverage physical mapping of the Anopheles atroparvus genome. *BMC Genomics* **19**: 278.

Artemov GN, Peery AN, Jiang X, Tu Z, Stegniy VN, Sharakhova M V, Sharakhov I V. 2017. The physical genome mapping of Anopheles albimanus corrected scaffold misassemblies and identified interarm rearrangements in genus Anopheles. *G3 Genes|Genomes|Genetics* **7**: 155–164.

Artemov GN, Sharakhova M V, Naumenko AN, Karagodin DA, Baricheva EM, Stegniy VN, Sharakhov I V. 2015. A standard photomap of ovarian nurse cell chromosomes in the European malaria vector Anopheles atroparvus. *Med Vet Entomol* **29**: 230–237.

Artemov GN, Stegniy VN, Sharakhova M V., Sharakhov I V. 2018b. The development of cytogenetic maps for malaria mosquitoes. *Insects* **9**: 121.

Avdeyev P, Jiang S, Aganezov S, Hu F, Alekseyev MA. 2016. Reconstruction of Ancestral Genomes in Presence of Gene Gain and Loss. *J Comput Biol* **23**: 150–164.

Bérard S, Gallien C, Boussau B, Szöllosi GJ, Daubin V, Tannier E. 2012. Evolution of gene neighborhoods within reconciled phylogenies. *Bioinformatics* **28**: i382–i388.

Boetzer M, Henkel C V., Jansen HJ, Butler D, Pirovano W. 2011. Scaffolding pre-assembled contigs using SSPACE. *Bioinformatics* **27**: 578–579.

Cabanettes F, Klopp C. 2018. D-GENIES: dot plot large genomes in an interactive, efficient and simple way. *PeerJ* **6**: e4958.

Camacho C, Coulouris G, Avagyan V, Ma N, Papadopoulos J, Bealer K, Madden TL. 2009. BLAST+: architecture and applications. *BMC Bioinformatics* **10**: 421.

Coluzzi M, Sabatini A, della Torre A, Di Deco MA, Petrarca V. 2002. A polytene chromosome analysis of the Anopheles gambiae species complex. *Science (80-)* **298**: 1415–1418.

Darzentas N. 2010. Circoletto: visualizing sequence similarity with Circos. *Bioinformatics* **26**: 2620–2621.

Duchemin W, Anselmetti Y, Patterson M, Ponty Y, Bérard S, Chauve C, Scornavacca C, Daubin V, Tannier E. 2017. DeCoSTAR: reconstructing the ancestral organization of genes or genomes using reconciled phylogenies. *Genome Biol Evol* **9**: 1312–1319.

Edgar RC. 2004. MUSCLE: multiple sequence alignment with high accuracy and high throughput. *Nucleic Acids Res* **32**: 1792–1797.

Fontaine MC, Pease JB, Steele A, Waterhouse RM, Neafsey DE, Sharakhov I V, Jiang X, Hall AB, Catteruccia F, Kakani E, et al. 2015. Extensive introgression in a malaria vector species complex revealed by phylogenomics. *Science (80-)* **347**: 1258524–1258524.

Ghurye J, Koren S, Small ST, Redmond S, Howell P, Phillippy AM, Besansky NJ. 2019a. A chromosome-scale assembly of the major African malaria vector Anopheles funestus. *Gigascience* **8**.

Ghurye J, Rhie A, Walenz BP, Schmitt A, Selvaraj S, Pop M, Phillippy AM, Koren S. 2019b. Integrating Hi-C links with assembly graphs for chromosome-scale assembly ed. I. Ioshikhes. *PLoS Comput Biol* **15**: e1007273.

Giraldo-Calderón GI, Emrich SJ, MacCallum RM, Maslen G, Dialynas E, Topalis P, Ho N, Gesing S, Madey G, Collins FH, et al. 2015. VectorBase: an updated bioinformatics resource for invertebrate vectors and other organisms related with human diseases. *Nucleic Acids Res* **43**: D707-13.

Harris RS. 2007. Improved pairwise alignment of genomic DNA. The Pennsylvania State University.

Jiang X, Biedler JK, Qi Y, Hall AB, Tu Z. 2015. Complete dosage compensation in Anopheles stephensi and the evolution of sex-biased genes in mosquitoes. *Genome Biol Evol* **7**: 1914–1924.

Jiang X, Peery A, Hall AB, Sharma A, Chen X-G, Waterhouse RM, Komissarov A, Riehle MM, Shouche Y, Sharakhova M V, et al. 2014. Genome analysis of a major urban malaria vector mosquito, Anopheles stephensi. *Genome Biol* **15**: 459.

Kanost MR, Arrese EL, Cao X, Chen Y-RR, Chellapilla S, Goldsmith MR, Grosse-Wilde E, Heckel DG, Herndon N, Jiang HHH, et al. 2016. Multifaceted biological insights from a draft genome sequence of the tobacco hornworm moth, Manduca sexta. *Insect Biochem Mol Biol* **76**: 118–147.

Kim D, Langmead B, Salzberg SL. 2015. HISAT: a fast spliced aligner with low memory requirements. *Nat Methods* **12**: 357–360.

Köster J, Rahmann S. 2012. Snakemake--a scalable bioinformatics workflow engine. *Bioinformatics* **28**: 2520–2522.
Krzywinski M, Schein J, Birol I, Connors J, Gascoyne R, Horsman D, Jones SJ, Marra MA. 2009. Circos: an information aesthetic for comparative genomics. *Genome Res* **19**: 1639–45.
Li H, Handsaker B, Wysoker A, Fennell T, Ruan J, Homer N, Marth G, Abecasis G, Durbin R, Subgroup 1000 Genome Project Data Processing. 2009. The Sequence Alignment/Map format and SAMtools. *Bioinformatics* **25**: 2078–2079.
Micallef L, Rodgers P. 2014. eulerAPE: Drawing Area-Proportional 3-Venn Diagrams Using Ellipses ed. H.A. Kestler. *PLoS One* **9**: e101717.
Mikheenko A, Prjibelski A, Saveliev V, Antipov D, Gurevich A. 2018. Versatile genome assembly evaluation with QUAST-LG. *Bioinformatics* **34**: i142–i150.
Mortazavi A, Schwarz EM, Williams B, Schaeffer L, Antoshechkin I, Wold BJ, Sternberg PW. 2010. Scaffolding a Caenorhabditis nematode genome with RNA-seq. *Genome Res* **20**: 1740–1747.
Neafsey DE, Waterhouse RM, Abai MR, Aganezov SS, Alekseyev MA, Allen JE, Amon J, Arcà B, Arensburger P, Artemov G, et al. 2015. Highly evolvable malaria vectors: the genomes of 16 Anopheles mosquitoes. *Science (80-)* **347**: 1258522–1258522.
Nystedt B, Street NR, Wetterbom A, Zuccolo A, Lin YC, Scofield DG, Vezzi F, Delhomme N, Giacomello S, Alexeyenko A, et al. 2013. The Norway spruce genome sequence and conifer genome evolution. *Nature* **497**: 579–584.
Sahlin K, Vezzi F, Nystedt B, Lundeberg J, Arvestad L. 2014. BESST - Efficient scaffolding of large fragmented assemblies. *BMC Bioinformatics* **15**: 281.
Sharakhov I V, Braginets O, Grushko O, Cohuet A, Guelbeogo WM, Boccolini D, Weill M, Costantini C, Sagnon N, Fontenille D, et al. 2004. A microsatellite map of the African human malaria vector Anopheles funestus. *J Hered* **95**: 29–34.
Sharakhov I V, Serazin AC, Grushko OG, Dana A, Lobo N, Hillenmeyer ME, Westerman R, Romero-Severson J, Costantini C, Sagnon N, et al. 2002. Inversions and gene order shuffling in Anopheles gambiae and A. funestus. *Science (80-)* **298**: 182–185.
Sharakhova M V., Artemov GN, Timoshevskiy VA, Sharakhov I V. 2019. Physical genome mapping using fluorescence in situ hybridization with mosquito chromosomes. In *Methods in Molecular Biology*, Vol. 1858 of, pp. 177–194.
Sharakhova M V., George P, Timoshevskiy V, Sharma A, Peery A, Sharakhov I V. 2014. *Mosquitoes (Diptera), pp. 93-170 in Protocols for cytogenetic mapping of arthropod genomes*.
Sharakhova M V, Xia A, Mcalister SI, Sharakhov I V. 2006. A standard cytogenetic photomap for the mosquito Anopheles stephensi (Diptera: Culicidae): application for physical mapping. *J Med Entomol* **43**: 861–866.
Sharakhova M V, Xia A, Tu Z, Shouche YS, Unger MF, Sharakhov I V. 2010. A physical map for an Asian malaria mosquito, Anopheles stephensi. *Am J Trop Med Hyg* **83**: 1023–1027.
Simão FA, Waterhouse RM, Ioannidis P, Kriventseva E V., Zdobnov EM. 2015. BUSCO: Assessing genome assembly and annotation completeness with single-copy orthologs. *Bioinformatics* **31**: 3210–3212.
Smit A, Hubley R, Green P. 2015. RepeatMasker Open-4.0. <http://www.repeatmasker.org>.
Song L, Shankar DS, Florea L. 2016. Rascaf: Improving Genome Assembly with RNA Sequencing Data. *Plant Genome* **9**: 0.
Stamatakis A. 2014. RAxML version 8: a tool for phylogenetic analysis and post-analysis of large phylogenies. *Bioinformatics* **30**: 1312–1313.
Waterhouse RM, Seppey M, Simão FA, Manni M, Ioannidis P, Klioutchnikov G, Kriventseva E V, Zdobnov EM. 2018. BUSCO applications from quality assessments to gene prediction and phylogenomics. *Mol Biol Evol* **35**: 543–548.
Waterhouse RM, Seppey M, Simão FA, Zdobnov EM. 2019. Using BUSCO to assess insect genomic resources. In *Methods in Molecular Biology*, Vol. 1858 of, pp. 59–74, Humana Press, New York, NY.
Wei Y, Cheng B, Zhu G, Shen D, Liang J, Wang C, Wang J, Tang J, Cao J, Sharakhov I V., et al. 2017. Comparative physical genome mapping of malaria vectors Anopheles sinensis and Anopheles gambiae. *Malar J* **16**: 235.
Wences AH, Schatz MC. 2015. Metassembler: merging and optimizing de novo genome assemblies. *Genome Biol* **16**: 207.
Xia A, Sharakhova M V., Leman SC, Tu Z, Bailey JA, Smith CD, Sharakhov I V. 2010. Genome landscape and evolutionary plasticity of chromosomes in malaria mosquitoes ed. W.J. Murphy. *PLoS One* **5**: e10592.
Ye J, Coulouris G, Zaretskaya I, Cutcutache I, Rozen S, Madden TL. 2012. Primer-BLAST: A tool to design target-specific primers for polymerase chain reaction. *BMC Bioinformatics* **13**: 134.
Zdobnov EM, Tegenfeldt F, Kuznetsov D, Waterhouse RM, Simao FA, Ioannidis P, Seppey M, Loetscher A, Kriventseva E V. 2017. OrthoDB v9.1: cataloging evolutionary and functional annotations for animal, fungal, plant, archaeal, bacterial and viral orthologs. *Nucleic Acids Res* **45**: D744–D749.
Zhang S V., Zhuo L, Hahn MW. 2016. AGOUTI: improving genome assembly and annotation using transcriptome data. *Gigascience* **5**: 31.
Zimin A, Stevens KA, Crepeau MW, Holtz-Morris A, Koriabine M, Marçais G, Puiu D, Roberts M, Wegrzyn JL, de Jong PJ, et al. 2014. Sequencing and assembly of the 22-Gb loblolly pine genome. *Genetics* **196**: 875–890.
